# Positive or U-Shaped Association of Elevated Hemoglobin Concentration Levels with Metabolic Syndrome and Metabolic Components: Findings from Taiwan Biobank and UK Biobank

**DOI:** 10.3390/nu14194007

**Published:** 2022-09-27

**Authors:** Vanessa Joy Timoteo, Kuang-Mao Chiang, Wen-Harn Pan

**Affiliations:** 1 Taiwan International Graduate Program in Molecular Medicine, National Yang Ming Chiao Tung University and Academia Sinica, Taipei City 115, Taiwan; 2 Institute of Biomedical Sciences, Academia Sinica, Taipei City 115, Taiwan

**Keywords:** hemoglobin, iron nutrition status, metabolic syndrome, metabolic disorders, observational study, Taiwanese Han Chinese, European White

## Abstract

Iron overnutrition has been implicated with a higher risk of developing metabolic and cardiovascular diseases, including metabolic syndrome (MetS), whereas iron deficiency anemia exacerbates many underlying chronic conditions. Hemoglobin (Hb) concentration in the blood, which reflects a major functional iron (i.e., heme iron) in the body, may serve as a surrogate of the nutritional status of iron. We conducted sex-specific observational association studies in which we carefully titrated the association between Hb deciles and MetS and its components among the Taiwanese Han Chinese (HC) from the Taiwan Biobank and Europeans of White ancestry from the UK Biobank, representing two large ethnicities. Our data show that at higher-than-normal levels of Hb, increasing deciles of Hb concentration were significantly associated with MetS across all sex subgroups in both ethnicities, with the highest deciles resulting in up to three times greater risk than the reference group [Taiwanese HC: OR = 3.17 (95% CI, 2.75–3.67) for Hb ≥ 16.5 g/dL in men, OR = 3.11 (2.78–3.47) for Hb ≥ 14.5 g/dL in women; European Whites: OR = 1.89 (1.80–1.98) for Hb ≥ 16.24 g/dL in men, OR = 2.35 (2.24–2.47) for Hb ≥ 14.68 g/dL in women]. The association between stronger risks and increasing Hb deciles was similarly observed with all metabolic components except diabetes. Here we found that both the highest Hb decile groups and contrarily the lowest ones, with respect to the reference, were associated with higher odds of diabetes in both ethnic groups [e.g., Taiwanese HC men: OR = 1.64 (1.33–2.02) for Hb ≥ 16.5 g/dL, OR = 1.71 (1.39–2.10) for Hb ≤ 13.5 g/dL; European Whites women: OR = 1.39 (1.26–1.45) for Hb ≥ 14.68 g/dL, OR = 1.81 (1.63–2.01) for Hb ≤ 12.39 g/dL]. These findings confirm that elevated Hb concentrations, a potential indicator of iron overnutrition, may play a role in the pathophysiology of MetS and metabolic components.

## 1. Introduction

Iron is an essential mineral that plays crucial roles in a wide variety of metabolic processes in the human body [1]. It primarily functions in the binding and transporting of oxygen molecules in circulation. It further takes part in immune function, DNA synthesis and cell division, electron transport within cells, and forms an integral part of the vital enzyme systems found in various tissues.

Among the most common diseases in humans are those linked to iron metabolism and homeostasis, ranging from anemia (i.e., commonly caused by iron deficiency) to hemochromatosis (i.e., iron overload) [2]. Anemia, clinically described as a condition wherein the number and size of red blood cells (RBCs) and their oxygen-carrying capacity become insufficient to meet physiological needs, remains to be a global public health problem [3]. It afflicted 1.62 billion individuals worldwide based on the published data of the World Health Organization (WHO) Global Database in 2008 [4]. More recently, in 2019, anemia was reported to globally affect 39.8% of children and 29.9% of women at reproductive age [5].

Meanwhile, epidemiological studies have implicated that iron overnutrition and elevated iron levels are associated with higher risk of adverse metabolic and cardiovascular outcomes [6]. Metabolic syndrome (MetS) is a disease entity characterized by the clustering of insulin resistance, impaired glucose tolerance, dyslipidemia, hypertension, and central obesity–all of which can lead to major cardiovascular events [7]. A global epidemic of MetS has been recognized for some time as a result of genetic susceptibility and lifestyle changes associated with modernization and urbanization (e.g., poor dietary quality, sedentary lifestyle, and physical inactivity). There has been a continuous increase in the prevalence of MetS in both men and women in all age groups on a global scale [7]. A 25.5% prevalence of MetS among Taiwanese Han Chinese (HC) was reported in the Nutrition and Health Survey in Taiwan (NAHSIT) from 2005 to 2008, with a lower proportion of women affected than men aged below 45 years, but with a higher proportion of women affected than men for those aged 45 years and over [8,9]. Similarly, in the United Kingdom, a MetS prevalence of 24.3% (i.e., one in four adults) was reported across several European cohorts [10].

Oxidative stress is known to be involved in the pathophysiology of many chronic non-communicable diseases, including MetS. Although there are other processes contributing to their onset, oxidative stress leads to the development of prolonged inflammatory state and further complications [11]. Iron deficiency and overload both influence the redox states. Nonetheless, the exact mechanisms as to how iron contributes to metabolic derangements are yet to be fully elucidated. It was proposed long before, through the ‘iron hypothesis’, that iron depletion protects against heart disease whereas high levels of body iron stores promote cardiovascular and metabolic diseases [12]. To date, most evidence supports that iron is a powerful pro-oxidant that in excess can cause cellular damage by producing reactive oxygen species in different tissues of the body, which may eventually lead to atherosclerotic events [13].

Iron, being an essential micronutrient, comes entirely from the diet. However, the bioavailability of iron depends on the dietary matrix, which, in turn, is constituted by the major dietary patterns found in a population [1,14]. Heme iron from meat, poultry, and fish is a superb source of iron compared to the non-heme iron from plant sources. Red meat consumption is known to contribute to the storage of excess iron in the body. On the contrary, non-heme iron, despite its lower bioavailability, was shown to contribute more to iron nutrition due to its abundant quantity in the diet [15].

Approximately two-thirds (65%) of iron in the human body are integrated in the protein hemoglobin (Hb) in circulating RBCs. The four heme units of Hb contain an iron cation (Fe^2+^) that switches redox states upon the binding and release of oxygen from the lungs to cells and of carbon dioxide from the cells to the lungs [16]. Therefore, Hb concentration in the blood may serve as a surrogate of iron nutrition status as it reflects a major functional iron in the body (i.e., heme iron) [17]. Nonetheless, Hb concentration may be affected by other prevailing micronutrient deficiencies, acute or chronic infections, inflammation, and disorders that alter RBC metabolism [18]. Hb is a good iron marker when the body iron store is low, whereas at the higher end of iron spectrum, variations in Hb concentration may be due to other nutritional or non-nutritional factors including autoimmune disease [19]. It is further important to note that other biomarkers (i.e., serum ferritin, transferrin saturation, etc.) should be considered with respect to the storage and transport of iron in humans.

There have been on-going controversies in human epidemiological studies as to whether the disruption of iron homeostasis that leads to elevated Hb levels may result in higher risks of metabolic disorders. Hence, we conducted sex-specific association studies to determine and validate the relationship of iron in excess with the risks of MetS and its individual components. We carefully titrated the association between Hb deciles and MetS, central obesity, hypertension (HTN), diabetes (i.e., type 2 diabetes or T2D), dyslipidemia (DLP), and gout. Gout was additionally included as an outcome due to the emerging evidence that suggests its role in the pathophysiology of MetS [20]. We utilized two, large population-based cohorts, the Taiwan Biobank (TWB) and UK Biobank (UKB), to represent the distinct ethnicities of East Asians and Caucasians, respectively. Our findings then confirm the role of elevated iron status, as demonstrated by increasing Hb concentrations, in the pathophysiology of MetS and metabolic outcomes in both the Taiwanese HC and European Whites. Intriguingly, we observed that both low and high levels of Hb contribute to the pathogenesis of T2D, whereas within normal levels, Hb confers protective effects. These findings will help to further our understanding on the role of iron dysregulation in human health and better identify at-risk individuals; hence, preventing further complications associated with the MetS and other cardiometabolic outcomes–ultimately leading to the improvement of public health in the future.

## 2. Materials and Methods

### 2.1. Study Design, Study Populations, and Ethical Considerations

We conducted cross-sectional observational studies utilizing two population-based cohorts, the Taiwan Biobank and the UK Biobank. The TWB is a large-scale collection of data on the genomic profiles, lifestyle, and environmental exposure history, and long-term health outcomes of the Han Chinese population in Taiwan. It has been established to elucidate the interrelationship between genetic and environmental factors in disease onset and progression [21]. The UKB, on the other hand, is a large prospective study of more than 500,000 individuals residing in the UK, whose extensive phenotype information, genotypes, and details on assessment and follow-ups have been reported in detail elsewhere [22]. Information on the TWB and UKB Projects can be accessed at https://www.twbiobank.org.tw/ (accessed on 6 June 2022) and https://www.ukbiobank.ac.uk/ (accessed on 6 June 2022).

The TWB cohort initially comprised 67,515 participants (20,764 men and 46,751 women), aged from 30 to 70 years old at the time of recruitment. These were community-dwelling and non-handicapped individuals during assessment. In order to maximize the power of the TWB study, we included all subjects at first, regardless of age. We excluded subjects who had self-reported renal/kidney failure, been diagnosed with cancer, or had missing data on Hb concentration.

From a total of 502,536 UKB participants (229,134 men; 273,402 women) aged from 40 to 70 years old during assessment (UKB data field 21003), we first selected participants who identified themselves as White (i.e., British, Irish, or any other White background) (field 21000) and were born in the UK (field 1647). We removed subjects who had inconsistent reported and genetic sex (fields 31 and 22001), were pregnant during data collection (field 3140), had been diagnosed with any form of cancer (fields 134 and 20001), and had self-reported renal/kidney failure, hereditary/genetic hematological disorder, clotting disorder/excessive bleeding, acquired immunodeficiency syndrome (HIV/AIDS), tuberculosis, or tropical and travel-related infections (field 135). These conditions are known to affect erythropoiesis and, thus, may influence the Hb levels of an individual [18]. We further excluded subjects with fasting time of 0 or beyond 24 h (field 74) and those with missing Hb data (field 30020). File S1 provides the list of variables and data fields used in the UKB study.

Upon further exclusion of subjects with extreme Hb values, a total of 67,237 Taiwanese HC (20,670 men and 46,567 women) were included in the analyses. On the other hand, a final population of 386,477 Europeans of White ancestry (182,048 men and 204,429 women) were analyzed.

This study has been carried out in accordance with The Code of Ethics of the World Medical Association (Declaration of Helsinki). Study protocols were evaluated and approved by the Institutional Review Board of Academia Sinica, with reference number AS-IRB 02. The UKB Project has been given ethical clearance by the UK National Health Service’s National Research Ethics Committee with reference number 11/NW/TWB and UKB participants gave duly signed written informed consents prior to the conduct of data collection. All data obtained were treated with the utmost confidentiality.

### 2.2. Blood and Data Collection

Venous blood samples were drawn from the TWB and UKB participants by qualified and trained medical researchers for hematological and biochemical assessments. Blood samples collected in EDTA (Ethylenediaminetetraacetic acid) vacutainer tubes were analyzed within 24–48 h after blood draw using automated, quality control-checked clinical hematology analyzers. Hemoglobin is one of the hematological traits measured at baseline recruitment, where anemia is defined by a Hb level <13.0 g/dL in men and <12.0 g/dL in women [23]. Serum and plasma were then separated from the whole blood samples for the measurement of a wide range of biochemical markers in the TWB and UKB. Glucose (GLU), triglyceride (TG), cholesterol (TC), high-density (HDL-C), and low-density (LDL-C) lipoprotein-associated cholesterol, and uric acid (UA) were measured via enzymatic assays using routinely calibrated clinical chemistry analyzers, whereas glycated hemoglobin (HbA1c) was determined by a high-performance liquid chromatography method. In the UKB, however, blood biochemistry was based on a random and non-fasting state of participants.

Standard procedures were followed in collecting anthropometric and blood pressure readings. Briefly, weight was recorded using a platform weighing scale whereas standing height was measured using a microtoise or medical measuring rod. Body mass index (BMI) was calculated as weight in kg divided by the square of height in m (kg/m^2^). Waist (WC) and hip circumferences were measured at the approximate midpoint between the lowest rib bone and super iliac point and the widest part of the hips, respectively, with a tape measure placed parallel to the floor at the end of a relaxed expiration of participants while standing. Waist-hip ratio (WHR) was simply calculated as waist circumference divided by the hip circumference. Systolic (SBP) and diastolic blood pressure (DBP) levels were obtained using automated sphygmomanometer following stringent protocol. All measurements were taken twice.

The socio-demographic profile, behavioral risk factors such as smoking and alcohol consumption, physical activity, and other health-related information about the participants were obtained through a face-to-face interview in the TWB. On the other hand, an extensive array of lifestyle, environmental, health, and medical data among UKB participants were collected via a touchscreen questionnaire, followed by a verbal interview for some variables that required verification (i.e., type of prescription medications).

### 2.3. Definition and Ascertainment of Metabolic Outcomes

Metabolic syndrome and its associated components (i.e., central obesity, hypertension, diabetes, dyslipidemia, and gout) are the metabolic outcomes, as dichotomous variables, in this study. We defined outcomes based on self-reports of physician-diagnosed health conditions by the participant and the widely used cut-offs for WC, SBP/DBP, GLU, TG, HDL-C, and UA. In the UKB, outcomes were additionally classified using data on medications and hospital episode statistics following the International Classification of Diseases 9th (ICD9) and 10th (ICD10) revisions. File S2 summarizes the disease definitions based on ICD and self-reported fields, whereas File S3 lists the prescription medications in the ascertainment of HTN, T2D, DLP, and gout in the UKB.

The definition of central or abdominal obesity was adopted from the International Diabetes Federation (IDF) criteria: a WC > 90 cm in men or >80 cm in women for East Asians and a WC > 94 cm in men or >80 cm in women for Europeans [24,25]. An SBP/DBP reading of ≥140/≥90 mm Hg classified hypertension in the TWB and UKB following the Taiwan Society of Cardiology/Taiwan Hypertension Society and the European Society of Cardiology/European Society of Hypertension criteria, respectively [26,27]. Type 2 diabetes in TWB was defined by a high fasting blood glucose (FBG) level ≥ 126 mg/dL [28] or a glycated hemoglobin ≥ 6.5% [29]. To convert FBG in mg/dL to SI units, multiply by 18 mmol/L [30]. HbA1c in mmol/mol is calculated as 10.93 × (HbA1c in %) − 23.50 [31]. In the ascertainment of T2D in UKB subjects, we followed the algorithms proposed by Eastwood and team, where GLU ≥ 11.1 mmol/L or HbA1c ≥ 48 mmol/mol (6.5%) captured hyperglycemia from blood tests even on a non-fasting state [32]. Hypertriglyceridemia, hypercholesterolemia, high LDL-cholesterol levels, and low HDL-cholesterol levels were classified in the TWB and UKB using the following cut-offs: TG ≥ 200 mg/dL (≥2.30 mmol/L), TC ≥ 240 mg/dL (≥6.20 mmol/L), LDL-C ≥ 160 mg/dL (≥4.10 mmol/L), and HDL-C < 40 mg/dL (<1.00 mmol/L) in men or <50 mg/dL (<1.30 mmol/L) in women [33,34]. Dyslipidemia in this study is described as a combination of either hypertriglyceridemia or low levels of HDL-C. TG in mg/dL is converted to SI units by multiplying by 0.01129 mmol/L, whereas TC, LDL-, and HDL-C are multiplied by 0.02586 mmol/L [35]. Lastly, gout was ascertained from a hyperuricemic level of >7.0 mg/dL (>416.0 μmol/L) in men or >6.0 mg/dL (>357.0 μmol/L) in women [36,37]. UA from mg/dL to μmol/L is computed using a conversion factor of 1 mg/dL = 59.48 μmol/L [38].

The definition of metabolic syndrome was mainly based on the NCEP ATP III criteria [39], whereas the WC cut-off point for Asians from IDF [24] was used in analyzing the Taiwanese HC data. In the NCEP ATP III classification, MetS was present upon meeting at least three of the following component risk factors: (1) WC > 102 cm in men or >88 cm in women; (2) TG ≥ 150 mg/dL (≥1.70 mmol/L) or taking triglyceride-lowering drugs; (3) HDL-C < 40 mg/dL in men or <50 mg/dL in women or taking statins or other medicines for high cholesterol; (4) blood pressure ≥130/≥85 mmHg or current use of anti-hypertensive drugs; and (5) FBG ≥ 100 mg/dL (≥5.6 mmol/L) or current use of anti-hyperglycemic drugs. The ethnic-specific criteria for central obesity in IDF (i.e., for Taiwanese HC) is WC > 90 cm in men and >80 cm in women.

### 2.4. Data Processing and Statistical Analyses

Data were analyzed using SAS v.9.4. The criteria for statistical significance were a *p*-value of <0.05.

For describing the characteristics of participants, continuous variables were expressed as mean ± S.D., whereas categorical characteristics were in counts and percentage values. The normal distribution of quantitative parameters was assessed, and extreme outliers were removed. We excluded subjects who were more than ± 5 S.D. from the sex-specific Hb means per ethnic group.

For comparing the risks of MetS and associated components, we categorized subjects according to deciles of increasing hemoglobin concentration. The decile with the lowest outcome prevalence served as the reference. Men and women were analyzed separately. Trends in quantitative characteristics and prevalence across Hb deciles were respectively tested by linear regression and the Cochran–Armitage trend test.

Multivariate logistic regression models were used to estimate the ORs and CIs of MetS and its individual components across Hb deciles. Age and age-squared were adjusted in the first model. Smoking status and alcohol consumption were further adjusted as covariates in the second model. The final model was fully adjusted for physical activity level, highest educational attainment, and comorbidities (i.e., for individual components of MetS). Fasting time was included in the UKB analyses to adjust for random/non-fasting blood biochemistry collection. Menopausal status was also added as a covariate in the women subgroups, wherein subjects were categorized as either non-menstruating, unsure of their menopausal status (i.e., had hysterectomy), or had age-at-menopause <44, 45–49, 50–54 (reference), or >55 years. We further adjusted for ethnic-specific BMI category groups [40,41] in all models when testing the associations between Hb and HTN, T2D, DLP, and gout.

In the UKB, the final categorization of smoking status (i.e., never smoked, stopped smoking, occasionally smoking, and currently smoking) was derived from smoking status (field 20116) and current tobacco smoking (field 1239). Alcohol drinking status (i.e., never drank, stopped drinking, occasionally drinking, and currently drinking) was similarly recoded from alcohol drinker status (field 20117) and alcohol intake frequency (1558). The highest educational qualification in the UK (field 6138) was recategorized into four levels as none; O-levels, CSEs, or equivalent; A-levels, NVQ/HND/HNC or equivalent, or other professional qualifications; and college or university degree holder.

## 3. Results

### 3.1. Characteristics of Taiwanese Han Chinese and European White Cohorts

The characteristics of TWB and UKB cohorts by sex and increasing Hb deciles were presented in detail in Tables S1 and S2, respectively. A total of 67,237 Taiwanese Han Chinese (20,670 men; 46,567 women) from the TWB and 386,477 European Whites (182,048 men; 204,429 women) from the UKB were included in the analyses. The mean ages were around 50 years old for male and female TWB participants and around 56–57 years for UKB counterparts, respectively. Around half of the Taiwanese HC women had already entered menopause (51.3%) during assessment, with a mean age-at-menopause of 49.4 ± 5.0 years. A large proportion of European women subjects also had menopause (60.6%), with a mean menopausal age of 49.8 ± 5.1 years.

The mean Hb levels of men subgroups were found to be similar in both populations (15.0 g/dL), whereas European women had a slightly higher mean Hb (13.5 ± 0.93 g/dL) than Taiwanese HC women (13.0 ± 1.3 g/dL). The interquartile ranges of Hb concentration were wider in the Taiwanese HC (14.4–15.8 g/dL in men; 12.5–13.8 g/dL in women) than in Europeans (14.4–15.7 g/dL in men; 12.9–14.1 g/dL in women). In both ethnic groups, men had higher mean measurements in all anthropometric, biochemical, and clinical parameters than women, except TC and LDL-C.

Majority of the subjects were never-smokers. In particular, nearly all of the Taiwanese HC women had never smoked a cigarette. Taiwanese HC were mostly occasional-drinkers whereas larger percentages of Europeans were current-drinkers. More than half of the Taiwanese HC did not regularly exercise. Meanwhile, majority of European men and women engaged in high and moderate physical activities, respectively. For both ethnic groups, however, a general increase in the proportion of current-smokers, current-drinkers, and those with no regular exercise (or had low physical activity levels) were observed with increasing Hb deciles. For instance, 32.9% of Taiwanese HC men and 14.1% of European men were current-smokers in the highest Hb deciles as compared to the 16.3% and 7.4%, respectively, in the lowest deciles. Lastly, at least half of the Taiwanese HC reached university or post-graduate studies, whereas relatively lower percentages of Europeans obtained a degree. Across increasing Hb concentration, we also observed a general increase in percentages of those with no formal education or a decrease in percentages of degree-holders.

### 3.2. Prevalence Rates of Metabolic Syndrome and Metabolic Components and Associations with Increasing Hemoglobin Deciles among Taiwanese Han Chinese and European Whites

There were significant differences in the prevalence rates of MetS and metabolic components by ethnic group (Tables S3 and S4 show by sex and Hb deciles the frequency distributions of outcomes based on measured health indicators; Tables S5 and S6 present the frequencies of cases based on self-reported data; Table S7 provides the frequencies from hospital in-patient records in the UKB).

Europeans were observed to have a higher mean weight, BMI, WC, SBP, and DBP than the Taiwanese HC counterparts. The mean TG, TC, and LDL-C of Europeans were also higher than the Taiwanese HC (i.e., TG: 1.98 mmol/L in European men versus 1.57 mmol/L in Taiwanese HC men; 1.55 mmol/L in European women versus 1.17 mmol/L in Taiwanese HC women). Conversely, the mean glucose, HbA1c, and UA levels of Taiwanese HC were found larger than the Europeans, despite a non-fasting blood collection among the latter (i.e., glucose: 5.5 mmol/L in Taiwanese HC men versus 5.1 mmol/L in European men; 5.2 mmol/L in Taiwanese HC women versus 5.1 mmol/L in European women).

Overall, we present that among the MetS components, central obesity was consistently highly prevalent among the Taiwanese HC (44.8%) and European Whites (56.7%), followed by dyslipidemia (i.e., combination of either hypertriglyceridemia or low levels of HDL-C) (34.3%; 51.9%), hypertension (23.9%; 56.6%), gout (20.2%; 14.4%), and type 2 diabetes (10.2%; 7.2%) (Tables S8 and S9).

We noted the highly significant increasing trends in the prevalence rates of MetS and other metabolic components with increasing deciles of Hb concentration among Taiwanese HC and European Whites (*p* < 0.0001) (Tables S3–S7). However, on the other side of the Hb distribution, we also observed that the Hb decile groups which comprised mostly the anemic subjects (i.e., D1 in Taiwanese HC men and Europeans; D1 and D2 in Taiwanese HC women) had higher prevalence rates of MetS, central obesity, hyperglycemia, low HDL-C, and hyperuricemia than those in the reference deciles. The elevation of rates is strong for hyperglycemia but modest for others.

**Figure 1:**
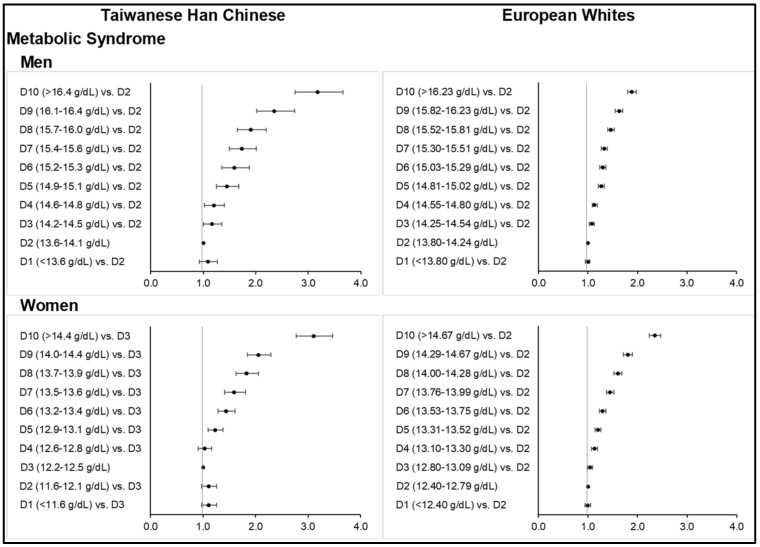
Odds ratios of metabolic syndrome across increasing deciles of hemoglobin concentration among Taiwanese Han Chinese and European Whites (top: men, bottom: women). Final models adjusted for age, age-squared, smoking status, alcohol consumption, physical activity level, and highest educational attainment, as well as menopausal status in women.

**Figure 2:**
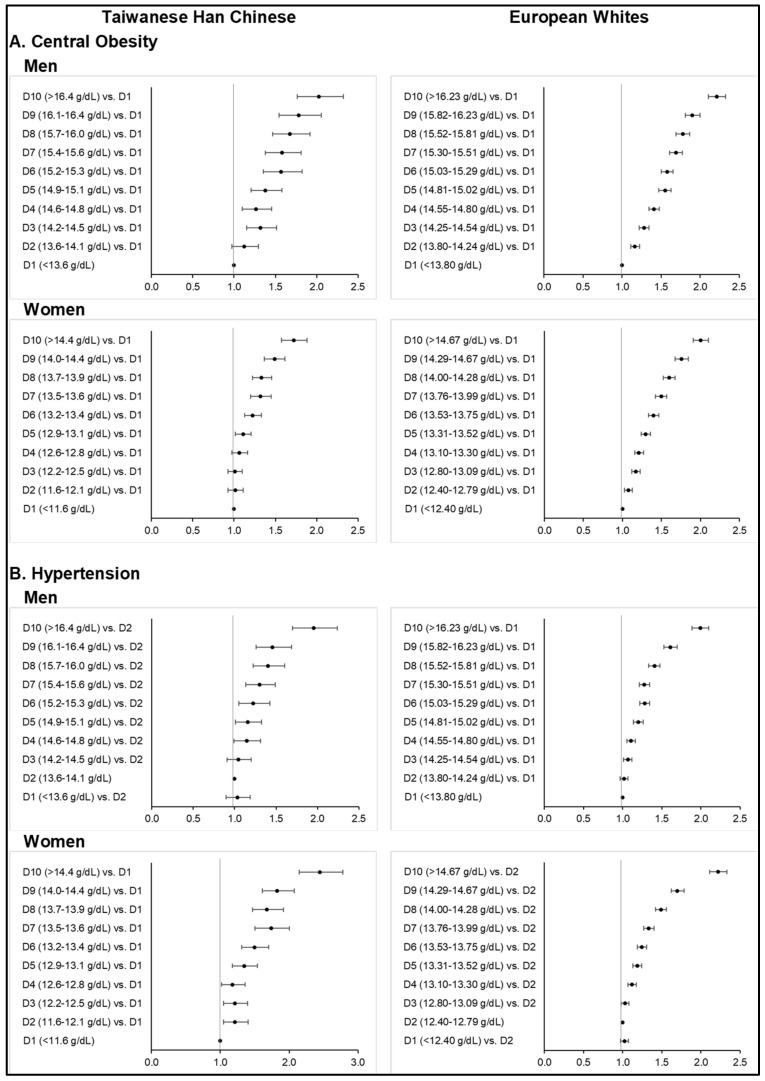

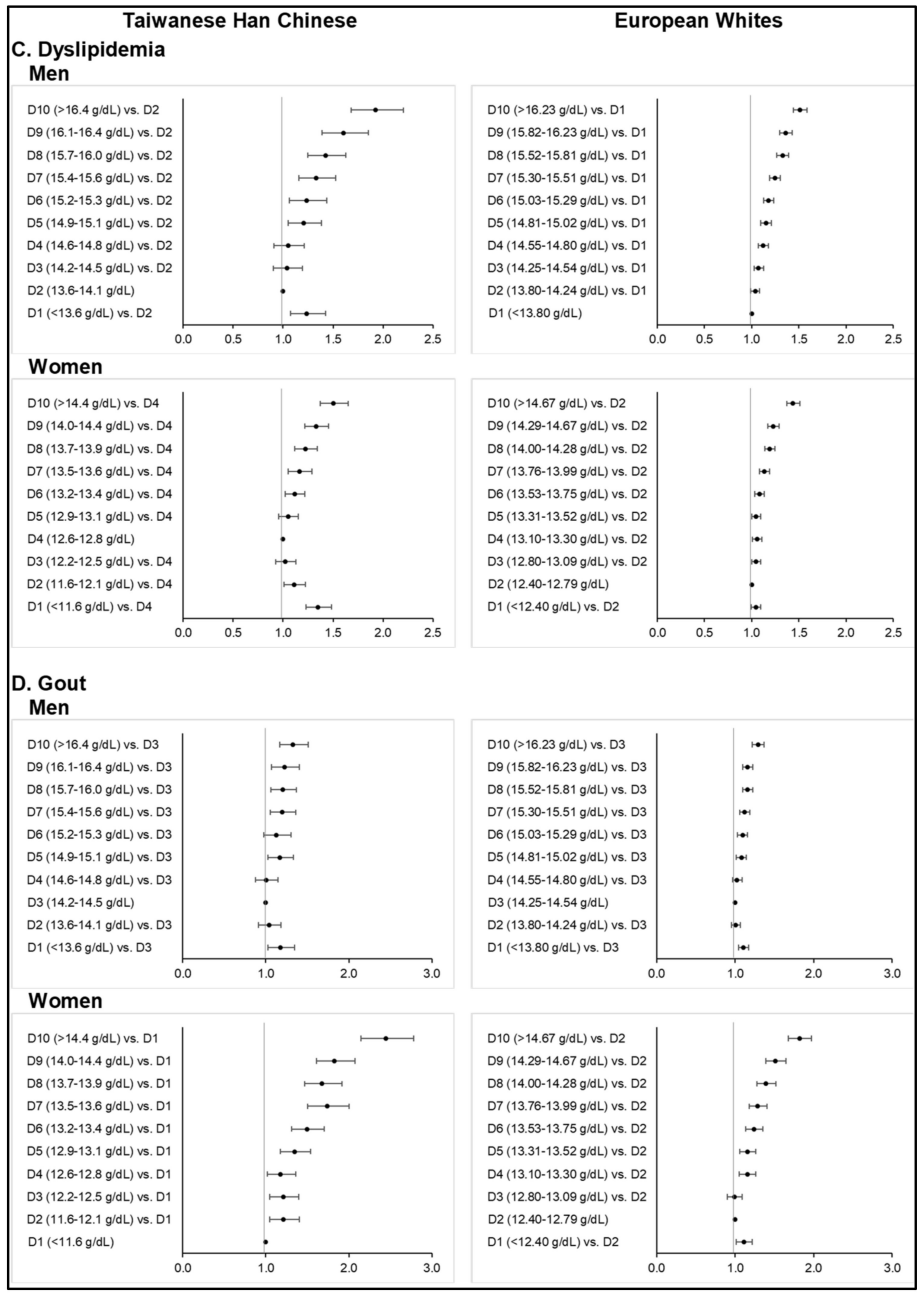
Odds ratios of central obesity, hypertension, dyslipidemia, and gout across increasing deciles of hemoglobin concentration among Taiwanese Han Chinese and European Whites (top: men, bottom: women). Final models adjusted for age, age-squared, smoking status, alcohol consumption, physical activity level, highest educational attainment, and comorbidities, as well as menopausal status in women.

**Figure 3:**
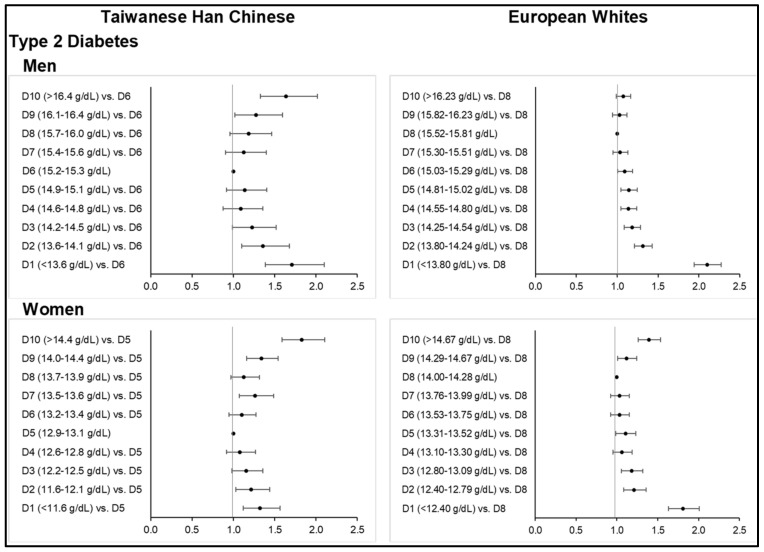
Odds ratios of diabetes across increasing deciles of hemoglobin concentration among Taiwanese Han Chinese and European Whites (top: men, bottom: women). Final models adjusted for age, age-squared, smoking status, alcohol consumption, physical activity level, highest educational attainment, and comorbidities, as well as menopausal status in women.

Consistent with the above observations, increasing Hb was found significantly associated with the increasing odds of MetS (Figure 1; Tables S10 and S11) and its metabolic components (Figure 2; Tables S12–S21) across all sex subgroups in both ethnicities, except for T2D. Higher deciles of Hb concentration further corresponded to stronger odds of central obesity (Figure 2; Tables S12 and S13), hypertension (Tables S14 and S15), dyslipidemia (Tables S16 and S17), and gout (Tables S20 and S21) in both ethnic groups. However, the slightly higher MetS prevalence observed among the mostly anemic subjects in the lower Hb deciles corresponded to ORs that were non-significant. Our findings on diabetes were different from those of other MetS components (Figure 3; Tables S22 and S23). Interestingly, the highest or lower Hb deciles, as compared to the reference, were associated with stronger odds of T2D.

### 3.3. Metabolic Syndrome

The prevalence of MetS following the NCEP ATP III criteria was 25.7% among Taiwanese HC men and 18.9% among women at the time of assessment. MetS prevalence was slightly increased to 26.8% in men when using the Taiwan’s modified criteria. Higher prevalence rates among European Whites were observed in men at 49.0% and in women at 37.5%. Subjects in the highest Hb deciles who were classified as having MetS were at 40.7% and 34.4% among Taiwanese HC men and women, respectively, and 58.4% and 53.0% among European men and women, respectively.

At higher-than-normal range of Hb concentration, increasing deciles of Hb were significantly associated with MetS across all sex subgroups in both ethnicities (Figure 1). The highest Hb deciles resulted in up to three times greater odds of having MetS than the reference group, upon fully adjusting for age, age-squared, smoking status, drinking status, physical activity level, and highest educational attainment (and menopausal status in women) (Tables S10 and S11). Particularly among Taiwanese HC men and women, ORs for MetS were 3.17 (2.75–3.67, *p* < 0.0001) for Hb ≥ 16.5 g/dL and 3.11 (2.78–3.47, *p* < 0.0001) for Hb ≥ 14.5 g/dL, respectively. Abrupt increases in ORs between D8 and D10 in this group were evident (i.e., from 1.90 (1.65–2.20) at 15.7–16.0 g/dL to 2.36 (2.02–2.74) at 16.1–16.4 g/dL to 3.17 (2.75–3.67) when Hb was ≥16.5 g/dL in men). Among Europeans, much lower ORs were obtained despite a larger sample size and higher prevalence rates of MetS across Hb deciles: 1.89 (1.80–1.98, *p* < 0.0001) at Hb ≥16.24 g/dL in men and 2.35 (2.24–2.47, *p* < 0.0001) at Hb ≥ 14.68 g/dL in women. ORs were generally attenuated after fully adjusting for covariates.

Central obesity was more prevalent among Europeans than Taiwanese HC when following the WHO’s WC criteria and ethnic-specific cut-off points. Similar to MetS, more than half of the European subjects were with central obesity. It is also evident that among Europeans and Taiwanese HC in the highest deciles of Hb concentration, central obesity was highly prevalent.

Among the Taiwanese HC men and women, fully adjusted ORs from the highest Hb deciles were respectively 2.03 (1.77–2.33, *p* < 0.0001) and 1.72 (1.57–1.89, *p* < 0.0001) for central obesity (Table S12). On the other hand, ORs obtained from the highest deciles among European men and women were 2.21 (2.11–2.33, *p* < 0.0001) and 2.00 (1.91–2.11, *p* < 0.0001) (Table S13).

Monotonous risk elevations with increasing Hb deciles were observed in men and women for both ethnic groups. Among Europeans, starting from the lowest decile within normal Hb concentrations, highly significant positive associations with central obesity were noted.

### 3.4. Hypertension

Greater proportions of Europeans were found to be hypertensive than the Taiwanese HC, either based on blood pressure measurement, self-reported data, or hospital in-patient records. More than half of the European men (i.e., 52.8%) were hypertensive based on office blood pressure readings alone. Furthermore, the majority of European subjects in the 10th deciles were classified as hypertensive (i.e., 63.5% in men and 55.2% in women). On the other hand, hypertensive Taiwanese HC based on blood pressure readings were 24.5% among men and 13.0% among women, whereas only 18.4% of men and 10.6% of women had self-reported hypertension.

The fully adjusted ORs for hypertension from the highest Hb deciles in Taiwanese HC men and women were 1.95 (1.70–2.24, *p* < 0.0001) and 2.12 (1.89–2.39, *p* < 0.0001), respectively (Table S14). Among European men and women, ORs for hypertension were at 1.99 (1.89–2.10, *p* < 0.0001) and 2.22 (2.11–2.33, *p* < 0.0001), respectively (Table S15). Significant risk elevation starts from the 4th decile for both gender and ethnicities. However, ORs became non-significant if we further adjusted for BMI groups.

### 3.5. Dyslipidemia

Europeans with hypertriglyceridemia, hypercholesterolemia, high LDL-C, and low HDL-C levels based on blood lipid profile were all higher in prevalence rates as compared to the Taiwanese HC. Notably, among the Taiwanese HC, observed prevalence rates in the highest Hb deciles were as high as thrice of that in the reference deciles. Hypertriglyceridemia was 28.8% among the Taiwanese HC men whose Hb levels were ≥16.5 g/dL; whereas hypertriglyceridemia was only 10.9% among those whose Hb were 13.6–14.1 g/dL. Among Taiwanese HC women, the prevalence of high LDL-C was 19.8% when Hb was ≥14.5 g/dL, whereas only 7.7% were identified in the 12.2–12.5 g/dL Hb range.

The fully adjusted ORs for dyslipidemia (i.e., combination of either hypertriglyceridemia or low HDL-C levels) from the highest Hb deciles were 1.92 (1.68–2.20, *p* < 0.0001) among Taiwanese HC men and 1.50 (1.37–1.65, *p* < 0.0001) among women (Table S16); whereas ORs obtained in the highest deciles among European men and women were 1.51 (1.44–1.59, *p* < 0.0001) and 1.44 (1.37–1.51, *p* < 0.0001), respectively (Table S17). Specific to the Taiwanese HC group, we observed the lowest Hb deciles to be positively associated with dyslipidemia as compared to the reference: OR = 1.24 (1.07–1.42, *p* = 0.0034) at Hb ≤13.5 g/dL in men and OR = 1.35 (1.23–1.48, *p* < 0.0001) at Hb ≤ 11.5 g/dL in women.

We also observed higher risks at higher Hb deciles for hypercholesterolemia or raised levels of either total cholesterol or LDL-C (Tables S18 and S19). However, highly significant positive associations were only identified in the Taiwanese HC group, especially among women, whereas risk elevations started from the 4th decile in the European group. ORs from the highest Hb deciles were 1.92 (1.65–2.25) among Taiwanese HC men, 2.67 (2.37–3.02) among Taiwanese HC women, 1.23 (1.18–1.29) among European men, and 1.35 (1.29–1.42) among European women (*p* < 0.0001). As opposed to a combination of hypertriglyceridemia and low HDL-C levels, we did not find any significant positive association with the lowest Hb decile for elevated cholesterol levels.

### 3.6. Gout or Hyperuricemia

The percentages of Taiwanese HC men and women with hyperuricemia were almost twice of that in Europeans. In particular, 29.7% of Taiwanese HC men and 17.7% of European men had hyperuricemia whereas there were 14.1% of Taiwanese HC women and 9.2% of European women with hyperuricemia. Similar observations on self-reported gout were noted for both ethnic groups.

Among the Taiwanese HC men and women, fully adjusted ORs for gout from the highest Hb deciles were 1.33 (1.17–1.51, *p* < 0.0001) and 2.44 (2.15–2.78, *p* < 0.0001) (Table S20). A highly significant monotonous risk elevation was evident among the Taiwanese HC women. ORs obtained in the highest deciles among European men and women were 1.29 (1.22–1.37, *p* < 0.0001) and 1.84 (1.70–1.99, *p* < 0.0001), respectively (Table S21). Similar to dyslipidemia, significant positive associations with gout were also identified in the lowest Hb deciles: OR = 1.18 (1.03–1.35, *p* = 0.0181) at Hb ≤ 13.5 g/dL among Taiwanese HC men, OR = 1.11 (1.04–1.17, *p* = 0.0010) at Hb ≤ 13.79 g/dL among European men, and OR = 1.12 (1.03–1.23, *p* = 0.0113) at Hb ≤ 12.39 g/dL among European women.

### 3.7. Type 2 Diabetes

Type 2 diabetes based on HbA1c cut-off and self-reported data were observed to be higher among Taiwanese HC (i.e., 11.7% and 7.4%, respectively, in men) than Europeans (i.e., 4.4% and 6.5%, respectively, in men). However, the percentage of European men with diabetes increased (i.e., 17.4%) using hospital in-patient records. On the basis of FBG criteria, lower percentages of subjects with hyperglycemia were noted. There were even much lower counts of diabetic European subjects identified from an 11.1 mmol/L GLU threshold, which was set to exclude false positives from a non-fasting glucose reading in the UKB. Among Europeans, the mean GLU and HbA1c levels of men were in a down-and-up trend with increasing Hb concentration, although the upward trend was rather small. On the other hand, the mean GLU levels of women continuously decreased from the first to fourth decile, followed by a constant increase from the fifth to the last decile. Hyperglycemia prevalence across Hb deciles was not statistically significant in the Taiwanese HC men and European women subgroups.

A U-shaped curvilinear association between Hb and diabetes risk was evident (Tables S22 and S23). The ORs obtained for diabetes in the lowest or highest decile were as follows: Taiwanese HC men: OR = 1.71 (1.39–2.10, *p* < 0.0001) at ≤13.5 g/dL and OR = 1.64 (1.33–2.02, *p* < 0.0001) at ≥16.5 g/dL Hb; Taiwanese HC women: OR = 1.32 (1.12–1.56, *p* = 0.0010) at ≤11.5 g/dL and OR = 1.83 (1.59–2.10, *p* < 0.0001) at ≥14.5 g/dL Hb; European Whites men: OR = 2.10 (1.94–2.27, *p* < 0.0001) at ≤13.79 g/dL Hb; European Whites women: OR = 1.81 (1.63–2.01, *p* < 0.0001) at ≤12.39 g/dL and OR = 1.39 (1.26–1.54, *p* < 0.0001) at ≥14.68 g/dL Hb.

In both ethnic groups, the associations between increasing Hb and risks of individual metabolic components were found modest, as compared to the Hb-MetS associations. ORs were attenuated in the final regression models, wherein we additionally accounted for the presence of comorbidities. ORs were further attenuated upon adjusting for BMI groups in the models for hypertension, dyslipidemia, and gout, but the trends still remained (Tables S24.1–S24.8).

## 4. Discussion

Our sex-specific observational association studies determined and validated the relationship between iron excess, as depicted by elevated hemoglobin concentration levels, and the risks of metabolic syndrome and metabolic components (i.e., central obesity, hypertension, type 2 diabetes, dyslipidemia, and gout) in two major ethnic populations, the Han Chinese and Europeans. With the careful titration of the associations between Hb deciles and most MetS components, monotonous MetS risk elevations in both men and women were found to be evident with an increasing Hb concentration. We additionally discovered that the Hb-T2D association was different from other MetS components investigated, which showed more of an inverse association, but both the highest and lowest Hb deciles led to higher odds of T2D as compared to the middle deciles for both the Taiwanese HC and European cohorts. Few studies made comparison among all MetS components to provide insight to MetS etiology.

### 4.1. Elevated Hemoglobin Concentration Is Associated with Increased Risks of Metabolic Syndrome and Several Individual Metabolic Components

The positive association between Hb and prevalence of the MetS and metabolic components has been illustrated in a few previous studies [42,43,44]. Recently, in a cohort of Finnish men and women after a 20-year follow-up [45], higher Hb levels within the normal range were further shown to be associated with an increased incidence of MetS and key components, as well as higher risks of cardiovascular and total mortalities. A similar study conducted in a population of middle-aged and elderly Chinese also demonstrated high Hb levels as a potential predictor of the incidence of MetS and its components, including gout and non-alcoholic fatty liver disease, after years of follow-up [46]. Our present findings corroborated these previous results, while carefully depicting in two ethnicities the relationships for the whole Hb range.

The mechanisms underlying the reported associations between Hb levels and metabolic disorders (or markers) have been poorly understood and not clearly identified. Most evidence points to iron-mediated oxidative stress. Oxidative stress is defined as an imbalance between the overproduction of reactive oxygen species and insufficient antioxidant defenses, which may generally lead to negative health consequences due to free radical-mediated tissue damage [47]. Hb at higher end may also be regarded as a biomarker of the body’s inflammatory state [48], aside from being a putative indicator of oxidative stress. Both inflammation and oxidative stress are well-known associated factors in the development of obesity [48,49] and hypertension [50,51]. In dyslipidemia, one of the possible consequences of free radical damage is on lipid peroxidation, which causes the depletion of the cellular content of reduced glutathione, an important player for preventing cell damage [52]. Another proposed mechanism of action of high Hb levels is by increasing blood viscosity (i.e., hyperviscosity) following a prolonged state of oxidative stress that eventually decreases the blood flow throughout the body [53], raising the blood pressure systemically [54]. Elevated blood viscosity might also induce insulin resistance, which is believed to be the root cause of MetS [7]. Other suggested mechanisms linking Hb and MetS include changes in plasma volume, endothelial cell dysfunction, etc. [45].

Meanwhile, hyperuricemia has been recently proposed to be an additional cause of the MetS. Epidemiological studies have likewise shown a positive association between hyperuricemia or gout and MetS [55,56]. The relationship between hyperuricemia and iron accumulation has been illustrated with a strong correlation between serum uric acid and serum ferritin levels, the most commonly deployed indicator for determining iron status [57]. For this reason, elevated uric acid was further proposed as a potential indicator of iron overload [58]. It was hypothesized that the elevation in uric acid levels is a response to counter an increased oxidative stress in the body (i.e., compensatory rather than protective) as urate assumes the coordination sites of iron, leading to a decline in electron transport and subsequent oxidant generation [57]. Although the mechanism remains unclear, looking into the etiology and pathology of hyperuricemia is highly important for Asians due to the relatively higher prevalence rates seen in this group [59]. Based on our data alone, MetS and gout were both found to be more prevalent among the Taiwanese HC than Europeans. However, our findings further demonstrated that although a relationship has been detected, the association between elevated Hb and hyperuricemia or gout was not as great as compared to the other MetS components. This requires further studies.

Taken together, future studies are highly needed to confirm the role of elevated Hb concentration as a risk factor of MetS and its individual components. MetS and all components are considered as major risk factors for cardiovascular diseases and total mortality. In fact, MetS was reported to increase the mortality risk by 46% among individuals with MetS as compared to those without [60]. Therefore, if these associations are confirmed as causal, primary prevention and optimal treatment regimen employing measures to control iron overnutrition may eventually lead to decreased MetS incidence and morbidity and mortality.

### 4.2. U-Shaped Association between Hemoglobin Concentration and Type 2 Diabetes Risk

Epidemiological studies that determined the association between iron intake or body iron stores and T2D risk have produced conflicting results between sex and different ethnicities to date [61,62], probably due to failure to carefully titrate the association. However, few studies have demonstrated that the association of Hb concentration with cause-specific and total mortality rates is rather U-shaped and not linear [63,64], and that in general, both low- and high-end Hb levels within the normal variation have been considered beneficial for human health [65]. We are the first to carefully investigate the Hb–T2D relationship.

In diabetes, high levels and stores of iron in the body may result in a selective damage of the pancreatic beta-cells through excessive oxidative stress, which then leads to impaired insulin synthesis and release [66]. Hereditary or primary hemochromatosis frequently gives rise to T2D (i.e., referred to as secondary diabetes), and there are instances when diabetes is the only apparent manifestation of hemochromatosis in patients [67]. Primary hemochromatosis particularly affects the parenchymal cells; thus, producing significant tissue damage [68]. Secondary hemochromatosis or the acquired abnormalities of iron overload, on the other hand, initially affects the reticuloendothelial cells in many organ systems, but eventually will involve the parenchymal cells [69].

Lower-than-normal Hb concentrations were also implicated with the risk of T2D from our findings. In a study that utilized the Women’s Health Initiative data of ~160,000 postmenopausal women in order to examine the associations of Hb levels with cancer mortality, coronary heart disease mortality, and total mortality, it showed that both low and high deciles of Hb were positively associated with all outcomes, and that a low mean Hb level specifically demonstrated a robust, positive association even after three years of follow-up [63]. Another study similarly reported the association of both low and high Hb concentrations with cardiovascular and all-cause mortality after an eight-year follow-up [64]. T2D was not investigated in the above two studies. The lower end risk elevation may represent an end-stage anemia phenomenon, whereas the low Hb-T2D association due to the chronic impairment of insulin action and low-grade inflammation, among others, that eventually lead to many long-term micro- and macrovascular complications [70], suggests more of a causal relationship specifically for the etiology of diabetes mellitus, since other MetS components did not show this clear negative association.

Similar inverse association between Hb concentrations and HbA1c levels for both men and women, across the fasting glucose quintiles within the non-diabetic range, was observed in a cross-sectional analysis among ~87,000 adult non-anemic and non-diabetic Korean adults [71,72]. The inverse relationship between Hb and HbA1c has been also observed in other populations, and anemia has been considered as a confounder of HbA1c values [73]. Nonetheless, the underlying mechanisms and clinical relevance of the effect of low Hb concentrations, iron deficiency, and iron deficiency anemia on glucose homeostasis are yet to be clearly elucidated.

We surmise that the observed increased odds of T2D at lower-than-normal Hb concentrations among Taiwanese HC and Europeans in the present study may be caused by anemia that reflects poor nutrition status, which results in poor blood circulation, decreased oxidative capacity, and pancreatic islet malfunction [74]. The possibility of a reverse causation should also be explored. Type 2 diabetes leads to chronic kidney disease that in turn causes anemia, contributing to the poor prognosis for T2D patients [75]. Whether it is reasonable to see a very modest effect of low Hb concentrations on the pathology of other MetS components, but primarily on T2D alone, warrants additional investigation.

In the context of iron metabolism, hepcidin has been recently discovered and is now widely accepted as the key systemic iron-regulatory hormone [76]. The expression of hepcidin in the liver is stimulated by inflammation or iron overload. During inflammation or infection, when hepcidin level is high, erythrocyte production becomes restricted through the inhibition of intestinal iron absorption and macrophage iron recycling, which reduces Hb concentration and ultimately causes anemia (i.e., anemia of inflammation) [77]. Hepcidin is also thought to be homeostatically regulated by the iron requirements of erythroid precursors for hemoglobin synthesis, so that during active erythropoiesis (i.e., during anemia), hepcidin production is suppressed to make more iron available for hemoglobin synthesis [78].

Serum hepcidin level was similarly shown to be increased among subjects with the MetS at a population level, wherein a linear increase in hepcidin levels in both sex groups was observed with increasing number of MetS components [79]. Nonetheless, there is still scarcity of studies linking hepcidin and MetS to date, which is probably due to a number of challenges related to hepcidin assay development [80]. Although the hepcidin-MetS association has been established for the first time, more studies are needed for validation and further elucidation of the mechanisms relating hepcidin to other iron biomarkers, including Hb, in the complex pathophysiology of the MetS.

In the context of iron nutrition, neither a too low nor too high iron status is desirable for human health. We have consistently shown that higher-than-normal levels of Hb increased risks of MetS and majority of the metabolic components, whereas too less or too much may confer risk against T2D. Such findings may be used in the future to support the rectification of the estimated average requirements, recommended dietary allowances, and tolerable upper intake levels for iron among Taiwanese HC and European Whites. Additionally, the utilization of a binary, single cut-off point to define optimal iron status may need to be revisited as it tends to obscure the more subtle disease-Hb associations [63,81].

### 4.3. Strengths and Limitations of the Study

The present study has several strengths. First, as we try to answer what levels of iron nutrition is optimal in order to prevent or delay the onset and progression of MetS and its metabolic components, we are the first to titrate Hb concentrations by decile categorization (i.e., investigating the effects of small increments in Hb concentration) against most components of MetS altogether. Second, our analyses employed relatively large populations of the Taiwanese HC and European Whites, which allowed us to further conduct sex-specific titration analyses. The large sample size and consistent results between the two ethnic groups strengthen the findings of the present study. To our knowledge, we are the first to explore the association of Hb levels with the risk of MetS among the Han Chinese of Taiwan. Our ethnic-specific analyses addressed the apparent differences in terms of iron status between the East Asians and Caucasians [82,83]. Hemochromatosis is common among Caucasians or populations of European origin. Iron overload among Asian populations, on the contrary, is rare and not well understood. This is partly due to the relatively higher prevalence of hemoglobinopathies (i.e., thalassemia) in Asians such that there is a possible masking by the coexistence and high prevalence of iron deficiency and anemia [84]. Lastly, our sex-specific analyses addressed the known differences between genders in terms of Hb concentration and distribution and other indicators of iron metabolism [85]. Although the underpinning mechanism has not been clearly depicted, sex hormones and menstruation are thought to play roles that might cause such sex-differences. Men had a consistently higher prevalence of MetS and metabolic outcomes than women in both ethnic groups, except for central obesity, yet we found that for most outcomes, women were likely to have higher relative risks of developing metabolic disorders with higher levels of Hb concentration. Interestingly, for central obesity, the relative risks in men were contrarily higher than women. Further studies of other factors, be it genetic, environmental, or lifestyle, will elucidate the subtle ethnic- and sex-differences in iron nutrition and metabolism and on how it affects an individual’s susceptibility to the MetS and metabolic components. This will be needed in order to come up with more comprehensive population- and gender-specific recommendations and personalized nutrition interventions for iron-related disorders.

Our study has some limitations as well. Findings were derived from cross-sectional data and analyses, which means that causality cannot be inferred. Both the TWB and UKB were comprised datasets that are comprehensive enough to adjust for variables or parameters that may confound any Hb-outcome association observed. However, there is the possibility of unadjusted confounders. We were unable to consider the consumption of total dietary energy, saturated fat, and other nutrients, as well as the intake of iron supplements, iron absorption enhancers (i.e., vitamin C), or iron inhibitors (i.e., tannins) in the regression models because of some incomplete information collected during a personal interview in the TWB. We did not screen for vegetarians, subjects taking dietary supplements, or those who had consumed more red meat, which may have influenced the outcomes. Concurrent acute or chronic infections and autoimmune diseases of subjects and the confounding effect of inflammatory markers (i.e., C-reactive protein) may have been overlooked as well. Additionally, although our findings may have substantial impact on two major ethnicities, the East Asians and Caucasians, the generalizability of results to other groups (i.e., Africans, South Asians, etc.) is still not clear. Finally, as we anticipate the measurement and availability of other relevant iron status markers (i.e., serum hepcidin, serum ferritin, serum iron, transferrin saturation) in the TWB and UKB in the near future, exploration of the association of these markers with metabolic outcomes is highly warranted. Low and high Hb concentrations may be the results of other pathological conditions irrelevant to iron status. All these should be considered in future studies.

## 5. Conclusions

Hemoglobin reflects the major functional iron in the body and may serve as a surrogate of iron status. Elevated Hb concentration, a potential indicator of iron overnutrition, may play a role in the pathophysiology of MetS and its key components. It is highly warranted to further explore the observed protection against diabetes within normal Hb levels, the increased risks in both below normal and relatively higher levels, and the underlying mechanisms for such findings.

## Data Availability

Not applicable.

## Supplementary Materials

The following supporting information can be downloaded at: https://www.mdpi.com/article/10.3390/nu14194007/s1, File S1: List of variables and data field codes utilized in the UKB analyses, File S2: Disease definitions used in the UKB analyses, File S3: Prescription medication codes (data field 20003) in the UKB analyses, Table S1: Characteristics of subjects in the TWB across increasing deciles of Hb levels; Table S2: Characteristics of subjects in the UKB across increasing deciles of Hb levels; Table S3: Frequency distribution of metabolic outcomes among subjects in the TWB across increasing deciles of Hb levels; Table S4: Frequency distribution of metabolic outcomes among subjects in the UKB across increasing deciles of Hb levels; Table S5: Frequency distribution of self-reported metabolic conditions among subjects in the TWB across increasing deciles of Hb levels; Table S6: Frequency distribution of self-reported metabolic conditions among subjects in the UKB across increasing deciles of Hb levels; Table S7: Cases of metabolic disorders from hospital in-patient records of UKB subjects across increasing deciles of Hb levels; Table S8: Cross-tabulation of cases in the TWB based on self-reported metabolic disorders and high levels of biochemical markers; Table S9: Cross-tabulation of cases in the UKB based on high levels of anthropometric and biochemical markers, self-reported metabolic conditions, and hospital in-patient records of subjects; Table S10: Odds ratios of metabolic syndrome (NCEP ATP III criteria) across increasing deciles of Hb levels among Taiwanese Han Chinese; Table S10.1: Odds ratios of metabolic syndrome (Taiwan’s criteria) across increasing deciles of Hb levels among Taiwanese Han Chinese; Table S11: Odds ratios of metabolic syndrome (NCEP ATP III criteria) across increasing deciles of Hb levels among European Whites; Table S12: Odds ratios of central obesity across increasing deciles of Hb levels among Taiwanese Han Chinese; Table S13: Odds ratios of central obesity across increasing deciles of Hb levels among European Whites; Table S14: Odds ratios of hypertension across increasing deciles of Hb levels among Taiwanese Han Chinese; Table S15: Odds ratios of hypertension across increasing deciles of Hb levels among European Whites; Table S16: Odds ratios of dyslipidemia across increasing deciles of Hb levels among Taiwanese Han Chinese; Table S17: Odds ratios of dyslipidemia across increasing deciles of Hb levels among European Whites; Table S18: Odds ratios of hypercholesterolemia across increasing deciles of Hb levels among Taiwanese Han Chinese; Table S19: Odds ratios of hypercholesterolemia across increasing deciles of Hb levels among European Whites; Table S20: Odds ratios of gout across increasing deciles of hemoglobin levels among Taiwanese Han Chinese; Table S21: Odds ratios of gout across increasing deciles of hemoglobin levels among European Whites; Table S22: Odds ratios of diabetes across increasing deciles of hemoglobin levels among Taiwanese Han Chinese; Table S23: Odds ratios of diabetes across increasing deciles of hemoglobin levels among European Whites; Tables S24.1–S24.8: With BMI as additional covariate in the models.
