# Supplementary material for: Positive or U-Shaped Association of Elevated Hemoglobin Concentration Levels with Metabolic Syndrome and Metabolic Components: Findings from Taiwan Biobank and UK Biobank
Source: Nutrients. 2022 Sep 27;14(19):4007. doi: 10.3390/nu14194007 (PMC9572591; doi:10.3390/nu14194007)
Supplement: Supplementary file 1 [file nutrients-14-04007-s001.zip › Nutrients Supplementary Files_Timoteo,Chiang,Pan.pdf]

File S1: List of variables and data field codes utilized in the UK Biobank analyses

A. Anthropometry
Standing height (50); weight (21002); body mass index (21001); waist circumference (48); hip circumference (49); systolic blood pressure, automated reading (4080); diastolic blood pressure, automated reading (4079)

B. Hematological data
Haemoglobin concentration (30020)

C. Serum biochemistry markers
Cholesterol (30690); glucose (30740); glycated haemoglobin (30750); HDL cholesterol (30760); LDL cholesterol (30780); triglycerides (30870); urate (30880)

D. Socio-demographic data
Sex (31); genetic sex (22001); age when attended assessment centre (21003); ethnic background (21000); country of birth (1647); pregnant (3140); had menopause (2724); age at menopause (3581); qualifications (6138)

E. Health-related outcomes
Number of self-reported cancers (134); non-cancer illness code, self-reported (20002): 1192 - renal/kidney failure, 1193 - renal failure requiring dialysis, 1194 - renal failure not requiring dialysis, 1451 - hereditary/genetic haematological disorder, 1339 - sickle cell disease, 1340 - thalassemia, 1445 - clotting disorder/excessive bleeding, 1327 - low platelets/platelet disorder, 1328 - haemophilia, 1546 - essential thrombocytosis, 1439 - hiv/aids, 1440 - tuberculosis (tb), 1441 - malaria, 1443 - schistosomiasis/bilharzia, 1065 - hypertension, 1072 - essential hypertension, 1220 - diabetes, 1223 - type 2 diabetes, 1473 - high cholesterol, 1466 - gout; vascular/heart problems diagnosed by doctor (6150); diabetes diagnosed by doctor (2443); main ICD9 diagnoses (41203); secondary ICD9 diagnoses (41205); main ICD10 diagnoses (41202); secondary ICD10 diagnoses (41204)

F. Medications
Medication for cholesterol, blood pressure or diabetes (6177); medication for cholesterol, blood pressure, diabetes, or take exogenous hormones (6153); treatment/medication, prescription medications via verbal interview (20003)

G. Lifestyle
Smoking status (20116); current tobacco smoking (1239); alcohol drinker status (20117); alcohol intake frequency (1558); physical activity, IPAQ activity group (22032)

H. Others
Fasting time (74)

File S2: Disease definitions used in the UK Biobank analyses

Metabolic Outcome	ICD-9 Codes	ICD-10 Codes	Self-reported Fields
Obesity	278<br>2780, 2781, 2788	E66<br>E660, E661, E662, E668, E669	-
Hypertension	401-405<br>4010, 4011, 4019<br>4020, 4021, 4029<br>4030, 4031, 4039<br>4040, 4041, 4049<br>4050, 4051, 4059	I10-I13, I15, O10<br>I110, I119<br>I120, I129<br>I130, I131, I132, I139<br>I150, I151, I152, I158, I159<br>O100, O101, O102, O103, O104, O109	6150 (4), 6177 (2), 6153 (2), 20002 (1065, 1072)
Diabetes mellitus, type 2	250<br>2500, 25000, 25001, 25009<br>2501, 25010, 25011, 25019<br>2502, 25020, 25021, 25029<br>2503, 2504, 2505, 2506, 2507<br>2509, 25090, 25091, 25099	E11-E14<br>E110, E111, E112, E113, E114, E115, E116, E117, E118, E119<br>E120, E121, E122, E123, E124, E125, E126, E127, E128, E129<br>E130, E131, E132, E133, E134, E135, E136, E137, E138, E139<br>E140, E141, E142, E143, E144, E145, E146, E147, E148, E149	2443 (1), 6177 (3), 6153 (3), 20002 (1220, 1223)
Dyslipidemia	2720, 2721, 2722, 2723, 2724<br>27200, 27201, 27202, 27203, 27209<br>27240, 27248, 27249	E780, E781, E782, E783, E784, E785	6177 (1), 6153 (1), 20002 (1473)
Gout	274<br>2740, 2741, 2748, 2749	M10<br>M100, M101, M102, M103, M104, M109<br>M1000, M1001, M1002, M1003, M1004, M1005, M1006, M1007, M1008, M1009<br>M1010, M1011, M1012, M1013, M1014, M1015, M1016, M1017, M1018, M1019<br>M1020, M1021, M1022, M1023, M1024, M1025, M1026, M1027, M1028, M1029<br>M1030, M1031, M1032, M1033, M1034, M1035, M1036, M1037, M1038, M1039<br>M1040, M1041, M1042, M1043, M1044, M1045, M1046, M1047, M1048, M1049<br>M1090, M1091, M1092, M1093, M1094, M1095, M1096, M1097, M1098, M1099	20002 (1466)

File S3: Prescription medication codes (data field 20003) in the UK Biobank analyses

A. Anti-hypertensive drugs
1140888578 - antihypertensive, 1140860470 - methyldopa, 1140860558 - metalpha 250mg tablet, 1140860478 - aldomet 125mg tablet, 1140910606 - alpha methyldopa, 1141164148 - imidapril hydrochloride, 1141164154 - tanatril 5mg tablet, 1140860882 - cilazapril, 1140860892 - vascace 250micrograms tablet, 1140860728 - quinapril, 1140881706 - accupro 5mg tablet, 1140860736 - accuretic tablet, 1140860750 - captopril, 1140860758 - capoten 12.5mg tablet, 1140860764 - captopril+hydrochlorothiazide 25mg/12.5mg tablet, 1141150560 - kaplon 12.5mg tablet, 1140881714 - capozide tablet, 1140851692 - capozide 50mg tablets x28, 1140888552 - enalapril, 1140860776 - innovace 2.5mg tablet, 140860784 - innozide tablet, 1140860790 - enalapril maleate+hydrochlorothiazide 20mg/12.5mg tablet, 1140881712 - renitec 5mg tablet, 1140860696 - lisinopril, 1140864952 - lisinopril+hydrochlorothiazide 10mg/12.5mg tablet, 1140860706 - carace 2.5mg tablet, 1140864910 - carace 10 plus tablet, 1140860714 - zestril 2.5mg tablet, 1140864618 - zestoretic 10 tablet, 1140888560 – perindopril, 1140860802 - coversyl 2mg tablet, 1141180592 - perindopril+indapamide, 1140860806 - ramipril, 1141165470 - felodipine+ramipril, 1141165476 - triapin mite 2.5mg/2.5mg tablet, 1141188408 - tritace 1.25mg tablet, 1141199940 - lopace 2.5mg capsule, 1140888556 - fosinopril, 1140860878 - staril 10mg tablet, 1140864176 - monozide 10 tablet, 1140860904 - trandolapril, 1140860912 - gopten 500micrograms capsule, 1140923712 - moexipril, 1141145658 - angiotensin ii receptor antagonist, 1140866758 - vasaten 50mg tablet, 1141171336 - eprosartan, 1141171344 - teveten 300mg tablet, 1141156836 - candesartan cilexetil, 1141156846 - amias 2mg tablet, 1141152998 - irbesartan, 1141153006 - aprovel 75mg tablet, 1141172682 - irbesartan+hydrochlorothiazide 150mg/12.5mg tablet, 1141172686 - coaprovel 150mg/12.5mg tablet, 1140916356 - losartan, 1140916362 - cozaar half strength 25mg tablet, 1141151018 - cozaar-comp 50mg/12.5mg tablet, 1141179974 - cozaar 25mg tablet, 1141151016 - losartan potassium+hydrochlorothiazide 50mg/12.5mg tablet, 1141145660 - valsartan, 1141145668 - diovan 40mg capsule, 1141201038 - valsartan+hydrochlorothiazide 80mg/12.5mg tablet, 1141201040 - co-diovan 80mg/12.5mg tablet, 1141193282 - olmesartan, 1141193346 - olmetec 10mg tablet, 1141166006 - telmisartan, 1141187790 - micardisplus 40mg/12.5mg tablet, 1141172492 - micardis 20mg tablet, 1141187788 - telmisartan+hydrochlorothiazide 40mg/12.5mg tablet, 1140872568 - nimodipine, 1140872472 - nimotop 30mg tablet, 1141153026 - lercanidipine, 1141153032 - zanidip 10mg tablet, 1140879802 - amlodipine, 1140861202 - istin 5mg tablet, 1141200400 - amlostin 5mg tablet, 1140888646 - felodipine, 1141190160 - vascalpha 5mg m/r tablet, 1141187094 - cabren 2.5mg m/r tablet, 1141199858 - cardioplen xl 5mg m/r tablet, 1141188836 - felendil xl 5mg m/r tablet, 1141188576 - felogen xl 5mg m/r tablet, 1141188152 - felotens xl 5mg m/r tablet, 1141188920 - keloc sr 5mg m/r tablet, 1141200782 - neofel xl 5mg m/r tablet, 1140868036 - parmid 10mg tablet, 1141201814 - parmid xl 5mg m/r tablet, 1140928212 - plendil 2.5mg m/r tablet, 1141150500 - slofedipine 20mg m/r tablet, 1140861088 - nifedipine, 1140860358 - tenif capsule, 1140926188 - unipine xl 30mg m/r tablet, 1140923572 - adipine mr 10 m/r tablet, 1140861090 - adalat 5mg capsule, 1140881702 - adalat 10mg capsule, 1140861106 - calcilat 10mg capsule, 1140861120 - coracten sr 10mg m/r capsule, 1140860426 - atenolol+nifedipine 50mg/20mg m/r capsule, 1140860356 - beta-adalat capsule, 1141173766 - calchan mr 10mg m/r tablet, 1141145870 - fortipine la40 m/r tablet, 1141157140 - nifedipress mr 10 m/r tablet, 1141150538 - nifedotard 20mr m/r tablet, 1141169730 - nifopress retard 20mg m/r tablet, 1140927940 - tensipine mr 10 m/r tablet, 1140861276 - lacidipine, 1140861282 - motens 2mg tablet, 1140879806 - diltiazem, 1140861128 - tildiem 60mg m/r tablet, 1140861136 - angiozem 60mg m/r tablet, 1140861138 - adizem-60 m/r tablet, 1140926780 - adizem-xl plus m/r capsule, 1140861166 - dilzem sr 60mg long acting m/r capsule, 1140926778 - diltiazem hcl+hydrochlorothiazide 150mg/12.5mg m/r capsule, 1140917428 - angitil sr 90 m/r capsule, 1141175224 - bi-carzem sr 60mg m/r capsule, 1141157136 - dilcardia sr 60mg m/r capsule, 1140911698 - slozem 120mg m/r capsule, 1141151474 - viazem xl 120mg m/r capsule,

1141174684 - zemret 180 xl m/r capsule, 1141167832 - zemtard 120 xl m/r capsule, 1140888510 – verapamil, 1141169710 - vertab sr 240 m/r tablet, 1141150926 - verapress mr 240 m/r tablet, 1141187774 - vera-til sr 120mg m/r tablet, 1140881692 - univer 120mg m/r capsule, 1141153328 - trandolapril+verapamil hydrochloride, 1141153316 - tarka 2mg/180mg m/r capsule, 1140866466 - securon 40mg tablet, 1140866460 - half securon sr 120mg m/r tablet, 1140866554 - cordilox 40mg tablet, 1140851730 - calcicard 60mg tablet, 1140860338 - viskaldix tablet, 1140879810 - nicardipine, 1140861176 - cardene 20mg capsule, 1140861190 - isradipine, 1140881894 - diuretic, 1140866092 - metolazone, 1140866094 - metenix-5 tablet, 1140866090 - methyclothiazide, 1140909708 - furosemide, 1140866116 - frusemide, 1140866412 - lasilactone capsule, 1140866506 - frusemide+potassium 20mg/10mmol m/r tablet, 1141195258 - furosemide+potassium 20mg/10mmol m/r tablet, 1140866332 - triamterene+frusemide 50mg/40mg tablet, 1140866194 - frusid 40mg tablet, 1140851414 - frumax 40mg tablet, 1140866406 - frumil tablet, 1140866408 - frusene tablet, 1140866418 - fru-co tablet, 1140866248 - lasix 20mg tablet, 1140888496 - torasemide, 1140864874 - torem 2.5mg tablet, 1140866122 - bendrofluazide, 1140910442 - bzt – bendrofluazide, 1140860340 - timolol maleate+bendrofluazide 10mg/2.5mg tablet, 1140866450 - bendrofluazide+potassium 2.5mg/7.7mmol m/r tablet, 1141146126 - atenolol+bendrofluazide, 1141194794 - bendroflumethiazide, 1141194800 - bendroflumethiazide+potassium 2.5mg/7.7mmol m/r tablet, 1140860312 - nadolol+bendroflumethiazide 40mg/5mg tablet, 1141194808 - timolol maleate+bendroflumethiazide 10mg/2.5mg tablet, 1141194810 - atenolol+bendroflumethiazide, 1140866136 - neo-naclex 5mg tablet, 1140866446 - neo-naclex k m/r tablet, 1140866162 - hydrochlorothiazide, 1140888686 - hydralazine, 1140866156 - cyclopenthiazide, 1140866158 - navidrex 500mcg tablet, 1140909706 - chlortalidone, 1140866144 - chlorthalidone, 1140866410 - kalspare tablet, 1140866146 - hygroton 50mg tablet, 1141180778 - atenolol+chlortalidone, 1140851484 - paritane 20mg tablet, 1140866108 - xipamide, 1140866110 - diurexan 20mg tablet, 1140866078 - indapamide, 1141180592 - perindopril+indapamide, 1141180598 - coversyl plus 4mg/1.25mg tablet, 1141201244 - eplerenone, 1141201250 - inspra 25mg tablet, 1141146378 - natrilix sr 1.5mg m/r tablet, 1140888922 - nindaxa 2.5mg tablet, 1140888512 - amiloride, 1140866352 - navispare tablet, 1140923276 - co-amilozide, 1140866422 - amiloride hcl+cyclopenthiazide 2.5mg/250micrograms tablet, 1140866426 - amiloride hydrochloride+bumetanide 5mg/1mg tablet, 1140866416 - moduret 25 tablet, 1140866420 - moduretic tablet, 1140927174 - amilamont 5mg/ml s/f oral solution, 1140866226 - berkamil 5mg tablet, 1140866280 - bumetanide, 1140866448 - bumetanide+potassium 500micrograms/7.7mmol m/r tablet, 1140866282 - burinex 1mg tablet, 1140866438 - burinex k m/r tablet, 1140866356 - burinex a tablet, 1140866388 - triamterene, 1140866328 - triam-co tablet, 1140866402 - dyazide tablet, 1140866236 - spironolactone, 1140866244 - aldactone 25mg tablet, 1140866312 - spiroctan 25mg tablet, 1140866318 - spirolone 25mg tablet, 1140923282 - co-flumactone, 1140866396 - aldactide 25 tablet, 1140916342 - beta-blocker, 1140866692 - beta-adrenoceptor blocking drug, 1140879854 - sotalol, 1140860304 - beta-cardone 40mg tablet, 1140860362 - sotacor 80mg tablet, 1140860332 - sotalol hydrochloride+hydrochlorothiazide 80mg/12.5mg tablet, 1140860292 - pindolol, 1140860294 - visken 5mg tablet, 1140910614 - prindolol, 1141164276 - nebivolol, 1141164280 - nebilet 5mg tablet, 1140860192 - nadolol, 1141194804 - nadolol+bendroflumethiazide 40mg/5mg tablet, 1140860194 - corgard 40mg tablet, 1140875808 - metipranolol, 1140860278 - mepranix 50mg tablet, 1140909368 - carvedilol, 1141168498 - eucardic 3.125 tablet, 1140866724 - acebutolol, 1140866726 - sectral 100mg capsule, 1140866738 - atenolol, 1140866756 - tenormin 25 tablet, 1140922930 - atenix 25mg tablet, 1140860348 - atenixco 50mg/12.5mg tablet, 1141146124 - atenolol+chlorthalidone, 1140923336 - co-tenidone, 1140860324 - tenoret 50 tablet, 1140860328 - tenoretic tablet, 1141146128 - atenolol+co-amilozide, 1140879760 - bisoprolol, 1140860434 - monocor 5mg tablet, 1140860492 - emcor 10mg tablet, 1140864950 - bisoprolol fumarate+hydrochlorothiazide 10mg/6.25mg tablet, 1141171152 - cardicor 1.25mg tablet, 1141182904 - soloc 5mg tablet, 1140879762 - celiprolol, 1140860498 - celectol 200mg tablet, 1140879818 - metoprolol, 1140860308 -

metoprolol tartrate+chlorthalidone 100mg/12.5mg tablet, 1140860404 - metoprolol tartrate+hydrochlorothiazide 100mg/12.5mg tablet, 1140860266 - betaloc 50mg tablet, 1140860274 - lopresor 50mg tablet, 1141182968 - tensomex 100mg tablet, 1140879824 - labetalol, 1140860250 - trandate 50mg tablet, 1140879830 - oxprenolol, 1140860334 - trasidrex tablet, 1140860220 - slow-trasicor 160mg m/r tablet, 1140860222 - trasicor 20mg tablet, 1140879842 - propranolol, 1141156754 - half propatard la 80mg m/r capsule, 1140866704 - angilol 10mg tablet, 1140860418 - propranolol hydrochloride+bendrofluazide 80mg/2.5mg capsule, 1140866712 - cardinol 10mg tablet, 1140866764 - apsolol 10mg tablet, 1140866804 - inderal 10mg tablet, 1140866800 - half-inderal la 80mg m/r capsule, 1140851556 - bedranol 10mg tablet, 1140866782 - beta-prograne 160mg m/r capsule, 1140866802 - half beta-prograne 80mg m/r capsule, 1141152076 - half propanix la 80mg m/r capsule, 1140866766 - propanix 10mg tablet, 1141187048 - rapranol sr 80mg m/r capsule, 1140860336 - timolol maleate+co-amilozide 10mg/2.5mg/25mg tablet, 1140879778 - doxazosin, 1140860690 - cardura 1mg tablet, 1141194372 - doxadura 1mg tablet

B. Anti-hyperglycemic drugs
1140883066 - insulin product, 1140884600 - metformin, 1141189090 - rosiglitazone 1mg / metformin 500mg tablet, 1141189094 - avandamet 1mg / 500mg tablet, 1140874686 - glucophage 500mg tablet, 1140874744 - gliclazide, 1140874746 - diamicron 80mg tablet, 1140874646 - glipizide, 1141157284 - glipizide product, 1140874652 - minodiab 2.5mg tablet, 1140874658 - gliquidone, 1140874664 - tolazamide, 1141152590 - glimepiride, 1141156984 - amaryl 1mg tablet, 1140874718 - glibenclamide, 1140874674 - tolbutamide, 1140874706 - chlorpropamide, 1140874716 - glymese 250mg tablet, 1141168660 - repaglinide, 1141168668 - novonorm 0.5mg tablet, 1141173882 - nateglinide, 1141173786 - starlix 60mg tablet, 1141171646 - pioglitazone, 1141171652 - actos 15mg tablet, 1141177600 - rosiglitazone, 1141177606 - avandia 4mg tablet, 1140874826 - glucagon product, 1140868902 - acarbose, 1140868908 - glucobay 50mg tablet

C. Triglyceride-lowering drugs
1140861924 - bezafibrate, 1141157260 - bezafibrate product, 1140861926 - bezalip 200mg tablet, 1140861928 - bezalip-mono 400mg m/r tablet, 1141201306 - fibrazate xl 400mg m/r tablet, 1140861954 - fenofibrate, 1140862026 - ciprofibrate, 1140862028 - modalim 100mg tablet, 1140861944 - clofibrate, 1140861856 - gemfibrozil (lopid), 1141157262 - gemfibrozil product, 1140861858 - lopid 300 capsule, 1140910670 - niacin, 1141188546 - niaspan 500mg m/r tablet, 1140861868 - nicotinic acid, 1193 - omega-3/fish oil supplement, 1141181868 - omacor 1g capsule

D. Cholesterol-lowering drugs
1140861958 - simvastatin, 1140881748 - zocor 10mg tablet, 1141200040 - zocor heart-pro 10mg tablet, 1141188146 - simvador 10mg tablet, 1140888594 - fluvastatin, 1140864592 - lescol 20mg capsule, 1140888648 - pravastatin, 1140861970 - lipostat 10mg tablet, 1141146234 - atorvastatin, 1141146138 - lipitor 10mg tablet, 1141192410 - rosuvastatin, 1141192414 - crestor 10mg tablet, 1140910632 - eptastatin, 1140910654 - velastatin, 1141192736 - ezetimibe, 1141192740 - ezetrol 10mg tablet, 1140861936 - questran 4g/sachet powder, 1140865576 - cholestyramine, 1141157416 - cholestyramine product, 1140861942 - cholestyramine+aspartame 4g/sachet powder, 1140909780 - colestyramine, 1141180734 - colestyramine product, 1141180722 - colestyramine+aspartame 4g/sachet powder, 1140888590 - colestipol, 1140861848 - colestid 5g/sachet granules

E. Anti-hyperuricemic drugs
1140875408 - allopurinol, 1140875476 - zyloric 100mg tablet, 1140875478 - zyloric 300mg tablet, 1140875496 - sulphinpyrazone, 1140909890 - sulfinpyrazone, 1140875496 - sulphinpyrazone, 1140875498 - anturan 100mg tablet, 1140875490 - probenecid, 1140875486 - colchicine
