# Supplementary material for: Positive or U-Shaped Association of Elevated Hemoglobin Concentration Levels with Metabolic Syndrome and Metabolic Components: Findings from Taiwan Biobank and UK Biobank
Source: Nutrients. 2022 Sep 27;14(19):4007. doi: 10.3390/nu14194007 (PMC9572591; doi:10.3390/nu14194007)
Supplement: Supplementary file 1 [file nutrients-14-04007-s001.zip › Nutrients Supplementary Tables_Timoteo,Chiang,Pan.pdf]

Table S1: Characteristics of subjects in the Taiwan Biobank (*N*=67,237) across increasing deciles of Hb levels

Variable		D1	D2	D3	D4	D5	D6	D7	D8	D9	D10	*P*-value for Trend
**Men, *n* (%)**	20,670 (30.7)	1,997 (9.7)	2,110 (10.2)	2,250 (10.9)	2,024 (9.8)	2,269 (11.0)	1,535 (7.4)	2,192 (10.6)	2,433 (11.8)	1,761 (8.5)	2,099 (10.2)	
Decile range, g/dL		9.0-13.5	13.6-14.1	14.2-14.5	14.6-14.8	14.9-15.1	15.2-15.3	15.4-15.6	15.7-16.0	16.1-16.4	16.5-20.3	
Hemoglobin, g/dL	15.1 ± 1.2	12.7 ± 0.8	13.9 ± 0.2	14.4 ± 0.1	14.7 ± 0.1	15.0 ± 0.1	15.2 ± 0.1	15.5 ± 0.1	15.8 ± 0.1	16.2 ± 0.1	17.0 ± 0.5	<0.0001
Age, y	50.8 ± 11.3	54.6 ± 11.0	53.0 ± 11.2	51.9 ± 11.3	51.2 ± 11.1	51.0 ± 11.4	50.0 ± 11.1	49.5 ± 11.3	49.4 ± 11.2	48.2 ± 11.0	48.8 ± 10.9	<0.0001
30-39	4,493 (21.7)	278 (13.9)	358 (17.0)	438 (19.5)	402 (19.9)	503 (22.2)	357 (23.3)	548 (25.0)	602 (24.7)	482 (27.4)	525 (25.0)	
40-49	4,510 (21.8)	320 (16.0)	404 (19.2)	441 (19.6)	431 (21.3)	454 (20.0)	352 (22.9)	521 (23.8)	581 (23.9)	444 (25.2)	562 (26.8)	
50-59	5,834 (28.2)	548 (27.4)	565 (26.8)	659 (29.3)	595 (29.4)	660 (29.1)	449 (29.3)	592 (27.0)	689 (28.3)	490 (27.8)	587 (28.0)	
60-70	5,833 (28.3)	851 (42.6)	783 (37.1)	712 (31.6)	596 (29.5)	652 (28.7)	377 (24.6)	531 (24.2)	561 (23.1)	345 (19.6)	425 (20.3)	
Weight, kg	73.1 ± 11.8	69.3 ± 11.1	70.6 ± 11.1	71.5 ± 11.1	72.1 ± 11.3	72.6 ± 11.3	73.6 ± 11.6	74.0 ± 11.4	74.5 ± 12.1	75.6 ± 12.1	77.1 ± 12.6	<0.0001
Body mass index, kg/m^2	25.4 ± 3.6	24.6 ± 3.5	24.7 ± 3.4	24.9 ± 3.3	25.0 ± 3.4	25.3 ± 3.4	25.5 ± 3.5	25.6 ± 3.4	25.8 ± 3.6	26.1 ± 3.6	26.7 ± 3.8	<0.0001
Waist circumference, cm	88.2 ± 9.4	86.2 ± 9.6	86.4 ± 9.2	87.1 ± 9.2	87.1 ± 9.3	87.8 ± 9.0	88.5 ± 9.0	88.8 ± 9.1	89.1 ± 9.5	90.0 ± 9.2	91.6 ± 9.7	<0.0001
Waist-hip ratio	0.904 ± 0.06	0.900 ± 0.06	0.896 ± 0.06	0.898 ± 0.06	0.897 ± 0.06	0.902 ± 0.06	0.904 ± 0.05	0.905 ± 0.06	0.907 ± 0.06	0.912 ± 0.05	0.923 ± 0.05	<0.0001
Systolic blood pressure, mm Hg	126.9 ± 16.8	126.6 ± 16.9	125.5 ± 16.7	125.6 ± 16.5	126.4 ± 17.1	126.8 ± 16.7	126.7 ± 16.4	126.8 ± 16.3	126.9 ± 16.3	128.2 ± 17.2	130.2 ± 17.5	<0.0001
Diastolic blood pressure, mm Hg	78.6 ± 10.6	75.4 ± 10.3	76.0 ± 10.1	76.8 ± 10.0	77.7 ± 10.3	78.3 ± 10.3	78.8 ± 10.2	79.3 ± 10.2	79.7 ± 10.4	81.0 ± 10.9	82.8 ± 11.2	<0.0001
Fasting blood glucose, mg/dL	99.7 ± 24.1	99.8 ± 24.7	98.4 ± 22.8	99.0 ± 21.5	98.3 ± 20.7	98.4 ± 20.6	98.5 ± 21.4	98.9 ± 21.2	99.6 ± 23.4	100.8 ± 26.7	105.2 ± 34.1	<0.0001
Glycated hemoglobin, %	5.91 ± 0.94	5.94 ± 0.96	5.90 ± 0.87	5.89 ± 0.85	5.87 ± 0.86	5.87 ± 0.83	5.86 ± 0.83	5.86 ± 0.83	5.89 ± 0.89	5.93 ± 1.02	6.10 ± 1.30	0.9639
Triglycerides, mg/dL	139.1 ± 109.2	115.8 ± 88.6	116.9 ± 77.8	124.4 ± 99.5	127.7 ± 88.0	131.2 ± 86.0	144.9 ± 123.0	142.0 ± 115.2	150.5 ± 114.0	160.0 ± 128.5	180.8 ± 142.4	<0.0001
Total cholesterol, mg/dL	191.5 ± 34.9	177.2 ± 34.5	185.7 ± 32.7	188.5 ± 33.1	189.9 ± 35.3	192.1 ± 34.2	195.0 ± 35.1	194.5 ± 34.6	195.6 ± 34.0	197.3 ± 34.3	199.9 ± 36.0	<0.0001
LDL-cholesterol, mg/dL	120.7 ± 31.4	108.1 ± 29.5	115.7 ± 29.1	117.5 ± 29.8	119.5 ± 31.9	121.6 ± 30.9	123.5 ± 31.4	124.0 ± 31.5	124.3 ± 31.0	126.5 ± 31.2	127.3 ± 32.6	<0.0001
HDL-cholesterol, mg/dL	47.9 ± 11.1	48.3 ± 12.2	49.3 ± 11.6	49.4 ± 11.9	48.9 ± 11.1	48.4 ± 11.0	47.7 ± 10.9	47.7 ± 10.8	47.2 ± 10.6	46.2 ± 10.4	45.3 ± 10.0	<0.0001
Uric acid, mg/dL	6.42 ± 1.37	6.29 ± 1.50	6.28 ± 1.33	6.24 ± 1.34	6.32 ± 1.33	6.42 ± 1.34	6.46 ± 1.34	6.47 ± 1.35	6.51 ± 1.31	6.57 ± 1.38	6.68 ± 1.42	<0.0001
Smoking status												<0.0001
Never smoked	11,112 (53.8)	1,097 (54.9)	1,198 (56.8)	1,232 (54.8)	1,123 (55.5)	1,263 (55.7)	834 (54.4)	1,221 (55.8)	1,303 (53.6)	911 (51.8)	925 (44.2)	
Stopped smoked	5,093 (24.6)	561 (28.1)	571 (27.1)	586 (26.1)	499 (24.7)	575 (25.3)	375 (24.5)	500 (22.8)	571 (23.5)	382 (21.7)	472 (22.5)	
Occasionally smoking	134 (0.7)	14 (0.70)	17 (0.81)	17 (0.76)	16 (0.79)	19 (0.84)	10 (0.65)	9 (0.41)	13 (0.53)	11 (0.63)	8 (0.38)	
Currently smoking	4,331 (21.0)	325 (16.3)	323 (15.3)	414 (18.4)	385 (19.0)	412 (18.2)	314 (20.5)	460 (21.0)	544 (22.4)	456 (25.9)	690 (32.9)	

Alcohol drinking status												<0.0001
Stopped drinking	1,189 (5.8)	152 (7.6)	117 (5.6)	125 (5.6)	119 (5.9)	127 (5.6)	87 (5.7)	118 (5.4)	133 (5.5)	89 (5.1)	122 (5.8)	
Occasionally drinking	16,751 (81.0)	1,633 (81.8)	1,741 (82.6)	1,857 (82.6)	1,656 (81.9)	1,857 (81.8)	1,241 (81.0)	1,769 (80.8)	1,982 (81.5)	1,396 (79.3)	1,612 (77.0)	
Currently drinking	2,730 (13.2)	212 (10.6)	251 (11.9)	267 (11.9)	248 (12.3)	285 (12.6)	205 (13.4)	303 (13.8)	316 (13.0)	275 (15.6)	361 (17.2)	
Physical activity												<0.0001
With regular exercise	8,949 (43.3)	1,011 (50.7)	1,058 (50.2)	1,057 (47.0)	902 (44.6)	982 (43.3)	622 (40.7)	865 (39.5)	989 (40.7)	690 (39.2)	769 (36.7)	
Highest educational attainment												<0.0001
No formal teaching or up to primary education	691 (3.3)	124 (6.2)	87 (4.1)	84 (3.7)	72 (3.6)	61 (2.7)	42 (2.7)	56 (2.6)	69 (2.8)	44 (2.5)	55 (2.6)	
Secondary education to high school	6,546 (31.7)	712 (35.3)	622 (29.5)	690 (30.7)	619 (30.6)	721 (31.8)	482 (31.4)	641 (29.3)	781 (32.1)	553 (31.4)	727 (34.7)	
University to post-graduate studies	13,430 (65.0)	1,181 (58.6)	1,399 (66.4)	1,475 (65.6)	1,331 (65.8)	1,486 (65.5)	1,009 (65.8)	1,493 (68.2)	1,581 (65.0)	1,163 (66.1)	1,313 (62.7)	
**Women, *n* (%)**	46,567 (69.3)	4,801 (10.3)	4,043 (8.7)	4,464 (9.6)	4,558 (9.8)	5,211 (11.2)	5,698 (12.2)	3,368 (7.2)	4,650 (10.0)	5,534 (11.9)	4,240 (9.1)	
Decile range, g/dL		6.6-11.5	11.6-12.1	12.2-12.5	12.6-12.8	12.9-13.1	13.2-13.4	13.5-13.6	13.7-13.9	14.0-14.4	14.5-18.7	
Hemoglobin, g/dL	13.0 ± 1.3	10.4 ± 1.1	11.9 ± 0.2	12.4 ± 0.1	12.7 ± 0.1	13.0 ± 0.1	13.3 ± 0.1	13.6 ± 0.1	13.8 ± 0.1	14.2 ± 0.1	14.9 ± 0.4	<0.0001
Age, y	50.3 ± 10.5	45.7 ± 9.4	48.3 ± 10.6	49.4 ± 10.6	50.0 ± 10.8	50.5 ± 10.7	51.0 ± 10.4	51.1 ± 10.5	51.9 ± 10.2	52.1 ± 10.1	53.1 ± 9.5	<0.0001
30-39	9,376 (20.1)	1,379 (28.7)	1,049 (26.0)	1,023 (22.9)	1,010 (22.2)	1,094 (21.0)	1,046 (18.4)	630 (18.7)	771 (16.6)	859 (15.5)	515 (12.1)	
40-49	10,750 (23.1)	1,941 (40.4)	1,109 (27.4)	1,098 (24.6)	1,015 (22.3)	1,064 (20.4)	1,207 (21.2)	694 (20.6)	858 (18.5)	1,048 (18.9)	716 (16.9)	
50-59	15,930 (34.2)	945 (19.7)	1,143 (28.3)	1,414 (31.7)	1,490 (32.7)	1,816 (34.9)	2,106 (37.0)	1,207 (35.8)	1,798 (38.7)	2,170 (39.2)	1,842 (43.4)	
60-70	10,511 (22.6)	536 (11.2)	742 (18.4)	929 (20.8)	1,043 (22.9)	1,237 (23.7)	1,339 (23.5)	837 (24.9)	1,223 (26.3)	1,457 (26.3)	1,168 (27.5)	
Had menopause	23,907 (51.3)	1,089 (22.5)	1,640 (40.6)	2,114 (47.4)	2,300 (50.5)	2,799 (53.7)	3,168 (55.6)	1,874 (55.7)	2,781 (59.8)	3,385 (61.2)	2,757 (65.0)	<0.0001
Age at menopause	49.4 ± 5.0	49.3 ± 4.7	49.4 ± 5.1	49.4 ± 5.0	49.3 ± 5.0	49.3 ± 5.1	49.4 ± 4.8	49.3 ± 5.2	49.5 ± 5.0	49.6 ± 4.9	49.5 ± 5.0	0.0929
Weight, kg	58.6 ± 9.9	58.3 ± 9.9	57.5 ± 9.6	57.4 ± 9.4	57.6 ± 9.5	57.6 ± 9.3	58.4 ± 9.8	58.9 ± 9.9	58.9 ± 10.0	59.8 ± 10.1	61.2 ± 10.7	<0.0001
Body mass index, kg/m^2	23.6 ± 3.8	23.2 ± 3.7	23.1 ± 3.7	23.1 ± 3.5	23.2 ± 3.5	23.3 ± 3.5	23.6 ± 3.7	23.8 ± 3.7	23.9 ± 3.8	24.2 ± 3.9	24.9 ± 4.1	<0.0001
Waist circumference, cm	80.9 ± 9.7	79.5 ± 9.5	79.4 ± 9.6	79.4 ± 9.3	79.7 ± 9.3	80.1 ± 9.2	80.9 ± 9.5	81.4 ± 9.5	81.5 ± 9.8	82.5 ± 9.9	84.3 ± 10.3	<0.0001
Waist-hip ratio	0.849 ± 0.07	0.837 ± 0.07	0.838 ± 0.07	0.839 ± 0.07	0.842 ± 0.07	0.846 ± 0.07	0.849 ± 0.07	0.853 ± 0.07	0.855 ± 0.07	0.860 ± 0.07	0.872 ± 0.07	<0.0001
Systolic blood pressure, mm Hg	117.6 ± 18.1	113.2 ± 16.4	114.0 ± 16.9	114.4 ± 16.7	115.5 ± 17.7	116.3 ± 17.7	117.7 ± 17.6	118.5 ± 18.3	120.0 ± 18.3	121.4 ± 18.3	125.0 ± 19.2	<0.0001

Diastolic blood pressure, mm Hg	71.1 ± 10.2	68.3 ± 9.8	68.6 ± 9.6	68.8 ± 9.3	69.5 ± 9.8	70.1 ± 9.8	71.1 ± 9.8	71.8 ± 10.2	72.6 ± 10.0	73.7 ± 10.2	76.4 ± 10.7	<0.0001
Fasting blood glucose, mg/dL	94.2 ± 18.9	90.7 ± 15.0	91.8 ± 16.8	91.9 ± 14.3	92.3 ± 14.1	93.1 ± 15.2	93.9 ± 16.3	94.9 ± 18.3	95.1 ± 18.4	96.5 ± 20.8	102.1 ± 32.2	<0.0001
Glycated hemoglobin, %	5.77 ± 0.75	5.64 ± 0.63	5.69 ± 0.67	5.70 ± 0.60	5.70 ± 0.58	5.73 ± 0.64	5.76 ± 0.66	5.79 ± 0.75	5.80 ± 0.71	5.86 ± 0.82	6.07 ± 1.22	<0.0001
Triglycerides, mg/dL	104.0 ± 72.0	88.9 ± 65.9	92.6 ± 61.9	92.6 ± 58.3	95.5 ± 61.9	99.7 ± 67.9	103.0 ± 63.7	107.2 ± 75.5	111.1 ± 72.2	117.6 ± 82.8	132.0 ± 92.6	<0.0001
Total cholesterol, mg/dL	198.4 ± 35.9	181.3 ± 33.0	190.0 ± 33.4	193.7 ± 33.8	196.7 ± 35.0	197.9 ± 35.4	201.0 ± 35.2	202.0 ± 36.1	204.1 ± 35.5	206.4 ± 35.8	210.3 ± 36.6	<0.0001
LDL-cholesterol, mg/dL	120.5 ± 31.8	107.9 ± 28.6	113.0 ± 29.6	115.8 ± 29.6	118.3 ± 30.8	119.5 ± 31.1	122.5 ± 31.4	123.3 ± 32.0	125.1 ± 32.0	127.6 ± 32.4	131.7 ± 33.0	<0.0001
HDL-cholesterol, mg/dL	58.2 ± 13.2	56.8 ± 13.1	58.7 ± 13.5	59.4 ± 13.3	59.1 ± 13.1	58.9 ± 13.1	58.6 ± 13.1	58.3 ± 13.3	58.2 ± 13.4	57.6 ± 13.2	56.1 ± 12.9	<0.0001
Uric acid, mg/dL	4.88 ± 1.13	4.49 ± 1.14	4.68 ± 1.08	4.72 ± 1.07	4.78 ± 1.05	4.84 ± 1.09	4.91 ± 1.11	5.01 ± 1.13	5.03 ± 1.10	5.10 ± 1.13	5.30 ± 1.19	<0.0001
Smoking status												<0.0001
Never smoked	44,120 (94.8)	4,535 (94.5)	3,863 (95.6)	4,247 (95.1)	4,314 (94.7)	4,956 (95.1)	5,407 (94.9)	3,199 (95.0)	4,408 (94.8)	5,227 (94.5)	3,954 (93.3)	
Stopped smoked	1,076 (2.3)	112 (2.3)	79 (2.0)	115 (2.6)	120 (2.6)	134 (2.6)	132 (2.3)	80 (2.4)	108 (2.3)	113 (2.0)	83 (2.0)	
Occasionally smoking	40 (0.1)	4 (0.08)	1 (0.02)	0	5 (0.1)	5 (0.1)	4 (0.07)	2 (0.06)	7 (0.2)	3 (0.05)	9 (0.2)	
Currently smoking	1,331 (2.9)	150 (3.1)	100 (2.5)	102 (2.3)	118 (2.6)	116 (2.2)	152 (2.7)	86 (2.6)	126 (2.7)	188 (3.4)	193 (4.6)	
Alcohol drinking status												0.8823
Stopped drinking	381 (0.8)	40 (0.8)	34 (0.8)	37 (0.8)	32 (0.7)	49 (0.9)	45 (0.8)	28 (0.8)	45 (1.0)	43 (0.8)	28 (0.7)	
Occasionally drinking	45,344 (97.4)	4,681 (97.5)	3,952 (97.8)	4,352 (97.5)	4,435 (97.3)	5,063 (97.2)	5,540 (97.3)	3,280 (97.4)	4,522 (97.3)	5,380 (97.3)	4,130 (97.4)	
Currently drinking	840 (1.8)	80 (1.7)	57 (1.4)	75 (1.7)	89 (2.0)	99 (1.9)	110 (1.9)	59 (1.8)	82 (1.8)	108 (2.0)	81 (1.9)	
Physical activity												<0.0001
With regular exercise	18,826 (40.4)	1,469 (30.4)	1,541 (38.1)	1,768 (39.6)	1,845 (40.5)	2,158 (41.4)	2,382 (41.8)	1,419 (42.2)	2,037 (43.8)	2,408 (43.6)	1,799 (42.5)	
Highest educational attainment												<0.0001
No formal teaching or up to primary education	3,370 (7.2)	218 (4.5)	249 (6.2)	318 (7.1)	291 (6.4)	390 (7.5)	429 (7.5)	255 (7.6)	367 (7.9)	468 (8.5)	385 (9.1)	
Secondary education to high school	19,657 (42.2)	1,782 (37.1)	1,550 (38.4)	1,781 (39.9)	1,876 (41.2)	2,155 (41.4)	2,458 (43.2)	1,468 (43.6)	2,107 (45.3)	2,500 (45.2)	1,975 (46.6)	
University to post-graduate studies	23,527 (50.5)	2,801 (58.3)	2,241 (55.5)	2,363 (53.0)	2,389 (52.4)	2,663 (51.1)	2,804 (49.3)	1,644 (48.8)	2,175 (46.8)	2,563 (46.3)	1,878 (44.3)	

Table S2: Characteristics of subjects in the UK Biobank (*N*=386,477) across increasing deciles of Hb levels

**Variable**		**D1**	**D2**	**D3**	**D4**	**D5**	**D6**	**D7**	**D8**	**D9**	**D10**	***P*-value for Trend**
**Men, *n* (%)**	182,048 (47.1)	17,547 (9.6)	18,999 (10.4)	18,073 (9.9)	19,634 (10.8)	16,535 (9.1)	18,664 (10.3)	17,955 (9.9)	18,326 (10.1)	18,021 (9.9)	18,294 (10.1)	
Decile range, g/dL		10.00-13.79	13.80-14.24	14.25-14.54	14.55-14.80	14.81-15.02	15.03-15.29	15.30-15.51	15.52-15.81	15.82-16.23	16.24-20.00	
Hemoglobin, g/dL	15.0 ± 1.0	13.2 ± 0.6	14.0 ± 0.1	14.4 ± 0.1	14.7 ± 0.1	14.9 ± 0.1	15.2 ± 0.1	15.4 ± 0.1	15.7 ± 0.1	16.0 ± 0.1	16.7 ± 0.4	<0.0001
Age, y	56.8 ± 8.1	58.7 ± 7.7	57.4 ± 7.9	56.9 ± 8.0	56.8 ± 8.1	56.4 ± 8.2	56.4 ± 8.1	56.2 ± 8.1	56.1 ± 8.2	56.1 ± 8.2	56.4 ± 8.2	<0.0001
40-49	41,399 (22.7)	2,710 (15.4)	3,793 (20.0)	3,972 (22.0)	4,373 (22.3)	4,023 (24.3)	4,446 (23.8)	4,503 (25.1)	4,602 (25.1)	4,560 (25.3)	4,417 (24.1)	
50-59	59,248 (32.6)	5,140 (29.3)	6,039 (31.8)	5,912 (32.7)	6,440 (32.8)	5,425 (32.8)	6,214 (33.3)	6,061 (33.8)	6,118 (33.4)	5,989 (33.2)	5,910 (32.3)	
60-70	81,401 (44.7)	9,697 (55.3)	9,167 (48.3)	8,189 (45.3)	8,821 (44.9)	7,087 (42.9)	8,004 (42.9)	7,391 (41.2)	7,606 (41.5)	7,472 (41.5)	7,967 (43.6)	
Weight, kg	86.2 ± 14.3	83.9 ± 15.5	84.5 ± 14.3	84.9 ± 13.9	85.6 ± 14.0	86.3 ± 14.1	86.5 ± 14.0	86.7 ± 13.9	87.5 ± 14.1	87.8 ± 14.1	88.7 ± 14.1	<0.0001
Body mass index, kg/m^2	27.9 ± 4.2	27.4 ± 4.8	27.3 ± 4.3	27.4 ± 4.2	27.6 ± 4.2	27.8 ± 4.2	27.9 ± 4.1	28.0 ± 4.1	28.2 ± 4.1	28.4 ± 4.1	28.7 ± 4.1	<0.0001
Waist circumference, cm	97.0 ± 11.3	95.8 ± 12.9	95.5 ± 11.7	95.8 ± 11.2	96.3 ± 11.2	96.9 ± 11.1	97.0 ± 11.0	97.3 ± 10.9	97.9 ± 10.9	98.4 ± 10.8	99.5 ± 10.8	<0.0001
Waist-hip ratio	0.936 ± 0.07	0.929 ± 0.07	0.926 ± 0.07	0.928 ± 0.07	0.931 ± 0.06	0.934 ± 0.06	0.935 ± 0.06	0.937 ± 0.06	0.941 ± 0.06	0.944 ± 0.06	0.952 ± 0.06	<0.0001
Systolic blood pressure, mm Hg	141.1 ± 17.4	138.9 ± 18.3	139.4 ± 17.5	139.7 ± 17.4	140.2 ± 17.2	140.7 ± 17.1	141.3 ± 16.9	141.4 ± 17.1	142.1 ± 17.2	142.9 ± 17.0	144.5 ± 17.4	<0.0001
Diastolic blood pressure, mm Hg	84.2 ± 10.0	79.8 ± 10.0	81.6 ± 9.7	82.4 ± 9.7	83.3 ± 9.7	83.9 ± 9.6	84.6 ± 9.5	85.0 ± 9.6	85.9 ± 9.7	86.7 ± 9.7	88.4 ± 9.8	<0.0001
Glucose, mmol/L	5.11 ± 1.21	5.39 ± 1.66	5.22 ± 1.39	5.16 ± 1.29	5.17 ± 1.35	5.15 ± 1.27	5.16 ± 1.29	5.13 ± 1.34	5.12 ± 1.28	5.13 ± 1.34	5.13 ± 1.43	<0.0001
Glycated hemoglobin, mmol/mol	35.9 ± 6.5	38.3 ± 8.7	36.8 ± 7.1	36.4 ± 6.7	36.3 ± 6.7	36.1 ± 6.9	36.0 ± 6.7	35.9 ± 7.8	35.7 ± 6.7	35.7 ± 7.2	35.6 ± 7.6	<0.0001
Triglycerides, mmol/L	1.98 ± 1.15	1.73 ± 1.06	1.81 ± 1.07	1.87 ± 1.08	1.92 ± 1.12	1.98 ± 1.14	1.99 ± 1.13	2.05 ± 1.17	2.09 ± 1.17	2.14 ± 1.20	2.24 ± 1.23	<0.0001
Total cholesterol, mmol/L	5.50 ± 1.13	5.11 ± 1.13	5.36 ± 1.09	5.45 ± 1.10	5.49 ± 1.11	5.53 ± 1.10	5.57 ± 1.11	5.60 ± 1.12	5.63 ± 1.11	5.64 ± 1.13	5.66 ± 1.14	<0.0001
LDL-cholesterol, mmol/L	3.49 ± 0.86	3.16 ± 0.85	3.36 ± 0.84	3.43 ± 0.84	3.48 ± 0.85	3.51 ± 0.84	3.54 ± 0.85	3.57 ± 0.85	3.60 ± 0.85	3.61 ± 0.86	3.64 ± 0.87	<0.0001
HDL-cholesterol, mmol/L	1.28 ± 0.31	1.32 ± 0.36	1.32 ± 0.33	1.32 ± 0.31	1.30 ± 0.31	1.29 ± 0.31	1.29 ± 0.30	1.27 ± 0.30	1.26 ± 0.29	1.25 ± 0.29	1.22 ± 0.28	<0.0001
Uric acid, μmol/L	309.9 ± 80.4	347.4 ± 79.9	348.0 ± 72.6	350.1 ± 70.6	351.9 ± 70.8	354.4 ± 70.4	355.4 ± 69.9	357.1 ± 69.1	359.0 ± 69.1	359.6 ± 68.7	365.1 ± 70.3	<0.0001
Smoking status												<0.0001
Never smoked	89,406 (49.1)	8,000 (45.6)	9,053 (47.7)	8,864 (49.1)	9,676 (49.3)	8,327 (50.4)	9,420 (50.5)	8,922 (49.7)	9,274 (50.6)	9,083 (50.4)	8,787 (48.1)	
Stopped smoked	70,354 (38.7)	7,677 (43.8)	7,971 (42.0)	7,254 (40.2)	7,779 (39.6)	6,336 (38.3)	7,064 (37.9)	6,771 (37.7)	6,707 (36.6)	6,454 (35.8)	6,341 (34.7)	
Occasionally smoking	6,086 (3.3)	558 (3.2)	638 (3.4)	618 (3.4)	710 (3.6)	598 (3.6)	633 (3.4)	592 (3.3)	606 (3.3)	564 (3.1)	569 (3.1)	
Currently smoking	16,125 (8.9)	1,305 (7.4)	1,327 (7.0)	1,327 (7.4)	1,463 (7.5)	1,268 (7.7)	1,542 (8.3)	1,666 (9.3)	1,729 (9.4)	1,912 (10.6)	2,586 (14.1)	
Alcohol drinking status												<0.0001

Never drank	3,103 (1.7)	416 (2.4)	306 (1.6)	273 (1.5)	297 (1.5)	282 (1.7)	276 (1.5)	293 (1.6)	307 (1.7)	293 (1.6)	360 (2.0)	
Stopped drinking	5,876 (3.2)	907 (5.2)	675 (3.6)	544 (3.0)	588 (3.0)	484 (2.9)	513 (2.8)	527 (2.9)	529 (2.9)	531 (3.0)	578 (3.2)	
Occasionally drinking	11,909 (6.6)	1,392 (7.9)	1,246 (6.6)	1,062 (5.9)	1,134 (5.8)	1,007 (6.1)	1,154 (6.2)	1,100 (6.1)	1,174 (6.4)	1,238 (6.9)	1,402 (7.7)	
Currently drinking	161,013 (88.5)	14,812 (84.5)	16,761 (88.3)	16,177 (89.6)	17,596 (89.7)	14,747 (89.3)	16,711 (89.6)	16,024 (89.3)	16,300 (89.0)	15,949 (88.6)	15,936 (87.2)	
Physical activity												<0.0001
Low	29,042 (18.8)	2,637 (17.9)	2,702 (16.7)	2,665 (17.2)	2,964 (17.6)	2,582 (18.4)	2,904 (18.3)	2,906 (19.1)	3,035 (19.5)	3,114 (20.5)	3,533 (23.2)	
Moderate	59,140 (38.3)	5,407 (36.8)	5,934 (36.6)	5,781 (37.4)	6,285 (37.4)	5,411 (38.5)	6,185 (39.0)	5,921 (38.8)	6,212 (39.8)	5,974 (39.3)	6,030 (39.6)	
High	66,223 (42.9)	6,661 (45.3)	7,571 (46.7)	7,088 (45.4)	7,574 (45.0)	6,081 (43.2)	6,756 (42.6)	6,424 (42.1)	6,349 (40.7)	6,120 (40.2)	5,679 (37.3)	
Highest educational attainment												<0.0001
None	31,680 (17.6)	3,911 (22.5)	3,392 (18.0)	2,960 (16.5)	3,335 (17.1)	2,697 (16.5)	3,008 (16.3)	2,931 (16.5)	2,950 (16.2)	3,052 (17.1)	3,444 (19.0)	
O-levels, CSEs or equivalent	45,621 (25.3)	4,036 (23.2)	4,453 (23.7)	4,343 (24.2)	4,739 (24.3)	4,103 (25.0)	4,818 (26.0)	4,533 (25.4)	4,910 (27.0)	4,761 (26.7)	4,925 (27.2)	
A-levels/AS-levels, NVQ or HNC or equivalent, or other professional qualification	43,950 (24.4)	4,100 (23.6)	4,578 (24.3)	4,289 (23.9)	4,820 (24.8)	3,999 (24.4)	4,377 (23.7)	4,386 (24.6)	4,417 (24.3)	4,434 (24.8)	4,550 (25.1)	
Degree	59,187 (32.8)	5,329 (30.7)	6,399 (34.0)	6,325 (35.3)	6,575 (33.8)	5,600 (34.2)	6,302 (34.1)	5,965 (33.5)	5,888 (32.4)	5,614 (31.4)	5,190 (28.7)	
**Women, *n* (%)**	204,429 (52.9)	20,263 (9.9)	19,723 (9.7)	21,308 (10.4)	20,990 (10.3)	19,744 (9.7)	20,399 (10.0)	19,960 (9.8)	21,279 (10.4)	20,445 (10.0)	20,318 (9.9)	
Decile range, g/dL		8.78-12.39	12.40-12.79	12.80-13.09	13.10-13.30	13.31-13.52	13.53-13.75	13.76-13.99	14.00-14.28	14.29-14.67	14.68-18.05	
Hemoglobin, g/dL	13.5 ± 0.9	11.8 ± 0.6	12.6 ± 0.1	12.9 ± 0.1	13.2 ± 0.1	13.4 ± 0.1	13.6 ± 0.1	13.9 ± 0.1	14.1 ± 0.1	14.5 ± 0.1	15.1 ± 0.4	<0.0001
Age, y	56.4 ± 7.9	54.3 ± 8.3	55.3 ± 8.1	55.7 ± 8.0	56.0 ± 7.9	56.4 ± 7.9	56.5 ± 7.8	56.8 ± 7.7	57.0 ± 7.7	57.5 ± 7.6	58.1 ± 7.4	<0.0001
40-49	47,572 (23.3)	7,072 (34.9)	5,699 (28.9)	5,693 (26.7)	5,206 (24.8)	4,475 (22.7)	4,422 (21.7)	4,078 (20.4)	4,170 (19.6)	3,645 (17.8)	3,113 (15.3)	
50-59	71,140 (34.8)	6,561 (32.4)	6,727 (34.1)	7,435 (34.9)	7,468 (35.6)	7,039 (35.7)	7,309 (35.8)	7,070 (35.4)	7,560 (35.5)	7,051 (34.5)	6,920 (34.1)	
60-70	85,717 (41.9)	6,630 (32.7)	7,297 (37.0)	8,180 (38.4)	8,316 (39.6)	8,230 (41.7)	8,668 (42.5)	8,812 (44.2)	9,549 (44.9)	9,749 (47.7)	10,286 (50.6)	
Had menopause	123,977 (60.6)	9,689 (47.4)	11,025 (56.0)	12,388 (58.2)	12,539 (59.8)	12,142 (61.6)	12,664 (62.1)	12,697 (63.7)	13,603 (64.0)	13,530 (66.2)	13,700 (67.5)	<0.0001
Age at menopause	49.8 ± 5.1	49.4 ± 5.3	49.6 ± 5.1	49.7 ± 5.0	49.7 ± 5.0	49.8 ± 5.0	49.9 ± 5.0	49.9 ± 5.0	50.0 ± 5.0	50.0 ± 5.0	49.9 ± 5.2	<0.0001
Weight, kg	71.4 ± 13.9	69.9 ± 14.3	69.7 ± 13.3	70.2 ± 13.6	70.5 ± 13.5	71.0 ± 13.6	71.2 ± 13.5	71.9 ± 13.7	72.5 ± 14.0	73.1 ± 14.1	74.4 ± 14.8	<0.0001
Body mass index, kg/m^2	27.0 ± 5.1	26.4 ± 5.3	26.3 ± 4.9	26.5 ± 5.0	26.7 ± 5.0	26.9 ± 5.0	26.9 ± 4.9	27.2 ± 5.0	27.4 ± 5.1	27.8 ± 5.2	28.3 ± 5.4	<0.0001
Waist circumference, cm	84.5 ± 12.5	82.5 ± 12.8	82.4 ± 12.0	82.9 ± 12.1	83.4 ± 12.1	83.9 ± 12.1	84.2 ± 12.0	85.0 ± 12.2	85.7 ± 12.4	86.6 ± 12.5	88.3 ± 12.9	<0.0001
Waist-hip ratio	0.816 ± 0.07	0.805 ± 0.07	0.805 ± 0.07	0.807 ± 0.07	0.810 ± 0.07	0.812 ± 0.07	0.815 ± 0.07	0.819 ± 0.07	0.823 ± 0.07	0.828 ± 0.07	0.839 ± 0.07	<0.0001

Systolic blood pressure, mm Hg	135.3 ± 19.4	130.2 ± 18.5	131.6 ± 18.6	132.6 ± 18.7	133.5 ± 18.8	134.7 ± 18.8	135.3 ± 18.8	136.2 ± 18.8	137.6 ± 18.9	139.3 ± 19.1	141.8 ± 19.4	<0.0001
Diastolic blood pressure, mm Hg	80.6 ± 9.9	77.0 ± 9.8	78.0 ± 9.6	78.8 ± 9.6	79.5 ± 9.6	80.1 ± 9.6	80.7 ± 9.6	81.3 ± 9.6	82.2 ± 9.7	83.3 ± 9.7	84.8 ± 9.9	<0.0001
Glucose, mmol/L	5.05 ± 1.03	5.09 ± 1.21	5.02 ± 0.98	5.02 ± 0.97	5.01 ± 0.90	5.04 ± 0.97	5.04 ± 0.93	5.05 ± 0.97	5.06 ± 1.01	5.08 ± 1.05	5.13 ± 1.28	<0.0001
Glycated hemoglobin, mmol/mol	35.6 ± 5.7	36.2 ± 6.5	35.5 ± 5.3	35.4 ± 5.3	35.4 ± 5.1	35.4 ± 5.1	35.4 ± 5.2	35.5 ± 5.3	35.5 ± 5.5	35.6 ± 5.8	36.1 ± 7.2	0.306
Triglycerides, mmol/L	1.55 ± 0.85	1.38 ± 0.78	1.41 ± 0.78	1.44 ± 0.79	1.49 ± 0.81	1.52 ± 0.83	1.55 ± 0.83	1.60 ± 0.87	1.63 ± 0.87	1.69 ± 0.90	1.81 ± 0.96	<0.0001
Total cholesterol, mmol/L	5.89 ± 1.12	5.53 ± 1.06	5.70 ± 1.06	5.79 ± 1.08	5.84 ± 1.10	5.91 ± 1.09	5.94 ± 1.11	5.98 ± 1.11	6.02 ± 1.13	6.08 ± 1.15	6.12 ± 1.18	<0.0001
LDL-cholesterol, mmol/L	3.64 ± 0.87	3.36 ± 0.81	3.47 ± 0.82	3.54 ± 0.83	3.59 ± 0.85	3.64 ± 0.85	3.67 ± 0.86	3.71 ± 0.86	3.74 ± 0.87	3.80 ± 0.88	3.85 ± 0.90	<0.0001
HDL-cholesterol, mmol/L	1.60 ± 0.38	1.58 ± 0.38	1.63 ± 0.37	1.62 ± 0.38	1.62 ± 0.38	1.61 ± 0.37	1.61 ± 0.38	1.60 ± 0.38	1.59 ± 0.37	1.57 ± 0.37	1.52 ± 0.37	<0.0001
Uric acid, μmol/L	270.0 ± 65.5	256.5 ± 69.0	257.5 ± 63.5	260.5 ± 62.3	265.6 ± 63.1	267.4 ± 63.5	269.7 ± 63.3	273.6 ± 64.2	277.1 ± 64.2	281.9 ± 65.5	290.1 ± 68.1	<0.0001
Smoking status												<0.0001
Never smoked	121,645 (59.5)	12,507 (61.8)	12,022 (61.0)	12,834 (60.3)	12,654 (60.3)	11,883 (60.2)	12,177 (59.7)	11,963 (60.0)	12,561 (59.1)	11,913 (58.3)	11,131 (54.8)	
Stopped smoked	64,806 (31.7)	6,566 (32.4)	6,440 (32.7)	7,115 (33.4)	6,787 (32.3)	6,399 (32.4)	6,581 (32.3)	6,265 (31.4)	6,685 (31.4)	6,184 (30.3)	5,784 (28.5)	
Occasionally smoking	4,052 (2.0)	410 (2.0)	392 (1.9)	422 (2.0)	434 (2.1)	395 (2.0)	435 (2.1)	389 (2.0)	430 (2.0)	394 (1.9)	351 (1.7)	
Currently smoking	13,831 (6.8)	769 (3.8)	861 (4.4)	925 (4.3)	1,109 (5.3)	1,061 (5.4)	1,200 (5.9)	1,332 (6.7)	1,586 (7.5)	1,943 (9.5)	3,045 (15.0)	
Alcohol drinking status												0.8823
Never drank	8,955 (4.4)	1,130 (5.6)	871 (4.4)	900 (4.2)	810 (3.9)	831 (4.2)	832 (4.1)	812 (4.1)	901 (4.2)	894 (4.4)	974 (4.8)	
Stopped drinking	7,219 (3.5)	991 (4.9)	710 (3.6)	707 (3.3)	716 (3.4)	642 (3.3)	654 (3.2)	652 (3.3)	734 (3.5)	667 (3.3)	746 (3.7)	
Occasionally drinking	28,538 (14.0)	3,281 (16.2)	2,709 (13.7)	2,896 (13.6)	2,818 (13.4)	2,617 (13.3)	2,646 (13.0)	2,668 (13.4)	2,873 (13.5)	2,897 (14.2)	3,133 (15.4)	
Currently drinking	159,593 (78.1)	14,842 (73.3)	15,421 (78.2)	16,790 (78.9)	16,633 (79.3)	15,645 (79.3)	16,254 (79.7)	15,818 (79.3)	16,755 (78.8)	15,977 (78.2)	15,458 (76.1)	
Physical activity												<0.0001
Low	29,445 (18.5)	3,027 (19.1)	2,810 (18.2)	2,923 (17.4)	3,016 (18.1)	2,718 (17.5)	2,866 (17.9)	2,919 (18.7)	2,988 (18.2)	3,025 (19.3)	3,153 (20.6)	
Moderate	68,427 (42.9)	6,785 (42.7)	6,522 (42.2)	7,236 (43.1)	7,134 (42.8)	6,783 (43.6)	6,890 (43.1)	6,682 (42.7)	7,091 (43.1)	6,719 (43.0)	6,585 (43.0)	
High	61,530 (38.6)	6,078 (38.3)	6,141 (39.7)	6,639 (39.5)	6,501 (39.0)	6,049 (38.9)	6,239 (39.0)	6,035 (38.6)	6,369 (38.7)	5,901 (37.7)	5,578 (36.4)	
Highest educational attainment												<0.0001
None	35,401 (17.5)	3,159 (15.7)	3,117 (15.9)	3,355 (15.9)	3,462 (16.6)	3,238 (16.5)	3,418 (16.9)	3,457 (17.5)	3,723 (17.6)	4,051 (20.0)	4,421 (22.0)	
O-levels, CSEs or equivalent	61,292 (30.2)	6,297 (31.3)	5,770 (29.5)	6,350 (30.1)	6,272 (30.1)	5,946 (30.3)	6,145 (30.4)	5,959 (30.1)	6,331 (30.0)	6,106 (30.2)	6,116 (30.4)	
A-levels/AS-levels, NVQ or HNC or equivalent, or other professional qualification	45,130 (22.3)	4,420 (22.0)	4,307 (22.0)	4,822 (22.8)	4,562 (21.9)	4,343 (22.2)	4,455 (22.0)	4,513 (22.8)	4,728 (22.4)	4,448 (22.0)	4,532 (22.5)	

Degree	60,913 (30.1)	6,220 (31.0)	6,387 (32.6)	6,604 (31.3)	6,533 (31.4)	6,069 (31.0)	6,221 (30.7)	5,871 (29.7)	6,319 (30.0)	5,642 (27.9)	5,047 (25.1)

A-levels, Advanced Level; CSE, Certificate of Secondary Education; HNC, Higher National Certificates; NVQ, National Vocational Qualifications; O-levels, Ordinary Level.

Table S3: Frequency distribution of metabolic outcomes among subjects in the Taiwan Biobank (*N*=67,237) across increasing deciles of Hb levels

Metabolic Outcome		D1	D2	D3	D4	D5	D6	D7	D8	D9	D10	*P*-value for Trend
**Men**	20,670	1,997	2,110	2,250	2,024	2,269	1,535	2,192	2,433	1,761	2,099	
**Hb Decile Range, g/dL**		9.0-13.5	13.6-14.1	14.2-14.5	14.6-14.8	14.9-15.1	15.2-15.3	15.4-15.6	15.7-16.0	16.1-16.4	16.5-20.3	
MS-NCEP ATP III	5,317 (25.7)	407 (20.4)	387 (18.3)	461 (20.5)	421 (20.8)	541 (23.8)	389 (25.3)	586 (26.7)	695 (28.6)	576 (32.7)	854 (40.7)	<0.0001
MS-TW	5,530 (26.8)	425 (21.3)	411 (19.5)	475 (21.1)	434 (21.4)	558 (24.6)	411 (26.8)	621 (28.3)	720 (29.6)	593 (33.7)	882 (42.0)	<0.0001
MS-IDF(C)	4,238 (20.5)	321 (16.1)	296 (14.0)	372 (16.5)	320 (15.8)	422 (18.6)	298 (19.4)	474 (21.6)	557 (22.9)	472 (26.8)	706 (33.6)	<0.0001
Obesity (BMI ≥27.0 kg/m^2)[†]	5,841 (28.3)	404 (20.2)	453 (21.5)	516 (22.9)	494 (24.4)	621 (27.4)	455 (29.6)	679 (31.0)	791 (32.5)	592 (33.6)	836 (39.8)	<0.0001
Overweight (BMI=24.0-26.9 kg/m^2)[†]	7,375 (35.7)	684 (34.3)	708 (33.6)	806 (35.8)	707 (34.9)	821 (36.2)	536 (34.9)	811 (37.0)	854 (35.1)	653 (37.1)	795 (37.9)	
Obesity (BMI ≥30.0 kg/m^2)	1,921 (9.3)	126 (6.3)	129 (6.1)	158 (7.0)	157 (7.8)	182 (8.0)	129 (8.4)	221 (10.1)	269 (11.1)	206 (11.7)	344 (16.4)	<0.0001
Overweight (BMI=25.0-29.9 kg/m^2)	8,636 (41.7)	696 (34.5)	773 (36.6)	877 (39.0)	796 (39.3)	927 (40.9)	667 (43.5)	967 (44.1)	1,079 (44.4)	831 (47.2)	1,023 (48.7)	
Central Obesity (WC >90 cm in men, >80 cm in women)	7,802 (37.8)	596 (29.8)	637 (30.2)	762 (33.9)	676 (33.4)	812 (35.8)	594 (38.7)	870 (39.7)	1,011 (41.6)	777 (44.1)	1,067 (50.8)	<0.0001
Hypertension (SBP/DBP ≥140/≥90 mm Hg)[‡]	5,067 (24.5)	454 (22.7)	423 (20.1)	479 (21.3)	454 (22.4)	526 (23.2)	366 (23.8)	536 (24.5)	632 (26.0)	489 (27.8)	708 (33.7)	<0.0001
Prehypertension (SBP=120-139 mm Hg or DBP=80-89 mm Hg)[‡]	8,980 (43.4)	865 (42.9)	927 (44.0)	964 (42.8)	874 (43.2)	1,000 (44.1)	660 (43.0)	970 (44.3)	1,070 (44.0)	740 (42.0)	910 (43.4)	
Hyperglycemia based on FBG (≥126 mg/dL)	1,333 (6.5)	167 (8.4)	129 (6.1)	141 (6.3)	96 (4.7)	124 (5.5)	73 (4.8)	120 (5.5)	139 (5.7)	128 (7.3)	216 (10.3)	0.0139
Impaired fasting blood glucose (FBG=100-125 mg/dL)	4,573 (22.1)	397 (19.7)	407 (19.3)	471 (20.9)	420 (20.8)	501 (22.1)	355 (23.1)	503 (23.0)	573 (23.6)	405 (23.0)	541 (25.8)	
Hyperglycemia based on HbA1c (≥6.5%)	2,425 (11.7)	316 (15.8)	254 (12.0)	255 (11.3)	201 (9.9)	239 (10.5)	144 (9.4)	221 (10.1)	269 (11.1)	201 (11.4)	325 (15.5)	0.5479
Elevated glycosylated hemoglobin (HbA1c=5.7-6.4%)	9,405 (45.5)	872 (43.2)	967 (45.8)	1,054 (46.8)	937 (46.3)	1,053 (46.4)	703 (45.8)	997 (45.5)	1,090 (44.8)	798 (45.3)	934 (44.5)	
Hypertriglyceridemia (TG ≥200 mg/dL)	3,390 (16.4)	216 (10.8)	230 (10.9)	269 (12.0)	262 (12.9)	331 (14.6)	255 (16.6)	358 (16.3)	486 (20.0)	379 (21.5)	604 (28.8)	<0.0001
Elevated triglycerides (TG=150-199 mg/dL)	2,859 (13.8)	187 (9.3)	240 (11.4)	258 (11.5)	255 (12.6)	299 (13.2)	218 (14.2)	335 (15.3)	384 (15.8)	292 (16.6)	391 (18.6)	
Hypercholesterolemia (TC ≥240 mg/dL)	1,820 (8.8)	89 (4.5)	119 (5.6)	155 (6.9)	157 (7.8)	199 (8.8)	155 (10.1)	217 (9.9)	259 (10.7)	194 (11.0)	276 (13.2)	<0.0001

Elevated total-cholesterol (TC=200-239 mg/dL)	6,110 (29.5)	368 (18.2)	541 (25.6)	617 (27.4)	571 (28.2)	712 (31.4)	506 (33.0)	706 (32.2)	780 (32.1)	580 (32.9)	729 (34.7)	
High LDL-cholesterol (LDL-C ≥160 mg/dL)	2,199 (10.6)	97 (4.9)	141 (6.7)	175 (7.8)	192 (9.5)	248 (10.9)	194 (12.6)	266 (12.1)	313 (12.9)	241 (13.7)	332 (15.8)	<0.0001
Elevated LDL-cholesterol (LDL-C=130-159 mg/dL)	5,515 (26.7)	341 (16.9)	510 (24.2)	557 (24.8)	525 (25.9)	637 (28.1)	433 (28.2)	650 (29.7)	715 (29.4)	527 (29.9)	620 (29.5)	
Low HDL-cholesterol (HDL-C <40 mg/dL in men, <50 mg/dL in women)	4,779 (23.1)	504 (25.2)	408 (19.3)	455 (20.2)	397 (19.6)	482 (21.2)	341 (22.2)	509 (23.2)	574 (23.6)	469 (26.6)	640 (30.5)	<0.0001
Hyperuricemia (SUA >7.0 mg/dL in men, >6.0 mg/dL in women)	6,137 (29.7)	560 (27.8)	532 (25.2)	573 (25.5)	540 (26.7)	671 (29.6)	449 (29.3)	671 (30.6)	779 (32.0)	586 (33.3)	776 (37.0)	<0.0001
**Women**	46,567	4,801	4,043	4,464	4,558	5,211	5,698	3,368	4,650	5,534	4,240	
**Hb Decile Range, g/dL**		6.6-11.5	11.6-12.1	12.2-12.5	12.6-12.8	12.9-13.1	13.2-13.4	13.5-13.6	13.7-13.9	14.0-14.4	14.5-18.7	
MS-NCEP ATP III	8,794 (18.9)	576 (12.0)	543 (13.4)	578 (13.0)	618 (13.6)	838 (16.1)	1,051 (18.5)	679 (20.2)	1,063 (22.9)	1,390 (25.1)	1,458 (34.4)	<0.0001
MS-TW	8,823 (19.0)	582 (12.1)	545 (13.5)	580 (13.0)	620 (13.6)	839 (16.1)	1,056 (18.5)	681 (20.2)	1,067 (23.0)	1,394 (25.2)	1,459 (34.4)	<0.0001
MS-IDF(C)	8,053 (17.3)	521 (10.9)	497 (12.3)	534 (12.0)	557 (12.2)	764 (14.7)	956 (16.8)	627 (18.6)	967 (20.8)	1,277 (23.1)	1,353 (31.9)	<0.0001
Obesity (BMI ≥27.0 kg/m^2)†	7,648 (16.4)	664 (13.8)	518 (12.8)	550 (12.3)	584 (12.8)	708 (13.6)	932 (16.4)	588 (17.5)	819 (17.6)	1,169 (21.1)	1,116 (26.3)	<0.0001
Overweight (BMI=24.0-26.9 kg/m^2)†	10,713 (23.0)	976 (20.3)	794 (19.6)	925 (20.7)	1,013 (22.2)	1,146 (22.0)	1,306 (22.9)	815 (24.2)	1,162 (25.0)	1435 (25.9)	1,141 (26.9)	
Obesity (BMI ≥30.0 kg/m^2)	2,890 (6.2)	276 (5.7)	198 (4.9)	206 (4.6)	191 (4.2)	243 (4.7)	350 (6.1)	221 (6.6)	299 (6.4)	447 (8.1)	459 (10.8)	<0.0001
Overweight (BMI=25.0-29.9 kg/m^2)	11,073 (23.8)	955 (19.7)	779 (19.3)	876 (19.6)	952 (20.9)	1,132 (21.7)	1,335 (23.4)	848 (25.2)	1,225 (26.3)	1,625 (29.4)	1,346 (31.8)	
Central Obesity (WC >90 cm in men, >80 cm in women)	22,310 (47.9)	1,932 (40.2)	1,661 (41.1)	1,846 (41.4)	1,979 (43.4)	2,339 (44.9)	2,737 (48.0)	1,705 (50.6)	2,403 (51.7)	3,074 (55.6)	2,634 (62.1)	<0.0001
Hypertension (SBP/DBP ≥140/≥90 mm Hg)‡	6,053 (13.0)	371 (7.7)	379 (9.4)	385 (8.6)	488 (10.7)	600 (11.5)	723 (12.7)	468 (13.9)	725 (15.6)	939 (17.0)	975 (23.0)	<0.0001
Prehypertension (SBP=120-139 mm Hg or DBP=80-89 mm Hg)‡	13,318 (28.6)	1,046 (21.6)	917 (22.7)	1,130 (25.3)	1,169 (25.7)	1,440 (27.6)	1,684 (29.6)	996 (29.6)	1,465 (31.5)	1,902 (34.4)	1,569 (37.0)	
Hyperglycemia based on FBG (≥126 mg/dL)	1,679 (3.6)	120 (2.5)	113 (2.8)	109 (2.4)	113 (2.5)	148 (2.8)	170 (3.0)	131 (3.9)	173 (3.7)	264 (4.8)	338 (8.0)	<0.0001
Impaired fasting blood glucose (FBG=100-125 mg/dL)	6,286 (13.5)	342 (7.1)	350 (8.7)	442 (9.9)	502 (11.0)	634 (12.2)	806 (14.2)	505 (15.0)	753 (16.2)	991 (17.9)	961 (22.7)	
Hyperglycemia based on HbA1c (≥6.5%)	3,505 (7.5)	241 (5.0)	239 (5.9)	271 (6.1)	262 (5.8)	321 (6.2)	389 (6.8)	277 (8.2)	370 (8.0)	512 (9.3)	623 (14.7)	<0.0001

Elevated glycosylated hemoglobin (HbA1c=5.7-6.4%)	19,617 (42.1)	1,614 (33.3)	1,524 (37.7)	1,805 (40.4)	1,861 (40.8)	2,227 (42.7)	2,477 (43.5)	1,427 (42.4)	2,100 (45.2)	2,569 (46.4)	2,013 (47.5)	
Hypertriglyceridemia (TG ≥200 mg/dL)	3,407 (7.3)	206 (4.3)	208 (5.1)	239 (5.4)	249 (5.5)	311 (6.0)	380 (6.7)	262 (7.8)	384 (8.3)	558 (10.1)	610 (14.4)	<0.0001
Elevated triglycerides (TG=150-199 mg/dL)	4,267 (9.2)	286 (5.9)	293 (7.3)	295 (6.6)	339 (7.4)	422 (8.1)	537 (9.4)	321 (9.5)	519 (11.2)	651 (11.8)	604 (14.2)	
Hypercholesterolemia (TC ≥240 mg/dL)	5,744 (12.3)	236 (4.9)	314 (7.8)	423 (9.5)	524 (11.5)	612 (11.7)	730 (12.8)	453 (13.5)	711 (15.3)	919 (16.6)	822 (19.4)	<0.0001
Elevated total-cholesterol (TC=200-239 mg/dL)	15,720 (33.7)	996 (20.6)	1,118 (27.7)	1,374 (30.8)	1,455 (31.9)	1,751 (33.6)	2,082 (36.5)	1,242 (36.9)	1,763 (37.9)	2,186 (39.5)	1,753 (41.3)	
High LDL-cholesterol (LDL-C ≥160 mg/dL)	5,211 (11.2)	232 (4.8)	270 (6.7)	342 (7.7)	446 (9.8)	526 (10.1)	668 (11.7)	403 (12.0)	621 (13.4)	862 (15.6)	841 (19.8)	<0.0001
Elevated LDL-cholesterol (LDL-C=130-159 mg/dL)	11,701 (25.1)	763 (15.8)	786 (19.4)	972 (21.8)	1,055 (23.2)	1,300 (25.0)	1,528 (26.8)	918 (27.3)	1,373 (29.5)	1,671 (30.2)	1,335 (31.5)	
Low HDL-cholesterol (HDL-C <40 mg/dL in men, <50 mg/dL in women)	12,807 (27.5)	1,465 (30.5)	1,056 (26.1)	1,079 (24.2)	1,103 (24.2)	1,297 (24.9)	1,480 (26.0)	919 (27.3)	1,318 (28.3)	1,666 (30.1)	1,425 (33.6)	<0.0001
Hyperuricemia (SUA >7.0 mg/dL in men, >6.0 mg/dL in women)	6,592 (14.1)	387 (8.0)	418 (10.3)	464 (10.4)	489 (10.7)	648 (12.4)	795 (14.0)	552 (16.4)	765 (16.5)	1,015 (18.3)	1,059 (25.0)	<0.0001

†Taiwan Ministry of Health classification for obesity and overweight. ‡Taiwan Society of Cardiology and Taiwan Hypertension Society classification. BMI, body mass index; DBP, diastolic blood pressure; FBG, fasting blood glucose; HbA1c, glycosylated hemoglobin; HDL-C, high-density lipoprotein cholesterol; LDL-C, low-density lipoprotein cholesterol; MS-IDF(C), metabolic syndrome following International Diabetes Federation criteria for Chinese; MS-NCEP ATP III, metabolic syndrome following National Cholesterol Education Program Adult Treatment Panel III criteria; MS-TW, metabolic syndrome following Taiwan criteria; SBP, systolic blood pressure; SUA, serum uric acid; TC, total cholesterol; TG, triglyceride; WC, waist circumference.

Table S4: Frequency distribution of metabolic outcomes among subjects in the UK Biobank (*N*=386,477) across increasing deciles of Hb levels

**Metabolic Outcome**		**D1**	**D2**	**D3**	**D4**	**D5**	**D6**	**D7**	**D8**	**D9**	**D10**	***P*-value for Trend**
**Men**	182,048	17,547	18,999	18,073	19,634	16,535	18,664	17,955	18,326	18,021	18,294	
**Hb Decile Range, g/dL**		10.00-13.79	13.80-14.24	14.25-14.54	14.55-14.80	14.81-15.02	15.03-15.29	15.30-15.51	15.52-15.81	15.82-16.23	16.24-20.00	
MS-NCEP ATP III	89,330 (49.0)	8,178 (46.3)	8,173 (43.8)	8,178 (44.5)	9,022 (46.0)	7,954 (48.1)	8,899 (48.9)	9,055 (49.1)	9,438 (51.5)	9,748 (54.1)	10,685 (58.4)	<0.0001
MS-IDF	79,412 (43.6)	7,054 (39.9)	7,098 (38.0)	7,189 (39.1)	7,926 (40.4)	7,065 (42.7)	7,896 (43.4)	8,098 (44.0)	8,498 (46.4)	8,874 (49.2)	9,714 (53.1)	<0.0001
Obesity (BMI ≥30.0 kg/m^2)	46,464 (25.5)	4,004 (22.8)	4,065 (21.8)	4,069 (22.2)	4,609 (23.5)	4,067 (24.7)	4,571 (25.2)	4,826 (26.3)	5,083 (27.8)	5,251 (29.2)	5,919 (32.5)	<0.0001
Overweight (BMI=25.0-29.9 kg/m^2)	90,139 (49.6)	7,694 (43.8)	8,682 (46.7)	8,984 (49.0)	9,834 (50.2)	8,335 (50.6)	9,207 (50.7)	9,398 (51.1)	9,404 (51.5)	9,240 (51.4)	9,361 (51.3)	
Central Obesity (WC >94 cm in men, >80 cm in women)	101,772 (55.9)	8,552 (48.7)	9,297 (48.9)	9,213 (51.0)	10,441 (53.2)	9,197 (55.6)	10,480 (56.2)	10,374 (57.8)	10,935 (57.8)	11,161 (61.9)	12,122 (66.3)	<0.0001
Hypertension (SBP/DBP ≥140/≥90 mm Hg)[†]	89,992 (52.8)	7,643 (46.1)	8,243 (47.3)	8,392 (48.6)	9,138 (50.0)	7,975 (51.3)	9,183 (53.3)	9,152 (53.7)	9,527 (55.7)	9,950 (59.0)	10,789 (63.5)	<0.0001
Prehypertension (SBP=130-139 mm Hg or DBP=85-89 mm Hg)[†]	38,376 (22.5)	3,633 (21.9)	3,996 (22.9)	3,958 (22.9)	4,303 (23.6)	3,612 (23.3)	4,026 (23.4)	3,879 (22.7)	3,878 (22.7)	3,613 (21.4)	3,478 (20.5)	
Hyperglycemia based on blood glucose[‡] (≥11.1 mmol/L)	1,803 (1.0)	296 (1.67)	191 (1.02)	144 (0.78)	195 (0.99)	137 (0.83)	155 (0.85)	173 (0.94)	156 (0.85)	172 (0.95)	184 (1.01)	<0.0001
Hyperglycemia based on HbA1c (≥48 mmol/mol)	7,967 (4.4)	1,530 (8.7)	910 (4.9)	765 (4.2)	783 (4.0)	646 (3.9)	687 (3.8)	671 (3.6)	633 (3.5)	650 (3.6)	692 (3.8)	<0.0001
Hypertriglyceridemia (TG ≥2.30 mmol/L)	50,563 (29.0)	3,608 (21.3)	4,256 (23.7)	4,503 (25.5)	5,086 (27.0)	4,558 (28.7)	5,020 (28.8)	5,503 (31.1)	5,673 (32.4)	5,872 (34.0)	6,484 (37.2)	<0.0001
Elevated triglycerides (TG=1.80-2.29 mmol/L)	29,599 (17.0)	2,410 (14.2)	2,736 (15.2)	2,804 (15.9)	3,088 (16.4)	2,749 (17.3)	3,112 (17.9)	3,084 (17.4)	3,153 (18.0)	3,174 (18.4)	3,289 (18.9)	
Hypercholesterolemia (TC ≥6.20 mmol/L)	45,440 (26.0)	2,768 (16.3)	3,881 (21.6)	4,258 (24.1)	4,733 (25.1)	4,186 (26.4)	4,797 (27.5)	5,078 (28.6)	5,167 (29.5)	5,158 (30.0)	5,414 (31.1)	<0.0001
Elevated total-cholesterol (TC=5.20-6.29 mmol/L)	59,227 (33.9)	4,922 (29.1)	5,958 (33.2)	5,949 (33.7)	6,487 (34.4)	5,514 (34.7)	6,093 (34.9)	6,129 (34.6)	6,201 (35.4)	6,018 (34.9)	5,956 (34.2)	
High LDL-cholesterol (LDL-C ≥4.10 mmol/L)	41,243 (23.7)	2,333 (13.8)	3,364 (18.8)	3,760 (21.4)	4,213 (22.4)	3,785 (23.9)	4,433 (25.5)	4,638 (26.2)	4,817 (27.5)	4,794 (27.8)	5,106 (29.4)	<0.0001
Elevated LDL-cholesterol (LDL-C=3.40-4.09 mmol/L)	51,721 (30.0)	4,013 (23.7)	4,984 (27.8)	5,091 (28.9)	5,668 (30.1)	4,794 (30.2)	5,323 (30.6)	5,416 (30.6)	5,483 (31.3)	5,505 (32.0)	5,444 (31.3)	

Low HDL-cholesterol (HDL-C <1.00 mmol/L in men, <1.30 mmol/L in women)	47,892 (26.3)	5,171 (29.3)	4,785 (25.6)	4,533 (24.6)	4,924 (25.1)	4,092 (24.8)	4,637 (25.5)	4,746 (25.8)	4,830 (26.4)	4,828 (26.8)	5,346 (29.2)	0.0006
Hyperuricemia (SUA >416.0 μmol/L in men, >357.0 μmol/L in women)	32,190 (17.7)	2,989 (16.9)	2,943 (15.8)	2,901 (15.8)	3,249 (16.6)	2,833 (17.1)	3,223 (17.7)	3,346 (18.2)	3,440 (18.8)	3,411 (18.9)	3,855 (21.1)	<0.0001
**Women**	204,429	20,263	19,723	21,308	20,990	19,744	20,399	19,960	21,279	20,445	20,318	
**Hb Decile Range, g/dL**		8.78-12.39	12.40-12.79	12.80-13.09	13.10-13.30	13.31-13.52	13.53-13.75	13.76-13.99	14.00-14.28	14.29-14.67	14.68-18.05	
MS-NCEP ATP III	74,714 (37.5)	6,124 (30.0)	5,871 (29.8)	6,618 (31.1)	6,937 (33.1)	6,872 (34.8)	7,474 (36.6)	7,873 (39.4)	8,901 (41.8)	9,281 (45.4)	10,763 (53.0)	<0.0001
MS-IDF	72,248 (35.3)	5,686 (27.8)	5,433 (27.6)	6,181 (29.0)	6,504 (31.0)	6,426 (32.6)	7,034 (34.5)	7,461 (37.4)	8,437 (39.7)	8,818 (43.1)	10,268 (50.5)	<0.0001
Obesity (BMI ≥30.0 kg/m^2)	47,242 (23.1)	4,026 (19.7)	3,617 (18.4)	4,153 (19.5)	4,294 (20.5)	4,259 (21.6)	4,504 (22.1)	4,814 (24.2)	5,410 (25.5)	5,647 (27.7)	6,518 (32.2)	<0.0001
Overweight (BMI=25.0-29.9 kg/m^2)	75,431 (36.9)	6,541 (32.1)	6,950 (35.3)	7,633 (35.9)	7,656 (36.5)	7,318 (37.1)	7,755 (38.1)	7,638 (38.3)	8,252 (38.8)	7,996 (39.2)	7,692 (38.0)	
Central Obesity (WC >94 cm in men, >80 cm in women)	117,475 (57.5)	9,811 (48.4)	9,743 (49.4)	11,073 (52.0)	11,212 (53.4)	10,982 (55.6)	11,715 (57.4)	12,002 (60.1)	13,223 (62.1)	13,394 (65.5)	14,320 (70.5)	<0.0001
Hypertension (SBP/DBP ≥140/≥90 mm Hg)[†]	76,394 (40.0)	5,612 (29.4)	5,847 (31.7)	6,749 (33.9)	6,950 (36.1)	7,025 (38.1)	7,582 (39.7)	7,873 (42.0)	8,992 (45.3)	9,335 (49.0)	10,429 (55.2)	<0.0001
Prehypertension (SBP=130-139 mm Hg or DBP=85-89 mm Hg)[†]	38,153 (20.0)	3,477 (18.2)	3,594 (19.5)	3,881 (19.5)	3,781 (19.7)	3,786 (20.5)	3,936 (20.6)	3,854 (20.6)	4,163 (21.0)	3,932 (20.6)	3,749 (19.9)	
Hyperglycemia based on blood glucose[‡] (≥11.1 mmol/L)	877 (0.4)	137 (0.67)	71 (0.36)	80 (0.38)	57 (0.27)	73 (0.37)	64 (0.31)	73 (0.37)	92 (0.43)	82 (0.40)	148 (0.73)	0.1130
Hyperglycemia based on HbA1c (≥48 mmol/mol)	4,495 (2.2)	731 (3.6)	385 (2.0)	438 (2.1)	379 (1.8)	340 (1.7)	364 (1.8)	360 (1.8)	404 (1.9)	474 (2.3)	620 (3.1)	0.1087
Hypertriglyceridemia (TG ≥2.30 mmol/L)	29,243 (14.9)	2,051 (10.5)	2,055 (10.9)	2,454 (12.0)	2,595 (12.9)	2,622 (13.8)	2,868 (14.6)	3,021 (15.8)	3,470 (17.0)	3,685 (18.8)	4,422 (22.7)	<0.0001
Elevated triglycerides (TG=1.80-2.20 mmol/L)	26,113 (13.3)	2,039 (10.4)	1,994 (10.6)	2,222 (10.9)	2,514 (12.5)	2,435 (12.9)	2,612 (13.3)	2,769 (14.5)	3,049 (14.9)	3,116 (15.9)	3,363 (17.3)	
Hypercholesterolemia (TC ≥6.20 mmol/L)	72,959 (37.2)	4,835 (24.7)	5,671 (30.0)	6,737 (33.0)	7,160 (35.4)	7,084 (37.4)	7,656 (39.1)	7,765 (40.7)	8,630 (42.3)	8,567 (43.7)	8,854 (45.5)	<0.0001
Elevated total-cholesterol (TC=5.20-6.20 mmol/L)	65,500 (34.9)	6,941 (35.4)	6,927 (36.6)	7,469 (36.5)	7,174 (35.5)	6,839 (36.1)	6,802 (34.7)	6,610 (34.6)	6,892 (33.8)	6,604 (33.7)	6,242 (32.1)	
High LDL-cholesterol (LDL-C ≥4.10 mmol/L)	55,095 (28.1)	3,400 (17.4)	3,929 (20.8)	4,842 (23.7)	5,265 (26.1)	5,307 (28.0)	5,737 (29.3)	5,887 (30.9)	6,621 (32.5)	6,808 (34.8)	7,299 (37.6)	<0.0001

Elevated LDL-cholesterol (LDL-C=3.40-4.10 mmol/L)	59,410 (30.3)	5,333 (27.3)	5,630 (29.8)	6,160 (30.2)	6,131 (30.4)	5,842 (30.9)	6,039 (30.9)	5,970 (31.3)	6,394 (31.4)	6,067 (31.0)	5,844 (30.1)	
Low HDL-cholesterol (HDL-C <1.00 mmol/L in men, <1.30 mmol/L in women)	65,764 (32.1)	6,987 (34.2)	5,962 (30.2)	6,521 (30.6)	6,339 (30.2)	6,006 (30.4)	6,226 (30.5)	6,399 (32.1)	6,867 (32.3)	6,798 (33.3)	7,659 (37.7)	<0.0001
Hyperuricemia (SUA >416.0 μmol/L in men, >357.0 μmol/L in women)	18,869 (9.2)	1,514 (7.4)	1,297 (6.6)	1,430 (6.7)	1,614 (7.7)	1,604 (8.1)	1,787 (8.8)	1,889 (9.5)	2,230 (10.5)	2,449 (12.0)	3,055 (15.0)	<0.0001

†European Society of Cardiology/National Institute for Health and Care Excellence classification. ‡Based on non-fasting/random blood biochemistry measured within 24 hours. BMI, body mass index; DBP, diastolic blood pressure; HbA1c, glycosylated hemoglobin; HDL-C, high-density lipoprotein cholesterol; LDL-C, low-density lipoprotein cholesterol; MS-IDF, metabolic syndrome following International Diabetes Federation criteria; MS-NCEP ATP III, metabolic syndrome following National Cholesterol Education Program Adult Treatment Panel III criteria; SBP, systolic blood pressure; SUA, serum uric acid; TC, total cholesterol; TG, triglyceride; WC, waist circumference.

Table S5: Frequency distribution of self-reported metabolic conditions among subjects in the Taiwan Biobank (*N*=67,237) across increasing deciles of Hb levels

**Self-reported Metabolic Conditions**		**D1**	**D2**	**D3**	**D4**	**D5**	**D6**	**D7**	**D8**	**D9**	**D10**	***P*-value for Trend**
**Men**	20,670	1,997	2,110	2,250	2,024	2,269	1,535	2,192	2,433	1,761	2,099	
**Hb Decile Range, g/dL**		9.0-13.5	13.6-14.1	14.2-14.5	14.6-14.8	14.9-15.1	15.2-15.3	15.4-15.6	15.7-16.0	16.1-16.4	16.5-20.3	
Hypertension	3,811 (18.4)	453 (22.7)	432 (20.5)	416 (18.5)	359 (17.7)	413 (18.2)	251 (16.4)	388 (17.7)	403 (16.6)	279 (15.8)	417 (19.9)	<0.0001
Diabetes mellitus	1,532 (7.4)	267 (13.4)	189 (9.0)	174 (7.7)	143 (7.1)	144 (6.4)	81 (5.3)	127 (5.8)	145 (6.0)	99 (5.6)	163 (7.8)	<0.0001
Hyperlipidemia	2,063 (10.0)	245 (12.3)	202 (9.6)	230 (10.2)	213 (10.5)	217 (9.6)	120 (7.8)	219 (10.0)	221 (9.1)	170 (9.7)	226 (10.8)	0.0904
Gout	2,060 (10.0)	217 (10.9)	225 (10.7)	209 (9.3)	175 (8.7)	227 (10.0)	145 (9.5)	242 (11.0)	233 (9.6)	146 (8.3)	241 (11.5)	0.9363
**Women**	46,567	4,801	4,043	4,464	4,558	5,211	5,698	3,368	4,650	5,534	4,240	
**Hb Decile Range, g/dL**		6.6-11.5	11.6-12.1	12.2-12.5	12.6-12.8	12.9-13.1	13.2-13.4	13.5-13.6	13.7-13.9	14.0-14.4	14.5-18.7	
Hypertension	4,915 (10.6)	294 (6.1)	307 (7.6)	407 (9.1)	424 (9.3)	491 (9.4)	587 (10.3)	389 (11.6)	586 (12.6)	737 (13.3)	693 (16.3)	<0.0001
Diabetes mellitus	2,108 (4.5)	189 (3.9)	179 (4.4)	194 (4.4)	207 (4.5)	198 (3.8)	238 (4.2)	141 (4.2)	210 (4.5)	294 (5.3)	258 (6.1)	<0.0001
Hyperlipidemia	3,271 (7.0)	172 (3.6)	212 (5.2)	236 (5.3)	304 (6.7)	397 (7.6)	411 (7.2)	269 (8.0)	384 (8.3)	473 (8.6)	413 (9.7)	<0.0001
Gout	255 (0.55)	22 (0.45)	19 (0.47)	28 (0.63)	23 (0.50)	27 (0.52)	31 (0.54)	13 (0.39)	22 (0.47)	39 (0.70)	31 (0.73)	0.1001

Table S6: Frequency distribution of self-reported metabolic conditions among subjects in the UK Biobank (*N*=386,477) across increasing deciles of Hb levels

**Self-reported Metabolic Conditions**		**D1**	**D2**	**D3**	**D4**	**D5**	**D6**	**D7**	**D8**	**D9**	**D10**	***P*-value for Trend**
**Men**	182,048	17,547	18,999	18,073	19,634	16,535	18,664	17,955	18,326	18,021	18,294	
**Hb Decile Range, g/dL**		10.00-13.79	13.80-14.24	14.25-14.54	14.55-14.80	14.81-15.02	15.03-15.29	15.30-15.51	15.52-15.81	15.82-16.23	16.24-20.00	
Hypertension	63,574 (34.9)	7,458 (42.2)	6,406 (34.3)	6,065 (33.0)	6,512 (33.2)	5,420 (32.8)	6,053 (33.3)	6,026 (32.7)	6,165 (33.6)	6,331 (35.1)	7,138 (39.0)	0.0053
Diagnosis by a doctor†	46,725 (25.7)	4,716 (26.7)	4,373 (23.4)	4,323 (23.5)	4,753 (24.2)	4,034 (24.4)	4,559 (25.1)	4,563 (24.8)	4,777 (26.1)	4,942 (27.4)	5,685 (31.1)	<0.0001
Use of medication‡	17,749 (9.7)	1,736 (9.8)	1,611 (8.6)	1,598 (8.7)	1,766 (9.0)	1,506 (9.1)	1,707 (9.4)	1,693 (9.2)	1,837 (10.0)	1,890 (10.5)	2,405 (13.1)	<0.0001
Type of medication§	46,974 (25.8)	6,274 (35.5)	4,996 (26.8)	4,606 (25.0)	4,821 (24.6)	3,961 (24.0)	4,382 (24.1)	4,244 (23.0)	4,303 (23.5)	4,383 (24.3)	5,004 (27.4)	<0.0001
Non-cancer illness††	54,519 (29.9)	6,179 (35.0)	5,377 (28.8)	5,069 (27.6)	5,541 (28.2)	4,622 (28.0)	5,198 (28.6)	5,228 (28.4)	5,420 (29.6)	5,546 (30.8)	6,339 (34.6)	<0.0001
Diabetes mellitus	11,760 (6.5)	2,375 (13.4)	1,419 (7.6)	1,193 (6.5)	1,142 (5.8)	965 (5.8)	1,011 (5.6)	937 (5.1)	904 (4.9)	873 (4.8)	941 (5.1)	<0.0001
Diagnosis by a doctor†	11,584 (6.4)	2,346 (13.3)	1,401 (7.5)	1,180 (6.4)	1,128 (5.8)	944 (5.7)	993 (5.5)	928 (5.0)	888 (4.9)	861 (4.8)	915 (5.0)	<0.0001
Use of medication‡	336 (0.18)	42 (0.24)	37 (0.20)	38 (0.21)	39 (0.20)	34 (0.21)	29 (0.16)	28 (0.15)	31 (0.17)	32 (0.18)	26 (0.14)	0.0177
Type of medication§	8,420 (4.6)	2,007 (11.4)	1,103 (5.9)	886 (4.8)	829 (4.2)	663 (4.0)	675 (3.7)	597 (3.2)	600 (3.3)	555 (3.0)	505 (2.8)	<0.0001
Non-cancer illness††	11,349 (6.2)	2,305 (13.0)	1,371 (7.3)	1,151 (6.3)	1,107 (5.6)	930 (5.6)	965 (5.3)	903 (4.9)	874 (4.8)	843 (4.7)	900 (4.9)	<0.0001
Hyperlipidemia	44,575 (24.5)	6,123 (34.6)	5,052 (27.1)	4,612 (25.1)	4,793 (24.4)	3,850 (23.3)	4,255 (23.4)	4,035 (21.9)	3,977 (21.7)	3,937 (21.9)	3,941 (21.5)	<0.0001
Use of medication‡	41,008 (22.5)	5,719 (32.4)	4,675 (25.0)	4,239 (23.0)	4,427 (22.6)	3,555 (21.5)	3,895 (21.4)	3,681 (20.0)	3,654 (19.9)	3,591 (19.9)	3,572 (19.5)	<0.0001
Type of medication§	39,886 (21.9)	5,550 (31.4)	4,584 (24.5)	4,134 (22.5)	4,329 (22.1)	3,427 (20.7)	3,783 (20.8)	3,574 (19.4)	3,512 (19.2)	3,522 (19.5)	3,471 (19.0)	<0.0001
Non-cancer illness††	27,512 (15.1)	3,393 (19.2)	3,086 (16.5)	2,848 (15.5)	2,951 (15.0)	2,433 (14.7)	2,726 (15.0)	2,523 (13.7)	2,531 (13.8)	2,497 (13.9)	2,524 (13.8)	<0.0001
Gout	5,770 (3.2)	731 (4.1)	637 (3.4)	551 (3.0)	613 (3.1)	496 (3.0)	551 (3.0)	526 (2.9)	549 (3.0)	542 (3.0)	574 (3.1)	<0.0001
Type of medication§	4,179 (2.3)	581 (3.3)	466 (2.5)	412 (2.2)	426 (2.2)	370 (2.2)	381 (2.1)	357 (1.9)	383 (2.1)	374 (2.1)	429 (2.3)	<0.0001
Non-cancer illness††	5,209 (2.9)	627 (3.6)	575 (3.1)	506 (2.8)	558 (2.8)	456 (2.8)	503 (2.8)	479 (2.6)	492 (2.7)	495 (2.8)	518 (2.8)	<0.0001
**Women**	204,429	20,263	19,723	21,308	20,990	19,744	20,399	19,960	21,279	20,445	20,318	
**Hb Decile Range, g/dL**		8.78-12.39	12.40-12.79	12.80-13.09	13.10-13.30	13.31-13.52	13.53-13.75	13.76-13.99	14.00-14.28	14.29-14.67	14.68-18.05	
Hypertension	54,087 (26.4)	4,792 (23.4)	4,391 (22.3)	4,745 (22.3)	5,031 (24.0)	4,807 (24.4)	5,084 (24.9)	5,352 (26.8)	6,023 (28.3)	6,409 (31.4)	7,453 (36.7)	<0.0001
Diagnosis by a doctor†	44,754 (21.9)	3,628 (17.8)	3,488 (17.7)	3,811 (17.9)	4,079 (19.4)	4,008 (20.3)	4,234 (20.8)	4,521 (22.7)	5,103 (24.0)	5,484 (26.8)	6,398 (31.5)	<0.0001
Use of medication‡	20,305 (9.9)	1,579 (7.7)	1,556 (7.9)	1,717 (8.1)	1,769 (8.4)	1,860 (9.4)	1,906 (9.3)	1,999 (10.0)	2,340 (11.0)	2,485 (12.2)	3,094 (15.2)	<0.0001
Type of medication§	38,685 (18.9)	3,618 (17.7)	3,176 (16.1)	3,398 (16.0)	3,558 (17.0)	3,374 (17.1)	3,591 (17.6)	3,723 (18.7)	4,257 (20.0)	4,511 (22.1)	5,479 (27.0)	<0.0001

Non-cancer illness††	46,643 (22.8)	3,915 (19.2)	3,715 (18.8)	4,008 (18.8)	4,280 (20.4)	4,141 (21.0)	4,390 (21.5)	4,652 (23.3)	5,265 (24.7)	5,618 (27.5)	6,659 (32.8)	<0.0001
Diabetes mellitus	6,910 (3.4)	1,106 (5.4)	673 (3.4)	688 (3.2)	595 (2.8)	586 (3.0)	581 (2.9)	574 (2.9)	618 (2.9)	687 (3.4)	802 (4.0)	<0.0001
Diagnosis by a doctor†	6,745 (3.3)	1,088 (5.3)	662 (3.4)	670 (3.1)	577 (2.8)	570 (2.9)	563 (2.8)	557 (2.8)	596 (2.8)	675 (3.3)	787 (3.9)	<0.0001
Use of medication‡	242 (0.12)	38 (0.19)	19 (0.10)	36 (0.17)	21 (0.10)	22 (0.11)	27 (0.13)	20 (0.10)	24 (0.11)	19 (0.09)	16 (0.08)	0.0039
Type of medication§	4,565 (2.2)	910 (4.5)	480 (2.4)	472 (2.2)	399 (1.9)	381 (1.9)	387 (1.8)	350 (1.8)	365 (1.7)	400 (2.0)	421 (2.1)	<0.0001
Non-cancer illness††	6,011 (2.9)	980 (4.8)	581 (3.0)	590 (2.8)	524 (2.5)	495 (2.5)	504 (2.5)	480 (2.4)	534 (2.5)	608 (3.0)	715 (3.5)	<0.0001
Hyperlipidemia	28,136 (13.8)	2,736 (13.4)	2,441 (12.4)	2,643 (12.4)	2,723 (13.0)	2,439 (12.4)	2,657 (13.0)	2,770 (13.9)	3,046 (14.3)	3,125 (15.3)	3,556 (17.5)	<0.0001
Use of medication‡	25,370 (12.4)	2,488 (12.2)	2,218 (11.3)	2,374 (11.1)	2,443 (11.6)	2,169 (11.0)	2,418 (11.9)	2,503 (12.5)	2,746 (12.9)	2,794 (13.7)	3,217 (15.8)	<0.0001
Type of medication§	24,300 (11.9)	2,388 (11.7)	2,112 (10.7)	2,251 (10.6)	2,361 (11.3)	2,056 (10.4)	2,322 (11.4)	2,380 (11.9)	2,636 (12.4)	2,717 (13.3)	3,077 (15.1)	<0.0001
Non-cancer illness††	19,687 (9.6)	1,682 (8.2)	1,645 (8.3)	1,836 (8.6)	1,921 (9.2)	1,772 (9.0)	1,872 (9.2)	1,973 (9.9)	2,158 (10.1)	2,267 (11.1)	2,561 (12.6)	<0.0001
Gout	436 (0.21)	61 (0.30)	44 (0.22)	36 (0.17)	42 (0.20)	34 (0.17)	39 (0.19)	38 (0.19)	39 (0.18)	40 (0.20)	63 (0.31)	0.9663
Type of medication§	310 (0.15)	46 (0.23)	35 (0.18)	28 (0.13)	28 (0.13)	25 (0.13)	25 (0.12)	24 (0.12)	26 (0.12)	27 (0.13)	46 (0.23)	0.4273
Non-cancer illness††	363 (0.18)	47 (0.23)	33 (0.17)	34 (0.16)	36 (0.17)	27 (0.14)	36 (0.18)	33 (0.17)	32 (0.15)	35 (0.17)	50 (0.25)	0.7946

†Data fields 6150 and 2443 for self-reported diagnosis of high blood pressure and diabetes by a doctor. ‡Data fields 6177 and 6153 for self-reported use of medications. §Data field 20003 for type of prescription medications by verbal interview. ††Data field 20002 for self-reported non-cancer illnesses (hypertension, essential hypertension, diabetes, type 2 diabetes, high cholesterol, and gout).

Table S7: Cases of metabolic disorders from hospital in-patient records of UK Biobank subjects (*N*=386,477) across increasing deciles of Hb levels

**Diagnoses†**		**D1**	**D2**	**D3**	**D4**	**D5**	**D6**	**D7**	**D8**	**D9**	**D10**	***P*-value for Trend**
**Men**	182,048	17,547	18,999	18,073	19,634	16,535	18,664	17,955	18,326	18,021	18,294	
**Hb Decile Range, g/dL**		10.00-13.79	13.80-14.24	14.25-14.54	14.55-14.80	14.81-15.02	15.03-15.29	15.30-15.51	15.52-15.81	15.82-16.23	16.24-20.00	
Hypertension	46,142 (25.3)	6,012 (34.0)	4,720 (25.3)	4,397 (23.9)	4,710 (24.0)	3,879 (23.5)	4,341 (23.9)	4,185 (22.7)	4,320 (23.6)	4,427 (24.6)	5,151 (28.2)	<0.0001
ICD9 diagnoses	85 (0.05)	16 (0.09)	5 (0.03)	7 (0.04)	7 (0.04)	6 (0.04)	10 (0.05)	10 (0.05)	4 (0.02)	9 (0.05)	11 (0.06)	0.7285
ICD10 diagnoses	46,108 (25.3)	6,008 (34.0)	4,718 (25.3)	4,394 (23.9)	4,707 (24.0)	3,878 (23.5)	4,335 (23.8)	4,181 (22.7)	4,318 (23.6)	4,424 (24.6)	5,145 (28.1)	<0.0001
Diabetes Mellitus	13,561 (17.4)	2,496 (14.1)	1,470 (7.9)	1,235 (6.7)	1,289 (6.6)	1,076 (6.5)	1,180 (6.5)	1,138 (6.2)	1,138 (6.2)	1,156 (6.4)	1,383 (7.6)	<0.0001
ICD9 diagnoses	58 (0.03)	17 (0.10)	4 (0.02)	7 (0.04)	5 (0.03)	4 (0.02)	5 (0.03)	5 (0.03)	2 (0.01)	4 (0.02)	5 (0.03)	0.0026
ICD10 diagnoses	13,548 (7.5)	2,494 (14.1)	1,469 (7.9)	1,233 (6.7)	1,287 (6.6)	1,075 (6.5)	1,179 (6.5)	1,136 (6.2)	1,138 (6.4)	1155 (14.1)	1,382 (7.6)	<0.0001
Lipids Disorders	24,390 (13.4)	1,617 (7.9)	1,328 (6.7)	1,478 (6.9)	1,475 (7.0)	1,316 (6.7)	1,412 (6.9)	1,407 (7.1)	1,556 (7.3)	1,646 (8.1)	1,992 (9.8)	<0.0001
ICD9 diagnoses	28 (0.02)	6 (0.03)	3 (0.02)	2 (0.01)	5 (0.03)	2 (0.01)	1 (0.01)	1 (0.01)	3 (0.02)	1 (0.01)	4 (0.02)	0.1903
ICD10 diagnoses	24,373 (13.4)	3,370 (19.1)	2,699 (14.5)	2,439 (13.3)	2,568 (13.1)	2,119 (12.8)	2,295 (12.6)	2,209 (12.0)	2,180 (11.9)	2,241 (12.4)	2,253 (12.3)	<0.0001
Gout	3,568 (2.0)	602 (3.4)	391 (2.1)	333 (1.8)	357 (1.8)	267 (1.6)	321 (1.8)	302 (1.6)	291 (1.6)	324 (1.8)	380 (2.1)	<0.0001
ICD9 diagnoses	7	3 (0.03)	2 (0.01)	0	0	0	0	0	0	1 (0.01)	1 (0.01)	-
ICD10 diagnoses	3,562 (2.0)	600 (3.4)	389 (2.1)	333 (1.8)	357 (1.8)	267 (1.6)	321 (1.8)	302 (1.6)	291 (1.6)	323 (1.8)	379 (2.1)	<0.0001
**Women**	204,429	20,263	19,723	21,308	20,990	19,744	20,399	19,960	21,279	20,445	20,318	
**Hb Decile Range, g/dL**		8.78-12.39	12.40-12.79	12.80-13.09	13.10-13.30	13.31-13.52	13.53-13.75	13.76-13.99	14.00-14.28	14.29-14.67	14.68-18.05	
Hypertension	37,706 (18.4)	3,709 (18.1)	3,220 (16.3)	3,280 (15.4)	3,470 (16.5)	3,352 (17.0)	3,483 (17.1)	3,619 (18.1)	4,103 (19.3)	4,245 (20.8)	5,225 (25.7)	<0.0001
ICD9 diagnoses	69 (0.03)	8 (0.04)	4 (0.02)	9 (0.04)	9 (0.04)	5 (0.03)	4 (0.02)	3 (0.02)	9 (0.04)	9 (0.04)	9 (0.04)	0.6071
ICD10 diagnoses	37,666 (18.4)	3,707 (18.1)	3,218 (16.2)	3,275 (15.4)	3,465 (16.5)	3,350 (17.0)	3,479 (17.1)	3,617 (18.1)	4,098 (19.3)	4,237 (20.7)	5,220 (25.7)	<0.0001
Diabetes Mellitus	8,069 (3.9)	1,194 (5.8)	727 (3.7)	709 (3.3)	680 (3.2)	637 (3.2)	680 (3.3)	667 (3.3)	740 (3.5)	862 (4.2)	1173 (5.8)	0.0895
ICD9 diagnoses	44 (0.02)	10 (0.05)	9 (0.05)	4 (0.02)	3 (0.01)	5 (0.03)	3 (0.01)	2 (0.01)	1	1	6 (0.03)	0.0032
ICD10 diagnoses	8,053 (3.9)	1,194 (5.8)	724 (3.7)	707 (3.3)	678 (3.2)	633 (3.2)	678 (3.3)	667 (3.3)	740 (3.5)	862 (4.2)	1,170 (5.8)	0.0848
Lipids Disorders	15,227 (7.4)	1,617 (7.9)	1,328 (6.7)	1,478 (6.9)	1,475 (7.0)	1,316 (6.7)	1,412 (6.9)	1,407 (7.1)	1,556 (7.3)	1,646 (8.1)	1,992 (9.8)	<0.0001
ICD9 diagnoses	19 (0.01)	1	2 (0.01)	1	2 (0.01)	0	1	2 (0.01)	2 (0.01)	3 (0.01)	5 (0.02)	0.0508
ICD10 diagnoses	15,213 (7.4)	1,616 (7.9)	1,326 (6.7)	1,477 (6.9)	1,473 (7.0)	1,316 (6.7)	1,411 (6.9)	1,407 (7.1)	1,555 (7.3)	1,644 (8.0)	1,988 (9.8)	<0.0001

Gout	498 (0.24)	83 (0.41)	42 (0.21)	35 (0.16)	51 (0.24)	48 (0.24)	38 (0.19)	46 (0.23)	43 (0.20)	51 (0.25)	61 (0.30)	0.3345
ICD9 diagnoses	1	0	0	0	0	0	0	0	1	0	0	-
ICD10 diagnoses	497 (0.24)	83 (0.41)	42 (0.21)	35 (0.16)	51 (0.24)	48 (0.24)	38 (0.19)	46 (0.23)	42 (0.20)	51 (0.25)	61 (0.30)	0.3148

†Data fields 41203 and 41205 provided diagnoses data from the hospital in-patient records of a subject, which are coded according to the International Classification of Diseases version-9 (ICD-9). Data fields 41202 and 41204 provided diagnoses data from the hospital in-patient records of a subject, which are coded according to the International Classification of Diseases version-10 (ICD-10).

Table S8: Cross-tabulation of cases in the Taiwan Biobank based on self-reported metabolic disorders and high levels of biochemical markers

Metabolic Outcome	All (*N*=67,237)	Men (*n*=20,670)	Women (*n*=46,567)
**A. Central obesity (based on ethnic-specific WC cutoff)**	**30,112 (44.8)**	**7,802 (37.8)**	**22,310 (47.9)**
**B. Hypertension**	**16,078 (23.9)**	**7,076 (34.2)**	**9,002 (19.3)**
High blood pressure	11,124 (16.5)	5,070 (24.5)	6,054 (13.0)
Self-reported hypertension	8,730 (13.0)	3,815 (18.4)	4,915 (10.6)
**C. Diabetes mellitus**	**6,875 (10.2)**	**2,810 (13.6)**	**4,065 (8.7)**
High glucose or HbA1c	6,170 (9.2)	2,532 (12.2)	3,638 (7.8)
Self-reported diabetes	3,641 (5.4)	1,533 (7.4)	2,108 (4.5)
**D. Dyslipidemia**	**23,066 (34.3)**	**7,505 (36.3)**	**15,561 (33.4)**
Hypertriglyceridemia or low HDL-C	20,039 (29.8)	6,383 (30.9)	13,656 (29.3)
Self-reported hyperlipidemia	5,335 (7.9)	2,064 (10.0)	3,271 (7.0)
**E. Gout**	**13,580 (20.2)**	**6,876 (33.3)**	**6,704 (14.4)**
Hyperuricemia	12,729 (18.9)	6,137 (29.7)	6,592 (14.1)
Self-reported gout	2,318 (3.4)	2,063 (9.8)	255 (0.6)

HbA1c, glycated hemoglobin; HDL-C, high-density lipoprotein cholesterol; LDL-C, low-density lipoprotein cholesterol; WC, waist circumference.

Table S9: Cross-tabulation of cases in the UK Biobank based on high levels of anthropometric and biochemical markers, self-reported metabolic conditions, and hospital in-patient records of subjects

Metabolic Disorder	All (*N*=386,477)	Men (*n*=182,048)	Women (*n*=204,429)
**A. Central obesity (based on ethnic-specific WC cutoff)**	**219,247 (56.7)**	**101,772 (55.9)**	**117,475 (57.5)**
**B. Hypertension**	**218,759 (56.6)**	**116,701 (64.1)**	**102,058 (49.9)**
High blood pressure	166,386 (43.0)	89,992 (49.4)	76,394 (37.3)
Self-reported hypertension	117,661 (30.4)	63,574 (34.9)	54,087 (26.4)
ICD diagnoses	83,848 (21.7)	46,142 (25.3)	37,706 (18.4)
**C. Diabetes mellitus**	**27,750 (7.2)**	**17,180 (9.4)**	**10,570 (5.2)**
High glucose or HbA1c	12,725 (3.3)	8,158 (4.5)	4,567 (2.2)
Self-reported diabetes	18,670 (4.8)	11,760 (6.5)	6,910 (3.4)
ICD diagnoses	21,630 (5.6)	13,561 (7.4)	8,069 (3.9)
**D. Dyslipidemia**	**200,565 (51.9)**	**105,275 (57.8)**	**95,290 (46.6)**
Hypertriglyceridemia or low HDL-C	158,593 (41.0)	79,747 (43.8)	78,846 (38.6)
Self-reported dyslipidemia	72,711 (18.8)	44,575 (24.5)	28,136 (13.8)
ICD diagnoses	39,617 (10.2)	24,390 (13.4)	15,227 (7.4)
**E. Gout**	**55,706 (14.4)**	**36,401 (20.0)**	**19,305 (9.4)**
Hyperuricemia	51,059 (13.2)	32,190 (17.7)	18,869 (9.2)
Self-reported gout	6,206 (1.6)	5,770 (3.2)	436 (0.21)
ICD diagnoses	4,066 (1.1)	3,568 (2.0)	498 (0.24)

HbA1c, glycated hemoglobin; HDL-C, high-density lipoprotein cholesterol; ICD, International Classification of Diseases; LDL-C, low-density lipoprotein cholesterol; WC, waist circumference.

Table S10: Odds ratios of metabolic syndrome (NCEP ATP III criteria) across increasing deciles of hemoglobin levels among Taiwanese Han Chinese

Metabolic Syndrome	Cases (%)	Model 1†	*P*-value	Model 2‡	*P*-value	Model 3§	*P*-value
**Men (*n*=20,670)**	5,317 (25.7)						
D1 (<13.6 g/dL) vs. D2	407 (20.4)	1.11 (0.95, 1.29)	0.2016	1.10 (0.94, 1.29)	0.2371	1.09 (0.93, 1.27)	0.2979
D2 (13.6-14.1 g/dL)	387 (18.3)	Reference	-	Reference	-	Reference	-
D3 (14.2-14.5 g/dL) vs. D2	461 (20.5)	1.18 (1.02, 1.38)	**0.0307**	1.17 (1.01, 1.36)	**0.0407**	1.17 (1.00, 1.36)	**0.0451**
D4 (14.6-14.8 g/dL) vs. D2	421 (20.8)	1.22 (1.05, 1.42)	**0.0119**	1.21 (1.03, 1.41)	**0.0180**	1.20 (1.03, 1.40)	**0.0221**
D5 (14.9-15.1 g/dL) vs. D2	541 (23.8)	1.47 (1.27, 1.71)	**<0.0001**	1.46 (1.26, 1.69)	**<0.0001**	1.45 (1.25, 1.68)	**<0.0001**
D6 (15.2-15.3 g/dL) vs. D2	389 (25.3)	1.63 (1.39, 1.91)	**<0.0001**	1.61 (1.37, 1.89)	**<0.0001**	1.59 (1.36, 1.87)	**<0.0001**
D7 (15.4-15.6 g/dL) vs. D2	586 (26.7)	1.78 (1.54, 2.06)	**<0.0001**	1.75 (1.51, 2.03)	**<0.0001**	1.74 (1.50, 2.01)	**<0.0001**
D8 (15.7-16.0 g/dL) vs. D2	695 (28.6)	1.96 (1.70, 2.26)	**<0.0001**	1.92 (1.66, 2.22)	**<0.0001**	1.90 (1.65, 2.20)	**<0.0001**
D9 (16.1-16.4 g/dL) vs. D2	576 (32.7)	2.46 (2.12, 2.86)	**<0.0001**	2.37 (2.04, 2.76)	**<0.0001**	2.36 (2.02, 2.74)	**<0.0001**
D10 (>16.4 g/dL) vs. D2	854 (40.7)	3.44 (2.98, 3.97)	**<0.0001**	3.22 (2.79, 3.72)	**<0.0001**	3.17 (2.75, 3.67)	**<0.0001**
**Women¶ (*n*=46,567)**	8,794 (18.9)						
D1 (<11.6 g/dL) vs. D3	576 (12.0)	1.13 (0.99, 1.28)	0.0664	1.13 (0.99, 1.28)	0.0627	1.11 (0.98, 1.26)	0.1147
D2 (11.6-12.1 g/dL) vs. D3	543 (13.4)	1.10 (0.97, 1.25)	0.1471	1.10 (0.97, 1.25)	0.1416	1.11 (0.97, 1.26)	0.1195
D3 (12.2-12.5 g/dL)	578 (13.0)	Reference	-	Reference	-	Reference	-
D4 (12.6-12.8 g/dL) vs. D3	618 (13.6)	1.02 (0.90, 1.16)	0.7217	1.02 (0.90, 1.16)	0.7283	1.03 (0.91, 1.17)	0.6443
D5 (12.9-13.1 g/dL) vs. D3	838 (16.1)	1.23 (1.09, 1.38)	**0.0006**	1.23 (1.09, 1.38)	**0.0006**	1.23 (1.10, 1.39)	**0.0005**
D6 (13.2-13.4 g/dL) vs. D3	1,051 (18.5)	1.43 (1.28, 1.60)	**<0.0001**	1.43 (1.28, 1.60)	**<0.0001**	1.44 (1.29, 1.61)	**<0.0001**
D7 (13.5-13.6 g/dL) vs. D3	679 (20.2)	1.59 (1.40, 1.80)	**<0.0001**	1.59 (1.40, 1.80)	**<0.0001**	1.59 (1.41, 1.80)	**<0.0001**
D8 (13.7-13.9 g/dL) vs. D3	1,063 (22.9)	1.82 (1.62, 2.03)	**<0.0001**	1.81 (1.62, 2.03)	**<0.0001**	1.83 (1.63, 2.05)	**<0.0001**
D9 (14.0-14.4 g/dL) vs. D3	1,390 (25.1)	2.05 (1.84, 2.28)	**<0.0001**	2.04 (1.83, 2.28)	**<0.0001**	2.06 (1.84, 2.29)	**<0.0001**
D10 (>14.4 g/dL) vs. D3	1,458 (34.4)	3.11 (2.79, 3.48)	**<0.0001**	3.09 (2.76, 3.45)	**<0.0001**	3.11 (2.78, 3.47)	**<0.0001**

†Model 1 adjusted for age and age-squared. ‡Model 2 additionally adjusted for smoking and alcohol consumption. §Model 3 additionally adjusted for physical activity and highest educational attainment. ¶In women, menopausal status was included as covariate.

Table S10.1: Odds ratios of metabolic syndrome (Taiwan's criteria) across increasing deciles of hemoglobin levels among Taiwanese Han Chinese

Metabolic Syndrome	Cases (%)	Model 1†	*P*-value	Model 2‡	*P*-value	Model 3§	*P*-value
**Men (*n*=20,670)**	5,530 (26.8)						
D1 (<13.6 g/dL) vs. D2	425 (21.3)	1.09 (0.93, 1.27)	0.2846	1.08 (0.93, 1.26)	0.3308	1.07 (0.92, 1.25)	0.4028
D2 (13.6-14.1 g/dL)	411 (19.5)	Reference	-	Reference	-	Reference	-
D3 (14.2-14.5 g/dL) vs. D2	475 (21.1)	1.14 (0.98, 1.32)	0.0898	1.13 (0.97, 1.31)	0.1149	1.12 (0.97, 1.31)	0.1251
D4 (14.6-14.8 g/dL) vs. D2	434 (21.4)	1.17 (1.01, 1.37)	**0.0390**	1.16 (1.00, 1.35)	0.0557	1.16 (0.99, 1.35)	0.0658
D5 (14.9-15.1 g/dL) vs. D2	558 (24.6)	1.42 (1.23, 1.64)	**<0.0001**	1.41 (1.22, 1.63)	**<0.0001**	1.40 (1.21, 1.62)	**<0.0001**
D6 (15.2-15.3 g/dL) vs. D2	411 (26.8)	1.62 (1.39, 1.90)	**<0.0001**	1.60 (1.37, 1.87)	**<0.0001**	1.59 (1.36, 1.86)	**<0.0001**
D7 (15.4-15.6 g/dL) vs. D2	621 (28.3)	1.78 (1.54, 2.05)	**<0.0001**	1.75 (1.52, 2.03)	**<0.0001**	1.74 (1.50, 2.01)	**<0.0001**
D8 (15.7-16.0 g/dL) vs. D2	720 (29.6)	1.90 (1.65, 2.19)	**<0.0001**	1.86 (1.62, 2.15)	**<0.0001**	1.85 (1.61, 2.13)	**<0.0001**
D9 (16.1-16.4 g/dL) vs. D2	593 (33.7)	2.37 (2.04, 2.75)	**<0.0001**	2.29 (1.97, 2.66)	**<0.0001**	2.27 (1.96, 2.64)	**<0.0001**
D10 (>16.4 g/dL) vs. D2	882 (42.0)	3.36 (2.92, 3.86)	**<0.0001**	3.14 (2.73, 3.62)	**<0.0001**	3.10 (2.69, 3.57)	**<0.0001**
**Women¶ (*n*=46,567)**	8,823 (19.0)						
D1 (<11.6 g/dL)	582 (12.1)	1.13 (1.00, 1.29)	0.0517	1.14 (1.001, 1.29)	**0.0487**	1.12 (0.98, 1.27)	0.0919
D2 (11.6-12.1 g/dL) vs. D1	545 (13.5)	1.10 (0.97, 1.25)	0.1467	1.10 (0.97, 1.25)	0.1413	1.11 (0.97, 1.26)	0.1191
D3 (12.2-12.5 g/dL) vs. D1	580 (13.0)	Reference	-	Reference	-	Reference	-
D4 (12.6-12.8 g/dL) vs. D1	620 (13.6)	1.02 (0.90, 1.16)	0.7231	1.02 (0.90, 1.16)	0.7298	1.03 (0.91, 1.17)	0.6461
D5 (12.9-13.1 g/dL) vs. D1	839 (16.1)	1.22 (1.09, 1.37)	**0.0007**	1.22 (1.09, 1.38)	**0.0007**	1.23 (1.09, 1.38)	**0.0005**
D6 (13.2-13.4 g/dL) vs. D1	1,056 (18.5)	1.43 (1.28, 1.60)	**<0.0001**	1.43 (1.28, 1.60)	**<0.0001**	1.44 (1.29, 1.61)	**<0.0001**
D7 (13.5-13.6 g/dL) vs. D1	681 (20.2)	1.59 (1.40, 1.80)	**<0.0001**	1.59 (1.40, 1.80)	**<0.0001**	1.59 (1.41, 1.80)	**<0.0001**
D8 (13.7-13.9 g/dL) vs. D1	1,067 (23.0)	1.82 (1.62, 2.04)	**<0.0001**	1.81 (1.62, 2.03)	**<0.0001**	1.83 (1.64, 2.05)	**<0.0001**
D9 (14.0-14.4 g/dL) vs. D1	1,394 (25.2)	2.05 (1.84, 2.28)	**<0.0001**	2.04 (1.83, 2.28)	**<0.0001**	2.06 (1.84, 2.29)	**<0.0001**
D10 (>14.4 g/dL) vs. D1	1,459 (34.4)	3.11 (2.78, 3.47)	**<0.0001**	3.08 (2.76, 3.44)	**<0.0001**	3.10 (2.77, 3.46)	**<0.0001**

†Model 1 adjusted for age and age-squared. ‡Model 2 additionally adjusted for smoking and alcohol consumption. §Model 3 additionally adjusted for physical activity and highest educational attainment. ¶In women, menopausal status was included as covariate.

Table S11: Odds ratios of metabolic syndrome (NCEP ATP III criteria) across increasing deciles of hemoglobin levels among European Whites

**Metabolic Syndrome**	**Cases (%)**	**Model 1**†	***P*-value**	**Model 2**‡	***P*-value**	**Model 3**§	***P*-value**
**Men (*n*=182,048)**	89,330 (49.0)						
D1 (<13.80 g/dL) vs. D2	8,178 (46.3)	1.04 (1.001, 1.09)	**0.0431**	1.03 (0.99, 1.08)	0.1622	0.99 (0.95, 1.04)	0.7693
D2 (13.80-14.24 g/dL)	8,173 (43.8)	Reference	-	Reference	-	Reference	-
D3 (14.25-14.54 g/dL) vs. D2	8,178 (44.5)	1.06 (1.02, 1.11)	**0.0041**	1.07 (1.03, 1.12)	**0.0012**	1.08 (1.03, 1.13)	**0.0014**
D4 (14.55-14.80 g/dL) vs. D2	9,022 (46.0)	1.13 (1.09, 1.18)	**<0.0001**	1.14 (1.10, 1.19)	**<0.0001**	1.13 (1.08, 1.19)	**<0.0001**
D5 (14.81-15.02 g/dL) vs. D2	7,954 (48.1)	1.26 (1.21, 1.32)	**<0.0001**	1.28 (1.23, 1.33)	**<0.0001**	1.27 (1.21, 1.33)	**<0.0001**
D6 (15.03-15.29 g/dL) vs. D2	8,899 (48.9)	1.31 (1.25, 1.36)	**<0.0001**	1.33 (1.27, 1.38)	**<0.0001**	1.30 (1.24, 1.36)	**<0.0001**
D7 (15.30-15.51 g/dL) vs. D2	9,055 (49.1)	1.33 (1.27, 1.38)	**<0.0001**	1.35 (1.29, 1.40)	**<0.0001**	1.33 (1.27, 1.39)	**<0.0001**
D8 (15.52-15.81 g/dL) vs. D2	9,438 (51.5)	1.47 (1.41, 1.53)	**<0.0001**	1.50 (1.44, 1.56)	**<0.0001**	1.47 (1.40, 1.53)	**<0.0001**
D9 (15.82-16.23 g/dL) vs. D2	9,748 (54.1)	1.65 (1.58, 1.72)	**<0.0001**	1.68 (1.61, 1.75)	**<0.0001**	1.63 (1.55, 1.70)	**<0.0001**
D10 (>16.23 g/dL) vs. D2	10,685 (58.4)	1.94 (1.87, 2.03)	**<0.0001**	1.98 (1.90, 2.06)	**<0.0001**	1.89 (1.80, 1.98)	**<0.0001**
**Women¶ (*n*=204,429)**	76,714 (37.5)						
D1 (<12.40 g/dL) vs. D2	6,124 (30.0)	1.07 (1.03, 1.12)	**0.0017**	1.04 (0.99, 1.09)	0.0899	1.00 (0.95, 1.05)	0.9514
D2 (12.40-12.79 g/dL)	5,871 (29.8)	Reference	-	Reference	-	Reference	-
D3 (12.80-13.09 g/dL) vs. D2	6,618 (31.1)	1.04 (0.997, 1.09)	0.0672	1.05 (1.001, 1.09)	**0.0454**	1.04 (0.99, 1.10)	0.1255
D4 (13.10-13.30 g/dL) vs. D2	6,937 (33.1)	1.12 (1.08, 1.17)	**<0.0001**	1.13 (1.08, 1.18)	**<0.0001**	1.13 (1.08, 1.19)	**<0.0001**
D5 (13.31-13.52 g/dL) vs. D2	6,872 (34.8)	1.20 (1.15, 1.25)	**<0.0001**	1.21 (1.16, 1.27)	**<0.0001**	1.20 (1.14, 1.27)	**<0.0001**
D6 (13.53-13.75 g/dL) vs. D2	7,474 (36.6)	1.29 (1.23, 1.34)	**<0.0001**	1.30 (1.25, 1.36)	**<0.0001**	1.30 (1.23, 1.36)	**<0.0001**
D7 (13.76-13.99 g/dL) vs. D2	7,873 (39.4)	1.44 (1.38, 1.50)	**<0.0001**	1.46 (1.40, 1.52)	**<0.0001**	1.45 (1.38, 1.52)	**<0.0001**
D8 (14.00-14.28 g/dL) vs. D2	8,901 (41.8)	1.58 (1.52, 1.65)	**<0.0001**	1.60 (1.54, 1.67)	**<0.0001**	1.60 (1.53, 1.68)	**<0.0001**
D9 (14.29-14.67 g/dL) vs. D2	9,281 (45.4)	1.79 (1.71, 1.87)	**<0.0001**	1.80 (1.73, 1.88)	**<0.0001**	1.81 (1.72, 1.90)	**<0.0001**
D10 (>14.67 g/dL) vs. D2	10,763 (53.0)	2.36 (2.26, 2.46)	**<0.0001**	2.34 (2.24, 2.44)	**<0.0001**	2.35 (2.24, 2.47)	**<0.0001**

†Model 1 adjusted for age and age-squared. ‡Model 2 additionally adjusted for smoking and alcohol consumption. §Model 3 additionally adjusted for physical activity and highest educational attainment. ¶In women, menopausal status was included as covariate.

Table S12: Odds ratios of central obesity across increasing deciles of hemoglobin levels among Taiwanese Han Chinese

Central Obesity	Cases (%)	Model 1†	*P*-value	Model 2‡	*P*-value	Model 3§	*P*-value
**Men (*n*=20,670)**	7,802 (37.8)						
D1 (<13.6 g/dL)	596 (29.8)	Reference	-	Reference	-	Reference	-
D2 (13.6-14.1 g/dL) vs. D1	637 (30.2)	1.02 (0.89, 1.17)	0.7600	1.03 (0.90, 1.18)	0.6875	1.12 (0.98, 1.29)	0.1081
D3 (14.2-14.5 g/dL) vs. D1	762 (33.9)	1.22 (1.07, 1.39)	**0.0031**	1.22 (1.07, 1.39)	**0.0029**	1.32 (1.15, 1.51)	**<0.0001**
D4 (14.6-14.8 g/dL) vs. D1	676 (33.4)	1.19 (1.05, 1.36)	**0.0093**	1.20 (1.05, 1.37)	**0.0087**	1.26 (1.10, 1.46)	**0.0011**
D5 (14.9-15.1 g/dL) vs. D1	812 (35.8)	1.33 (1.17, 1.51)	**<0.0001**	1.33 (1.17, 1.52)	**<0.0001**	1.38 (1.20, 1.58)	**<0.0001**
D6 (15.2-15.3 g/dL) vs. D1	594 (38.7)	1.51 (1.31, 1.74)	**<0.0001**	1.51 (1.31, 1.74)	**<0.0001**	1.57 (1.35, 1.82)	**<0.0001**
D7 (15.4-15.6 g/dL) vs. D1	870 (39.7)	1.58 (1.39, 1.80)	**<0.0001**	1.58 (1.39, 1.80)	**<0.0001**	1.58 (1.38, 1.81)	**<0.0001**
D8 (15.7-16.0 g/dL) vs. D1	1,011 (41.6)	1.71 (1.51, 1.94)	**<0.0001**	1.70 (1.50, 1.93)	**<0.0001**	1.68 (1.47, 1.92)	**<0.0001**
D9 (16.1-16.4 g/dL) vs. D1	777 (44.1)	1.90 (1.66, 2.18)	**<0.0001**	1.88 (1.64, 2.16)	**<0.0001**	1.78 (1.54, 2.05)	**<0.0001**
D10 (>16.4 g/dL) vs. D1	1,067 (50.8)	2.48 (2.18, 2.83)	**<0.0001**	2.40 (2.11, 2.74)	**<0.0001**	2.03 (1.77, 2.33)	**<0.0001**
**Women¶ (*n*=46,567)**	22,310 (47.9)						
D1 (<11.6 g/dL)	1,932 (40.2)	Reference	-	Reference	-	Reference	-
D2 (11.6-12.1 g/dL) vs. D1	1,661 (41.1)	0.96 (0.88, 1.05)	0.3621	0.96 (0.88, 1.05)	0.3691	1.01 (0.93, 1.11)	0.7629
D3 (12.2-12.5 g/dL) vs. D1	1,846 (41.4)	0.95 (0.87, 1.03)	0.1993	0.94 (0.87, 1.03)	0.1846	1.01 (0.92, 1.10)	0.8560
D4 (12.6-12.8 g/dL) vs. D1	1,979 (43.4)	1.01 (0.93, 1.10)	0.7723	1.01 (0.93, 1.10)	0.8125	1.06 (0.98, 1.16)	0.1608
D5 (12.9-13.1 g/dL) vs. D1	2,339 (44.9)	1.06 (0.98, 1.15)	0.1465	1.06 (0.98, 1.15)	0.1636	1.11 (1.02, 1.21)	**0.0179**
D6 (13.2-13.4 g/dL) vs. D1	2,737 (48.0)	1.20 (1.11, 1.30)	**<0.0001**	1.20 (1.11, 1.30)	**<0.0001**	1.22 (1.13, 1.33)	**<0.0001**
D7 (13.5-13.6 g/dL) vs. D1	1,705 (50.6)	1.33 (1.21, 1.45)	**<0.0001**	1.32 (1.21, 1.45)	**<0.0001**	1.32 (1.20, 1.45)	**<0.0001**
D8 (13.7-13.9 g/dL) vs. D1	2,403 (51.7)	1.36 (1.25, 1.48)	**<0.0001**	1.36 (1.25, 1.47)	**<0.0001**	1.33 (1.22, 1.45)	**<0.0001**
D9 (14.0-14.4 g/dL) vs. D1	3,074 (55.6)	1.59 (1.47, 1.72)	**<0.0001**	1.58 (1.46, 1.72)	**<0.0001**	1.49 (1.37, 1.62)	**<0.0001**
D10 (>14.4 g/dL) vs. D1	2,634 (62.1)	2.04 (1.87, 2.22)	**<0.0001**	2.03 (1.86, 2.22)	**<0.0001**	1.72 (1.57, 1.89)	**<0.0001**

†Model 1 adjusted for age and age-squared. ‡Model 2 additionally adjusted for smoking and alcohol consumption. §Model 3 additionally adjusted for physical activity, highest educational attainment, and comorbidities. ¶In women, menopausal status was included as covariate.

Table S13: Odds ratios of central obesity across increasing deciles of hemoglobin levels among European Whites

Central Obesity	Cases (%)	Model 1†	*P*-value	Model 2‡	*P*-value	Model 3§	*P*-value
**Men (*n*=182,048)**	101,772 (55.9)						
D1 (<13.80 g/dL)	8,552 (48.7)	Reference	-	Reference	-	Reference	-
D2 (13.80-14.24 g/dL) vs. D1	9,297 (48.9)	1.04 (1.00, 1.09)	**0.0444**	1.05 (1.01, 1.10)	**0.0173**	1.17 (1.11, 1.22)	**<0.0001**
D3 (14.25-14.54 g/dL) vs. D1	9,213 (51.0)	1.15 (1.10, 1.20)	**<0.0001**	1.17 (1.12, 1.22)	**<0.0001**	1.28 (1.22, 1.34)	**<0.0001**
D4 (14.55-14.80 g/dL) vs. D1	10,441 (53.2)	1.26 (1.21, 1.32)	**<0.0001**	1.29 (1.23, 1.34)	**<0.0001**	1.41 (1.34, 1.48)	**<0.0001**
D5 (14.81-15.02 g/dL) vs. D1	9,197 (55.6)	1.41 (1.35, 1.47)	**<0.0001**	1.44 (1.38, 1.51)	**<0.0001**	1.55 (1.47, 1.63)	**<0.0001**
D6 (15.03-15.29 g/dL) vs. D1	10,480 (56.2)	1.44 (1.38, 1.50)	**<0.0001**	1.48 (1.42, 1.54)	**<0.0001**	1.58 (1.50, 1.65)	**<0.0001**
D7 (15.30-15.51 g/dL) vs. D1	10,374 (57.8)	1.55 (1.49, 1.62)	**<0.0001**	1.60 (1.53, 1.66)	**<0.0001**	1.69 (1.61, 1.77)	**<0.0001**
D8 (15.52-15.81 g/dL) vs. D1	10,935 (59.7)	1.68 (1.61, 1.75)	**<0.0001**	1.74 (1.66, 1.81)	**<0.0001**	1.78 (1.69, 1.87)	**<0.0001**
D9 (15.82-16.23 g/dL) vs. D1	11,161 (61.9)	1.85 (1.77, 1.93)	**<0.0001**	1.92 (1.84, 2.00)	**<0.0001**	1.90 (1.81, 2.00)	**<0.0001**
D10 (>16.23 g/dL) vs. D1	12,122 (66.3)	2.22 (2.13, 2.32)	**<0.0001**	2.32 (2.22, 2.42)	**<0.0001**	2.21 (2.11, 2.33)	**<0.0001**
**Women¶ (*n*=204,429)**	117,475 (57.5)						
D1 (<12.40 g/dL)	9,811 (48.4)	Reference	-	Reference	-	Reference	-
D2 (12.40-12.79 g/dL) vs. D1	9,743 (49.4)	1.01 (0.97, 1.05)	0.6505	1.03 (0.99, 1.07)	0.1371	1.07 (1.02, 1.13)	**0.0033**
D3 (12.80-13.09 g/dL) vs. D1	11,073 (52.0)	1.11 (1.06, 1.15)	**<0.0001**	1.13 (1.09, 1.18)	**<0.0001**	1.17 (1.12, 1.23)	**<0.0001**
D4 (13.10-13.30 g/dL) vs. D1	11,212 (53.4)	1.16 (1.12, 1.21)	**<0.0001**	1.20 (1.15, 1.24)	**<0.0001**	1.21 (1.16, 1.27)	**<0.0001**
D5 (13.31-13.52 g/dL) vs. D1	10,982 (55.6)	1.26 (1.21, 1.31)	**<0.0001**	1.30 (1.24, 1.35)	**<0.0001**	1.30 (1.24, 1.36)	**<0.0001**
D6 (13.53-13.75 g/dL) vs. D1	11,715 (57.4)	1.35 (1.29, 1.40)	**<0.0001**	1.39 (1.34, 1.45)	**<0.0001**	1.40 (1.33, 1.47)	**<0.0001**
D7 (13.76-13.99 g/dL) vs. D1	12,002 (60.1)	1.49 (1.43, 1.55)	**<0.0001**	1.54 (1.48, 1.61)	**<0.0001**	1.50 (1.43, 1.57)	**<0.0001**
D8 (14.00-14.28 g/dL) vs. D1	13,223 (62.1)	1.61 (1.55, 1.68)	**<0.0001**	1.67 (1.62, 1.74)	**<0.0001**	1.60 (1.53, 1.68)	**<0.0001**
D9 (14.29-14.67 g/dL) vs. D1	13,394 (65.5)	1.84 (1.77, 1.92)	**<0.0001**	1.91 (1.83, 1.99)	**<0.0001**	1.76 (1.68, 1.85)	**<0.0001**
D10 (>14.67 g/dL) vs. D1	14,320 (70.5)	2.27 (2.18, 2.36)	**<0.0001**	2.35 (2.25, 2.45)	**<0.0001**	2.00 (1.91, 2.11)	**<0.0001**

†Model 1 adjusted for age and age-squared. ‡Model 2 additionally adjusted for smoking and alcohol consumption. §Model 3 additionally adjusted for physical activity, highest educational attainment, and comorbidities. ¶In women, menopausal status was included as covariate.

Table S14: Odds ratios of hypertension[††] across increasing deciles of hemoglobin levels among Taiwanese Han Chinese

**Hypertension**	**Cases (%)**	**Model 1[†]**	***P*-value**	**Model 2[‡]**	***P*-value**	**Model 3[§]**	***P*-value**
**Men (*n*=20,670)**	7,076 (34.2)						
D1 (<13.6 g/dL) vs. D2	712 (35.7)	1.07 (0.94, 1.23)	0.3115	1.07 (0.94, 1.23)	0.3060	1.03 (0.90, 1.19)	0.6604
D2 (13.6-14.1 g/dL)	679 (32.2)	Reference	-	Reference	-	Reference	-
D3 (14.2-14.5 g/dL) vs. D2	711 (31.6)	1.05 (0.92, 1.20)	0.4959	1.05 (0.92, 1.20)	0.4719	1.05 (0.91, 1.20)	0.5306
D4 (14.6-14.8 g/dL) vs. D2	658 (32.5)	1.15 (1.001, 1.32)	**0.0479**	1.15 (1.001, 1.32)	**0.0489**	1.14 (0.99, 1.32)	0.0620
D5 (14.9-15.1 g/dL) vs. D2	744 (32.8)	1.18 (1.04, 1.35)	**0.0131**	1.18 (1.03, 1.35)	**0.0150**	1.16 (1.01, 1.33)	**0.0348**
D6 (15.2-15.3 g/dL) vs. D2	497 (32.4)	1.24 (1.07, 1.44)	**0.0040**	1.24 (1.07, 1.44)	**0.0052**	1.23 (1.05, 1.43)	**0.0085**
D7 (15.4-15.6 g/dL) vs. D2	733 (33.4)	1.35 (1.18, 1.55)	**<0.0001**	1.35 (1.18, 1.55)	**<0.0001**	1.30 (1.13, 1.49)	**0.0002**
D8 (15.7-16.0 g/dL) vs. D2	844 (34.7)	1.46 (1.28, 1.67)	**<0.0001**	1.46 (1.28, 1.67)	**<0.0001**	1.40 (1.23, 1.60)	**<0.0001**
D9 (16.1-16.4 g/dL) vs. D2	609 (34.6)	1.58 (1.37, 1.82)	**<0.0001**	1.56 (1.35, 1.80)	**<0.0001**	1.46 (1.26, 1.69)	**<0.0001**
D10 (>16.4 g/dL) vs. D2	889 (42.4)	2.21 (1.94, 2.53)	**<0.0001**	2.19 (1.91, 2.51)	**<0.0001**	1.95 (1.70, 2.24)	**<0.0001**
**Women[¶] (*n*=46,567)**	9,002 (19.3)						
D1 (<11.6 g/dL) vs. D3	560 (11.7)	1.11 (0.98, 1.27)	0.0988	1.11 (0.98, 1.27)	0.1004	1.07 (0.94, 1.22)	0.2944
D2 (11.6-12.1 g/dL) vs. D3	573 (14.2)	1.06 (0.93, 1.21)	0.3640	1.06 (0.93, 1.21)	0.3596	1.06 (0.93, 1.21)	0.3669
D3 (12.2-12.5 g/dL)	647 (14.5)	Reference	-	Reference	-	Reference	-
D4 (12.6-12.8 g/dL) vs. D3	756 (16.6)	1.13 (1.002, 1.27)	**0.0468**	1.13 (1.002, 1.27)	**0.0475**	1.15 (1.02, 1.30)	**0.0272**
D5 (12.9-13.1 g/dL) vs. D3	901 (17.3)	1.14 (1.02, 1.28)	**0.0231**	1.14 (1.02, 1.28)	**0.0241**	1.15 (1.02, 1.30)	**0.0198**
D6 (13.2-13.4 g/dL) vs. D3	1,076 (18.9)	1.25 (1.12, 1.40)	**<0.0001**	1.25 (1.12, 1.40)	**<0.0001**	1.25 (1.12, 1.40)	**0.0001**
D7 (13.5-13.6 g/dL) vs. D3	695 (20.6)	1.40 (1.23, 1.58)	**<0.0001**	1.40 (1.23, 1.58)	**<0.0001**	1.37 (1.21, 1.56)	**<0.0001**
D8 (13.7-13.9 g/dL) vs. D3	1,066 (22.9)	1.53 (1.36, 1.71)	**<0.0001**	1.53 (1.36, 1.71)	**<0.0001**	1.50 (1.34, 1.69)	**<0.0001**
D9 (14.0-14.4 g/dL) vs. D3	1,378 (24.9)	1.71 (1.53, 1.90)	**<0.0001**	1.71 (1.53, 1.90)	**<0.0001**	1.63 (1.46, 1.82)	**<0.0001**
D10 (>14.4 g/dL) vs. D3	1,350 (31.8)	2.33 (2.09, 2.61)	**<0.0001**	2.34 (2.09, 2.62)	**<0.0001**	2.12 (1.89, 2.39)	**<0.0001**

[†]Model 1 adjusted for age and age-squared. [‡]Model 2 additionally adjusted for smoking and alcohol consumption. [§]Model 3 additionally adjusted for physical activity, highest educational attainment, and comorbidities. [¶]In women, menopausal status was included as covariate. [††]Based on Taiwan Society of Cardiology and Taiwan Hypertension Society classification of hypertension.

Table S15: Odds ratios of hypertension†† across increasing deciles of hemoglobin levels among European Whites

Hypertension	Cases (%)	Model 1†	*P*-value	Model 2‡	*P*-value	Model 3§	*P*-value
**Men (*n*=182,048)**	116,701 (64.1)						
D1 (<13.80 g/dL)	11,243 (64.1)	Reference	-	Reference	-	Reference	-
D2 (13.80-14.24 g/dL) vs. D1	11,461 (60.3)	0.94 (0.90, 0.98)	**0.0054**	0.94 (0.90, 0.99)	**0.0091**	1.01 (0.97, 1.07)	0.5851
D3 (14.25-14.54 g/dL) vs. D1	10,882 (60.2)	0.98 (0.93, 1.01)	0.2729	0.98 (0.94, 1.03)	0.4121	1.06 (1.01, 1.12)	**0.0152**
D4 (14.55-14.80 g/dL) vs. D1	12,026 (61.3)	1.04 (0.99, 1.08)	0.1225	1.04 (0.998, 1.09)	0.0595	1.11 (1.05, 1.16)	**<0.0001**
D5 (14.81-15.02 g/dL) vs. D1	10,249 (62.0)	1.11 (1.06, 1.16)	**<0.0001**	1.12 (1.07, 1.17)	**<0.0001**	1.20 (1.14, 1.26)	**<0.0001**
D6 (15.03-15.29 g/dL) vs. D1	11,898 (63.8)	1.20 (1.15, 1.25)	**<0.0001**	1.21 (1.16, 1.27)	**<0.0001**	1.28 (1.22, 1.34)	**<0.0001**
D7 (15.30-15.51 g/dL) vs. D1	11,345 (63.2)	1.20 (1.14, 1.25)	**<0.0001**	1.21 (1.16, 1.27)	**<0.0001**	1.27 (1.21, 1.34)	**<0.0001**
D8 (15.52-15.81 g/dL) vs. D1	11,977 (65.4)	1.33 (1.27, 1.39)	**<0.0001**	1.35 (1.29, 1.41)	**<0.0001**	1.40 (1.33, 1.48)	**<0.0001**
D9 (15.82-16.23 g/dL) vs. D1	12,287 (68.2)	1.53 (1.46, 1.60)	**<0.0001**	1.55 (1.48, 1.62)	**<0.0001**	1.61 (1.53, 1.69)	**<0.0001**
D10 (>16.23 g/dL) vs. D1	13,333 (72.9)	1.91 (1.82, 2.00)	**<0.0001**	1.95 (1.86, 2.04)	**<0.0001**	1.99 (1.89, 2.10)	**<0.0001**
**Women¶ (*n*=204,429)**	102,058 (49.9)						
D1 (<12.40 g/dL) vs. D2	8,365 (41.3)	1.07 (1.03, 1.12)	**0.0016**	1.06 (1.01, 1.10)	**0.0112**	1.02 (0.97, 1.07)	0.4327
D2 (12.40-12.79 g/dL)	8,228 (41.7)	Reference	-	Reference	-	Reference	-
D3 (12.80-13.09 g/dL) vs. D2	9,245 (43.4)	1.04 (0.998, 1.08)	0.0650	1.04 (0.999, 1.09)	0.0571	1.03 (0.98, 1.08)	0.2840
D4 (13.10-13.30 g/dL) vs. D2	9,570 (45.6)	1.12 (1.07, 1.17)	**<0.0001**	1.13 (1.08, 1.17)	**<0.0001**	1.12 (1.07, 1.17)	**<0.0001**
D5 (13.31-13.52 g/dL) vs. D2	9,406 (47.6)	1.18 (1.13, 1.23)	**<0.0001**	1.19 (1.14, 1.24)	**<0.0001**	1.19 (1.13, 1.25)	**<0.0001**
D6 (13.53-13.75 g/dL) vs. D2	10,002 (49.0)	1.24 (1.19, 1.29)	**<0.0001**	1.25 (1.20, 1.30)	**<0.0001**	1.24 (1.18, 1.30)	**<0.0001**
D7 (13.76-13.99 g/dL) vs. D2	10,264 (51.4)	1.34 (1.28, 1.40)	**<0.0001**	1.35 (1.30, 1.41)	**<0.0001**	1.33 (1.27, 1.40)	**<0.0001**
D8 (14.00-14.28 g/dL) vs. D2	11,573 (54.4)	1.50 (1.44, 1.56)	**<0.0001**	1.52 (1.45, 1.58)	**<0.0001**	1.49 (1.42, 1.56)	**<0.0001**
D9 (14.29-14.67 g/dL) vs. D2	12,018 (58.9)	1.75 (1.68, 1.82)	**<0.0001**	1.77 (1.70, 1.85)	**<0.0001**	1.70 (1.62, 1.78)	**<0.0001**
D10 (>14.67 g/dL) vs. D2	13,387 (65.9)	2.31 (2.21, 2.41)	**<0.0001**	2.35 (2.25, 2.45)	**<0.0001**	2.22 (2.11, 2.33)	**<0.0001**

†Model 1 adjusted for age and age-squared. ‡Model 2 additionally adjusted for smoking and alcohol consumption. §Model 3 additionally adjusted for physical activity, highest educational attainment, and comorbidities. ¶In women, menopausal status was included as covariate. ††Based on European Society of Cardiology/National Institute for Health and Care Excellence classification of hypertension.

Table S16: Odds ratios of dyslipidemia†† across increasing deciles of hemoglobin levels among Taiwanese Han Chinese

Dyslipidemia	Cases (%)	Model 1†	*P*-value	Model 2‡	*P*-value	Model 3§	*P*-value
**Men (*n*=20,670)**	9,066 (43.9)						
D1 (<13.6 g/dL) vs. D2	796 (39.9)	1.20 (1.06, 1.36)	**0.0053**	1.19 (1.04, 1.35)	**0.0087**	1.14 (1.002, 1.30)	**0.0458**
D2 (13.6-14.1 g/dL)	750 (35.6)	Reference	-	Reference	-	Reference	-
D3 (14.2-14.5 g/dL) vs. D2	860 (38.2)	1.13 (1.001, 1.28)	**0.0490**	1.12 (0.99, 1.27)	0.0764	1.12 (0.99, 1.27)	0.0792
D4 (14.6-14.8 g/dL) vs. D2	798 (39.4)	1.19 (1.05, 1.35)	**0.0070**	1.18 (1.04, 1.34)	**0.0123**	1.17 (1.03, 1.34)	**0.0159**
D5 (14.9-15.1 g/dL) vs. D2	966 (42.6)	1.37 (1.21, 1.55)	**<0.0001**	1.36 (1.20, 1.54)	**<0.0001**	1.35 (1.19, 1.53)	**<0.0001**
D6 (15.2-15.3 g/dL) vs. D2	659 (42.9)	1.39 (1.21, 1.59)	**<0.0001**	1.37 (1.19, 1.57)	**<0.0001**	1.36 (1.19, 1.57)	**<0.0001**
D7 (15.4-15.6 g/dL) vs. D2	994 (45.4)	1.55 (1.37, 1.75)	**<0.0001**	1.53 (1.35, 1.73)	**<0.0001**	1.50 (1.32, 1.70)	**<0.0001**
D8 (15.7-16.0 g/dL) vs. D2	1,128 (46.4)	1.61 (1.43, 1.82)	**<0.0001**	1.58 (1.40, 1.78)	**<0.0001**	1.53 (1.35, 1.73)	**<0.0001**
D9 (16.1-16.4 g/dL) vs. D2	894 (50.8)	1.94 (1.70, 2.21)	**<0.0001**	1.88 (1.65, 2.14)	**<0.0001**	1.81 (1.58, 2.06)	**<0.0001**
D10 (>16.4 g/dL) vs. D2	1,221 (58.2)	2.60 (2.29, 2.95)	**<0.0001**	2.44 (2.15, 2.77)	**<0.0001**	2.20 (1.93, 2.50)	**<0.0001**
**Women¶ (*n*=46,567)**	19,868 (42.7)						
D1 (<11.6 g/dL) vs. D2	1,796 (37.4)	1.16 (1.07, 1.27)	**0.0007**	1.16 (1.07, 1.27)	**0.0008**	1.15 (1.05, 1.25)	**0.0026**
D2 (11.6-12.1 g/dL)	1,459 (36.1)	Reference	-	Reference	-	Reference	-
D3 (12.2-12.5 g/dL) vs. D2	1,620 (36.3)	0.97 (0.89, 1.07)	0.5717	0.98 (0.89, 1.07)	0.5723	0.98 (0.90, 1.07)	0.6688
D4 (12.6-12.8 g/dL) vs. D2	1,753 (38.5)	1.05 (0.96, 1.15)	0.2776	1.05 (0.96, 1.15)	0.2851	1.05 (0.96, 1.15)	0.2513
D5 (12.9-13.1 g/dL) vs. D2	2,091 (40.1)	1.11 (1.02, 1.21)	**0.0177**	1.11 (1.02, 1.21)	**0.0177**	1.12 (1.03, 1.22)	**0.0107**
D6 (13.2-13.4 g/dL) vs. D2	2,421 (42.5)	1.21 (1.11, 1.32)	**<0.0001**	1.21 (1.11, 1.31)	**<0.0001**	1.21 (1.11, 1.31)	**<0.0001**
D7 (13.5-13.6 g/dL) vs. D2	1,483 (44.0)	1.29 (1.17, 1.41)	**<0.0001**	1.28 (1.17, 1.41)	**<0.0001**	1.26 (1.14, 1.38)	**<0.0001**
D8 (13.7-13.9 g/dL) vs. D2	2,132 (45.9)	1.36 (1.24, 1.48)	**<0.0001**	1.35 (1.24, 1.48)	**<0.0001**	1.33 (1.21, 1.45)	**<0.0001**
D9 (14.0-14.4 g/dL) vs. D2	2,749 (49.7)	1.57 (1.44, 1.71)	**<0.0001**	1.56 (1.44, 1.70)	**<0.0001**	1.50 (1.38, 1.64)	**<0.0001**
D10 (>14.4 g/dL) vs. D2	2,364 (55.8)	1.96 (1.79, 2.14)	**<0.0001**	1.93 (1.77, 2.12)	**<0.0001**	1.74 (1.59, 1.91)	**<0.0001**

†Model 1 adjusted for age and age-squared. ‡Model 2 additionally adjusted for smoking and alcohol consumption. §Model 3 additionally adjusted for physical activity, highest educational attainment, and comorbidities. ¶In women, menopausal status was included as covariate. ††Described as either hypertriglyceridemia, low levels of HLD-C, or a combination of the two.

Table S17: Odds ratios of dyslipidemia[††] across increasing deciles of hemoglobin levels among European Whites

Dyslipidemia	Cases (%)	Model 1[†]	*P*-value	Model 2[‡]	*P*-value	Model 3[§]	*P*-value
**Men (*n*=182,048)**	128,064 (70.4)						
D1 (<13.80 g/dL)	11,993 (68.4)	Reference	-	Reference	-	Reference	-
D2 (13.80-14.24 g/dL) vs. D1	12,662 (66.7)	0.97 (0.93, 1.01)	0.1420	0.98 (0.94, 1.02)	0.3563	1.07 (1.01, 1.12)	**0.0134**
D3 (14.25-14.54 g/dL) vs. D1	12,228 (67.7)	1.03 (0.99, 1.08)	0.1728	1.05 (1.00, 1.10)	**0.0327**	1.14 (1.09, 1.20)	**<0.0001**
D4 (14.55-14.80 g/dL) vs. D1	13,484 (68.7)	1.09 (1.04, 1.14)	**0.0002**	1.11 (1.06, 1.16)	**<0.0001**	1.20 (1.14, 1.26)	**<0.0001**
D5 (14.81-15.02 g/dL) vs. D1	11,486 (69.5)	1.14 (1.09, 1.20)	**<0.0001**	1.17 (1.11, 1.22)	**<0.0001**	1.25 (1.18, 1.31)	**<0.0001**
D6 (15.03-15.29 g/dL) vs. D1	13,137 (70.4)	1.20 (1.14, 1.25)	**<0.0001**	1.22 (1.17, 1.28)	**<0.0001**	1.29 (1.23, 1.36)	**<0.0001**
D7 (15.30-15.51 g/dL) vs. D1	12,764 (71.1)	1.25 (1.19, 1.31)	**<0.0001**	1.27 (1.21, 1.33)	**<0.0001**	1.36 (1.29, 1.43)	**<0.0001**
D8 (15.52-15.81 g/dL) vs. D1	13,302 (72.6)	1.35 (1.29, 1.41)	**<0.0001**	1.37 (1.31, 1.44)	**<0.0001**	1.45 (1.37, 1.52)	**<0.0001**
D9 (15.82-16.23 g/dL) vs. D1	13,159 (73.0)	1.38 (1.32, 1.44)	**<0.0001**	1.40 (1.34, 1.47)	**<0.0001**	1.43 (1.35, 1.50)	**<0.0001**
D10 (>16.23 g/dL) vs. D1	13,849 (75.7)	1.57 (1.50, 1.65)	**<0.0001**	1.58 (1.51, 1.66)	**<0.0001**	1.56 (1.48, 1.64)	**<0.0001**
**Women[¶] (*n*=204,429)**	138,861 (67.9)						
D1 (<12.40 g/dL)	12,085 (59.6)	Reference	-	Reference	-	Reference	-
D2 (12.40-12.79 g/dL) vs. D1	12,025 (61.0)	0.98 (0.94, 1.02)	0.3765	1.00 (0.96, 1.04)	0.9564	1.02 (0.97, 1.07)	0.3095
D3 (12.80-13.09 g/dL) vs. D1	13,473 (63.2)	1.06 (1.01, 1.10)	**0.0103**	1.08 (1.03, 1.12)	**0.0004**	1.11 (1.06, 1.16)	**<0.0001**
D4 (13.10-13.30 g/dL) vs. D1	13,713 (65.3)	1.14 (1.09, 1.19)	**<0.0001**	1.16 (1.12, 1.21)	**<0.0001**	1.21 (1.16, 1.27)	**<0.0001**
D5 (13.31-13.52 g/dL) vs. D1	13,086 (66.3)	1.16 (1.11, 1.21)	**<0.0001**	1.18 (1.13, 1.24)	**<0.0001**	1.19 (1.14, 1.25)	**<0.0001**
D6 (13.53-13.75 g/dL) vs. D1	13,933 (68.3)	1.26 (1.21, 1.32)	**<0.0001**	1.29 (1.24, 1.35)	**<0.0001**	1.30 (1.24, 1.36)	**<0.0001**
D7 (13.76-13.99 g/dL) vs. D1	14,010 (70.2)	1.35 (1.30, 1.41)	**<0.0001**	1.38 (1.32, 1.44)	**<0.0001**	1.38 (1.31, 1.45)	**<0.0001**
D8 (14.00-14.28 g/dL) vs. D1	15,329 (72.0)	1.47 (1.41, 1.53)	**<0.0001**	1.49 (1.43, 1.55)	**<0.0001**	1.49 (1.42, 1.57)	**<0.0001**
D9 (14.29-14.67 g/dL) vs. D1	15,184 (74.3)	1.60 (1.53, 1.67)	**<0.0001**	1.61 (1.54, 1.68)	**<0.0001**	1.56 (1.49, 1.64)	**<0.0001**
D10 (>14.67 g/dL) vs. D1	16,023 (78.9)	2.00 (1.91, 2.09)	**<0.0001**	1.96 (1.87, 2.05)	**<0.0001**	1.83 (1.74, 1.93)	**<0.0001**

[†]Model 1 adjusted for age and age-squared. [‡]Model 2 additionally adjusted for smoking and alcohol consumption. [§]Model 3 additionally adjusted for physical activity, highest educational attainment, and comorbidities. [¶]In women, menopausal status was included as covariate. [††]Described as either hypertriglyceridemia, low levels of HLD-C, or a combination of the two.

Table S18: Odds ratios of hypercholesterolemia†† across increasing deciles of hemoglobin levels among Taiwanese Han Chinese

**Hypercholesterolemia**	**Cases (%)**	**Model 1†**	***P*-value**	**Model 2‡**	***P*-value**	**Model 3§**	***P*-value**
**Men (*n*=20,670)**	4,359 (21.1)						
D1 (<13.6 g/dL)	343 (17.2)	Reference	-	Reference	-	Reference	-
D2 (13.6-14.1 g/dL) vs. D1	347 (16.5)	0.97 (0.82, 1.15)	0.7292	0.98 (0.83, 1.15)	0.7756	1.01 (0.85, 1.19)	0.9320
D3 (14.2-14.5 g/dL) vs. D1	427 (19.0)	1.18 (1.01, 1.38)	**0.0392**	1.18 (1.01, 1.39)	**0.0376**	1.22 (1.04, 1.44)	**0.0135**
D4 (14.6-14.8 g/dL) vs. D1	405 (20.0)	1.27 (1.08, 1.49)	**0.0034**	1.27 (1.09, 1.50)	**0.0032**	1.31 (1.12, 1.55)	**0.0010**
D5 (14.9-15.1 g/dL) vs. D1	467 (20.6)	1.33 (1.14, 1.56)	**0.0003**	1.33 (1.14, 1.56)	**0.0003**	1.37 (1.17, 1.61)	**<0.0001**
D6 (15.2-15.3 g/dL) vs. D1	327 (21.3)	1.41 (1.19, 1.67)	**<0.0001**	1.41 (1.19, 1.67)	**<0.0001**	1.45 (1.22, 1.72)	**<0.0001**
D7 (15.4-15.6 g/dL) vs. D1	496 (22.6)	1.55 (1.33, 1.81)	**<0.0001**	1.55 (1.33, 1.81)	**<0.0001**	1.57 (1.34, 1.84)	**<0.0001**
D8 (15.7-16.0 g/dL) vs. D1	542 (22.3)	1.52 (1.30, 1.77)	**<0.0001**	1.51 (1.30, 1.76)	**<0.0001**	1.52 (1.30, 1.77)	**<0.0001**
D9 (16.1-16.4 g/dL) vs. D1	418 (23.7)	1.69 (1.44, 1.99)	**<0.0001**	1.68 (1.43, 1.97)	**<0.0001**	1.67 (1.42, 1.97)	**<0.0001**
D10 (>16.4 g/dL) vs. D1	587 (28.0)	2.08 (1.79, 2.42)	**<0.0001**	2.04 (1.75, 2.38)	**<0.0001**	1.92 (1.65, 2.25)	**<0.0001**
**Women¶ (*n*=46,567)**	9,361 (20.1)						
D1 (<11.6 g/dL)	450 (9.4)	Reference	-	Reference	-	Reference	-
D2 (11.6-12.1 g/dL) vs. D1	548 (13.6)	1.26 (1.10, 1.44)	**0.0007**	1.26 (1.10, 1.44)	**0.0008**	1.28 (1.12, 1.47)	**0.0004**
D3 (12.2-12.5 g/dL) vs. D1	676 (15.1)	1.35 (1.18, 1.53)	**<0.0001**	1.35 (1.18, 1.53)	**<0.0001**	1.37 (1.20, 1.56)	**<0.0001**
D4 (12.6-12.8 g/dL) vs. D1	830 (18.2)	1.64 (1.44, 1.86)	**<0.0001**	1.64 (1.44, 1.85)	**<0.0001**	1.66 (1.46, 1.89)	**<0.0001**
D5 (12.9-13.1 g/dL) vs. D1	1,034 (19.8)	1.77 (1.57, 2.00)	**<0.0001**	1.77 (1.57, 2.00)	**<0.0001**	1.81 (1.60, 2.04)	**<0.0001**
D6 (13.2-13.4 g/dL) vs. D1	1,206 (21.2)	1.87 (1.66, 2.11)	**<0.0001**	1.87 (1.66, 2.11)	**<0.0001**	1.90 (1.68, 2.14)	**<0.0001**
D7 (13.5-13.6 g/dL) vs. D1	737 (21.9)	1.96 (1.72, 2.23)	**<0.0001**	1.95 (1.72, 2.23)	**<0.0001**	1.96 (1.72, 2.23)	**<0.0001**
D8 (13.7-13.9 g/dL) vs. D1	1,122 (24.1)	2.14 (1.89, 2.41)	**<0.0001**	2.13 (1.89, 2.41)	**<0.0001**	2.14 (1.89, 2.41)	**<0.0001**
D9 (14.0-14.4 g/dL) vs. D1	1,448 (26.2)	2.36 (2.10, 2.65)	**<0.0001**	2.35 (2.09, 2.64)	**<0.0001**	2.32 (2.06, 2.61)	**<0.0001**
D10 (>14.4 g/dL) vs. D1	1,310 (30.9)	2.85 (2.53, 3.22)	**<0.0001**	2.83 (2.51, 3.19)	**<0.0001**	2.67 (2.37, 3.02)	**<0.0001**

†Model 1 adjusted for age and age-squared. ‡Model 2 additionally adjusted for smoking and alcohol consumption. §Model 3 additionally adjusted for physical activity, highest educational attainment, and comorbidities. ¶In women, menopausal status was included as covariate. ††Described as either hypercholesterolemia, high levels of LDL-C, or a combination of the two.

Table S19: Odds ratios of hypercholesterolemia†† across increasing deciles of hemoglobin levels among European Whites

Hypercholesterolemia	Cases (%)	Model 1†	*P*-value	Model 2‡	*P*-value	Model 3§	*P*-value
**Men (*n*=182,048)**	93,741 (51.5)						
D1 (<13.80 g/dL) vs. D2	9,152 (52.2)	1.14 (1.09, 1.19)	**<0.0001**	1.12 (1.07, 1.17)	**<0.0001**	1.03 (0.98, 1.08)	0.2360
D2 (13.80-14.24 g/dL)	9,439 (49.7)	Reference	-	Reference	-	Reference	-
D3 (14.25-14.54 g/dL) vs. D2	9,068 (50.2)	1.10 (0.97, 1.05)	0.6845	1.02 (0.97, 1.06)	0.4765	1.02 (0.98, 1.07)	0.3642
D4 (14.55-14.80 g/dL) vs. D2	9,889 (50.4)	1.06 (1.02, 1.10)	**0.0052**	1.07 (1.02, 1.11)	**0.0019**	1.07 (1.02, 1.12)	**0.0045**
D5 (14.81-15.02 g/dL) vs. D2	8,403 (50.8)	1.08 (1.03, 1.12)	**0.0006**	1.09 (1.04, 1.14)	**0.0001**	1.08 (1.03, 1.13)	**0.0029**
D6 (15.03-15.29 g/dL) vs. D2	9,716 (52.1)	1.09 (1.05, 1.14)	**<0.0001**	1.10 (1.06, 1.15)	**<0.0001**	1.07 (1.03, 1.13)	**0.0026**
D7 (15.30-15.51 g/dL) vs. D2	9,240 (51.5)	1.13 (1.08, 1.18)	**<0.0001**	1.14 (1.09, 1.18)	**<0.0001**	1.10 (1.05, 1.16)	**<0.0001**
D8 (15.52-15.81 g/dL) vs. D2	9,586 (52.3)	1.20 (1.15, 1.25)	**<0.0001**	1.20 (1.15, 1.25)	**<0.0001**	1.17 (1.11, 1.22)	**<0.0001**
D9 (15.82-16.23 g/dL) vs. D2	9,470 (52.6)	1.26 (1.21, 1.31)	**<0.0001**	1.26 (1.21, 1.32)	**<0.0001**	1.18 (1.12, 1.23)	**<0.0001**
D10 (>16.23 g/dL) vs. D2	9,778 (53.5)	1.41 (1.35, 1.47)	**<0.0001**	1.39 (1.33, 1.45)	**<0.0001**	1.23 (1.18, 1.29)	**<0.0001**
**Women¶ (*n*=204,429)**	100,585 (49.2)						
D1 (<12.40 g/dL) vs. D2	7,622 (37.6)	1.13 (1.09, 1.18)	**<0.0001**	1.10 (1.06, 1.14)	**<0.0001**	1.04 (0.99, 1.09)	0.0958
D2 (12.40-12.79 g/dL)	8,153 (41.3)	Reference	-	Reference	-	Reference	-
D3 (12.80-13.09 g/dL) vs. D2	9,320 (43.7)	1.03 (0.99, 1.07)	0.1168	1.04 (0.996, 1.08)	0.0778	1.04 (0.99, 1.09)	0.0856
D4 (13.10-13.30 g/dL) vs. D2	9,874 (47.0)	1.04 (0.999, 1.08)	0.0534	1.04 (1.00, 1.09)	**0.0360**	1.05 (1.00, 1.10)	0.0524
D5 (13.31-13.52 g/dL) vs. D2	9,479 (48.0)	1.03 (0.99, 1.07)	0.1465	1.04 (0.995, 1.08)	0.0847	1.01 (0.96, 1.06)	0.7501
D6 (13.53-13.75 g/dL) vs. D2	10,261 (50.3)	1.07 (1.03, 1.11)	**0.0012**	1.07 (1.03, 1.12)	**0.0005**	1.05 (1.00, 1.10)	**0.0337**
D7 (13.76-13.99 g/dL) vs. D2	10,458 (52.4)	1.14 (1.10, 1.19)	**<0.0001**	1.15 (1.10, 1.19)	**<0.0001**	1.10 (1.05, 1.15)	**<0.0001**
D8 (14.00-14.28 g/dL) vs. D2	11,496 (54.0)	1.21 (1.16, 1.26)	**<0.0001**	1.20 (1.16, 1.25)	**<0.0001**	1.16 (1.10, 1.21)	**<0.0001**
D9 (14.29-14.67 g/dL) vs. D2	11,589 (56.7)	1.29 (1.24, 1.35)	**<0.0001**	1.28 (1.23, 1.33)	**<0.0001**	1.18 (1.13, 1.24)	**<0.0001**
D10 (>14.67 g/dL) vs. D2	12,333 (60.7)	1.60 (1.53, 1.66)	**<0.0001**	1.53 (1.47, 1.59)	**<0.0001**	1.35 (1.29, 1.42)	**<0.0001**

†Model 1 adjusted for age and age-squared. ‡Model 2 additionally adjusted for smoking and alcohol consumption. §Model 3 additionally adjusted for physical activity, highest educational attainment, and comorbidities. ¶In women, menopausal status was included as covariate. ††Described as either hypercholesterolemia, high levels of LDL-C, or a combination of the two.

Table S20: Odds ratios of gout across increasing deciles of hemoglobin levels among Taiwanese Han Chinese

Gout	Cases (%)	Model 1†	*P*-value	Model 2‡	*P*-value	Model 3§	*P*-value
**Men (*n*=20,670)**	6,876 (33.3)						
D1 (<13.6 g/dL) vs. D3	646 (32.3)	1.18 (1.03, 1.34)	**0.0156**	1.18 (1.03, 1.35)	**0.0139**	1.18 (1.03, 1.35)	**0.0181**
D2 (13.6-14.1 g/dL) vs. D3	619 (29.3)	1.02 (0.89, 1.16)	0.8008	1.02 (0.89, 1.16)	0.8152	1.04 (0.91, 1.19)	0.5898
D3 (14.2-14.5 g/dL)	655 (29.1)	Reference	-	Reference	-	Reference	-
D4 (14.6-14.8 g/dL) vs. D3	599 (29.6)	1.02 (0.90, 1.17)	0.7488	1.02 (0.90, 1.17)	0.7537	1.00 (0.88, 1.15)	0.9483
D5 (14.9-15.1 g/dL) vs. D3	754 (33.2)	1.21 (1.07, 1.37)	**0.0034**	1.21 (1.06, 1.37)	**0.0039**	1.17 (1.03, 1.33)	**0.0185**
D6 (15.2-15.3 g/dL) vs. D3	504 (32.8)	1.18 (1.03, 1.36)	**0.0196**	1.18 (1.02, 1.35)	**0.0250**	1.13 (0.98, 1.30)	0.1014
D7 (15.4-15.6 g/dL) vs. D3	760 (34.7)	1.28 (1.13, 1.45)	**0.0001**	1.27 (1.12, 1.45)	**0.0002**	1.20 (1.05, 1.36)	**0.0064**
D8 (15.7-16.0 g/dL) vs. D3	854 (35.1)	1.30 (1.15, 1.47)	**<0.0001**	1.30 (1.15, 1.47)	**<0.0001**	1.20 (1.06, 1.37)	**0.0043**
D9 (16.1-16.4 g/dL) vs. D3	645 (36.6)	1.38 (1.21, 1.58)	**<0.0001**	1.37 (1.20, 1.56)	**<0.0001**	1.23 (1.07, 1.40)	**0.0038**
D10 (>16.4 g/dL) vs. D3	841 (40.1)	1.61 (1.42, 1.83)	**<0.0001**	1.58 (1.39, 1.80)	**<0.0001**	1.33 (1.17, 1.51)	**<0.0001**
**Women¶ (*n*=46,567)**	6,704 (14.4)						
D1 (<11.6 g/dL)	396 (8.3)	Reference	-	Reference	-	Reference	-
D2 (11.6-12.1 g/dL) vs. D1	428 (10.6)	1.16 (1.00, 1.34)	**0.0499**	1.16 (1.00, 1.34)	**0.0470**	1.21 (1.05, 1.41)	**0.0107**
D3 (12.2-12.5 g/dL) vs. D1	483 (10.8)	1.14 (0.99, 1.31)	0.0681	1.14 (0.99, 1.31)	0.0701	1.21 (1.05, 1.40)	**0.0090**
D4 (12.6-12.8 g/dL) vs. D1	503 (11.0)	1.13 (0.99, 1.30)	0.0801	1.13 (0.98, 1.30)	0.0955	1.18 (1.02, 1.36)	**0.0244**
D5 (12.9-13.1 g/dL) vs. D1	658 (12.6)	1.30 (1.14, 1.49)	**0.0001**	1.30 (1.14, 1.48)	**0.0001**	1.35 (1.17, 1.54)	**<0.0001**
D6 (13.2-13.4 g/dL) vs. D1	808 (14.2)	1.47 (1.29, 1.68)	**<0.0001**	1.47 (1.29, 1.67)	**<0.0001**	1.49 (1.31, 1.70)	**<0.0001**
D7 (13.5-13.6 g/dL) vs. D1	554 (16.5)	1.75 (1.53, 2.02)	**<0.0001**	1.75 (1.52, 2.01)	**<0.0001**	1.74 (1.51, 2.00)	**<0.0001**
D8 (13.7-13.9 g/dL) vs. D1	773 (16.6)	1.73 (1.52, 1.97)	**<0.0001**	1.72 (1.51, 1.97)	**<0.0001**	1.68 (1.47, 1.92)	**<0.0001**
D9 (14.0-14.4 g/dL) vs. D1	1,029 (18.6)	1.97 (1.74, 2.24)	**<0.0001**	1.96 (1.73, 2.23)	**<0.0001**	1.83 (1.61, 2.08)	**<0.0001**
D10 (>14.4 g/dL) vs. D1	1,072 (25.3)	2.86 (2.52, 3.24)	**<0.0001**	2.84 (2.50, 3.22)	**<0.0001**	2.44 (2.15, 2.78)	**<0.0001**

†Model 1 adjusted for age and age-squared. ‡Model 2 additionally adjusted for smoking and alcohol consumption. §Model 3 additionally adjusted for physical activity, highest educational attainment, and comorbidities. ¶In women, menopausal status was included as covariate.

Table S21: Odds ratios of gout across increasing deciles of hemoglobin levels among European Whites

**Gout**	**Cases (%)**	**Model 1**†	***P*-value**	**Model 2**‡	***P*-value**	**Model 3**§	***P*-value**
**Men (*n*=182,048)**	36,401 (20.0)						
D1 (<13.80 g/dL) vs. D3	3,549 (20.2)	1.12 (1.07, 1.18)	**<0.0001**	1.14 (1.08, 1.20)	**<0.0001**	1.11 (1.04, 1.17)	**0.0010**
D2 (13.80-14.24 g/dL) vs. D3	3,474 (18.3)	1.01 (0.96, 1.06)	0.7324	1.01 (0.96, 1.06)	0.7989	1.01 (0.95, 1.07)	0.7908
D3 (14.25-14.54 g/dL)	3,261 (18.0)	Reference	-	Reference	-	Reference	-
D4 (14.55-14.80 g/dL) vs. D3	3,693 (18.8)	1.05 (1.001, 1.11)	**0.0465**	1.05 (0.999, 1.11)	0.0525	1.03 (0.97, 1.09)	0.4090
D5 (14.81-15.02 g/dL) vs. D3	3,193 (19.3)	1.10 (1.04, 1.16)	**0.0010**	1.10 (1.05, 1.17)	**0.0004**	1.08 (1.02, 1.15)	**0.0125**
D6 (15.03-15.29 g/dL) vs. D3	3,615 (20.1)	1.13 (1.08, 1.19)	**<0.0001**	1.14 (1.08, 1.21)	**<0.0001**	1.09 (1.03, 1.16)	**0.0025**
D7 (15.30-15.51 g/dL) vs. D3	3,843 (21.0)	1.16 (1.10, 1.22)	**<0.0001**	1.17 (1.11, 1.23)	**<0.0001**	1.12 (1.06, 1.19)	**0.0001**
D8 (15.52-15.81 g/dL) vs. D3	3,843 (21.0)	1.22 (1.16, 1.28)	**<0.0001**	1.24 (1.18, 1.31)	**<0.0001**	1.16 (1.09, 1.23)	**<0.0001**
D9 (15.82-16.23 g/dL) vs. D3	3,789 (21.0)	1.22 (1.16, 1.29)	**<0.0001**	1.25 (1.19, 1.32)	**<0.0001**	1.16 (1.10, 1.23)	**<0.0001**
D10 (>16.23 g/dL) vs. D3	4,280 (23.4)	1.40 (1.33, 1.47)	**<0.0001**	1.46 (1.39, 1.54)	**<0.0001**	1.29 (1.22, 1.37)	**<0.0001**
**Women¶ (*n*=204,429)**	19,305 (9.4)						
D1 (<12.40 g/dL) vs. D3	1,567 (7.7)	1.23 (1.14, 1.32)	**<0.0001**	1.21 (1.12, 1.30)	**<0.0001**	1.12 (1.03, 1.23)	**0.0113**
D2 (12.40-12.79 g/dL) vs. D3	1,330 (6.7)	1.00 (0.92, 1.08)	0.9165	0.99 (0.92, 1.07)	0.8654	1.01 (0.92, 1.11)	0.8324
D3 (12.80-13.09 g/dL)	1,471 (6.9)	Reference	-	Reference	-	Reference	-
D4 (13.10-13.30 g/dL) vs. D3	1,664 (7.9)	1.14 (1.06, 1.23)	**0.0003**	1.15 (1.07, 1.23)	**0.0003**	1.17 (1.07, 1.27)	**0.0005**
D5 (13.31-13.52 g/dL) vs. D3	1,642 (8.3)	1.18 (1.10, 1.27)	**<0.0001**	1.19 (1.10, 1.28)	**<0.0001**	1.17 (1.07, 1.27)	**0.0005**
D6 (13.53-13.75 g/dL) vs. D3	1,823 (8.9)	1.27 (1.18, 1.36)	**<0.0001**	1.28 (1.19, 1.38)	**<0.0001**	1.25 (1.15, 1.36)	**<0.0001**
D7 (13.76-13.99 g/dL) vs. D3	1,933 (9.7)	1.36 (1.27, 1.46)	**<0.0001**	1.38 (1.28, 1.48)	**<0.0001**	1.30 (1.19, 1.41)	**<0.0001**
D8 (14.00-14.28 g/dL) vs. D3	2,271 (10.7)	1.51 (1.40, 1.61)	**<0.0001**	1.52 (1.42, 1.63)	**<0.0001**	1.41 (1.30, 1.53)	**<0.0001**
D9 (14.29-14.67 g/dL) vs. D3	2,490 (12.2)	1.71 (1.60, 1.83)	**<0.0001**	1.73 (1.62, 1.85)	**<0.0001**	1.53 (1.41, 1.66)	**<0.0001**
D10 (>14.67 g/dL) vs. D3	3,114 (15.3)	2.17 (2.03, 2.31)	**<0.0001**	2.21 (2.07, 2.36)	**<0.0001**	1.84 (1.70, 1.99)	**<0.0001**

†Model 1 adjusted for age and age-squared. ‡Model 2 additionally adjusted for smoking and alcohol consumption. §Model 3 additionally adjusted for physical activity, highest educational attainment, and comorbidities. ¶In women, menopausal status was included as covariate.

Table S22: Odds ratios of diabetes across increasing deciles of hemoglobin levels among Taiwanese Han Chinese

Diabetes	Cases (%)	Model 1†	*P*-value	Model 2‡	*P*-value	Model 3§	*P*-value
**Men (*n*=20,670)**	2,810 (13.6)						
D1 (<13.6 g/dL) vs. D6	386 (19.3)	1.59 (1.30, 1.95)	**<0.0001**	1.59 (1.30, 1.95)	**<0.0001**	1.71 (1.39, 2.10)	**<0.0001**
D2 (13.6-14.1 g/dL) vs. D6	307 (14.6)	1.21 (0.98, 1.49)	0.0710	1.23 (1.00, 1.51)	0.0547	1.36 (1.10, 1.68)	**0.0047**
D3 (14.2-14.5 g/dL) vs. D6	295 (13.1)	1.14 (0.93, 1.41)	0.2118	1.15 (0.93, 1.41)	0.2004	1.23 (0.99, 1.52)	0.0602
D4 (14.6-14.8 g/dL) vs. D6	239 (11.8)	1.05 (0.85, 1.30)	0.6597	1.05 (0.84, 1.30)	0.6795	1.09 (0.87, 1.36)	0.4485
D5 (14.9-15.1 g/dL) vs. D6	277 (12.2)	1.11 (0.90, 1.37)	0.3462	1.11 (0.90, 1.37)	0.3326	1.14 (0.92, 1.41)	0.2476
D6 (15.2-15.3 g/dL)	163 (10.6)	Reference	-	Reference	-	Reference	-
D7 (15.4-15.6 g/dL) vs. D6	259 (11.8)	1.16 (0.94, 1.43)	0.1793	1.16 (0.93, 1.43)	0.1851	1.13 (0.91, 1.40)	0.2850
D8 (15.7-16.0 g/dL) vs. D6	298 (12.3)	1.23 (1.00, 1.52)	**0.0492**	1.22 (0.99, 1.50)	0.0616	1.18 (0.96, 1.46)	0.1197
D9 (16.1-16.4 g/dL) vs. D6	224 (12.7)	1.38 (1.11, 1.72)	**0.0043**	1.35 (1.08, 1.69)	**0.0076**	1.28 (1.02, 1.60)	**0.0341**
D10 (>16.4 g/dL) vs. D6	362 (17.3)	1.96 (1.60, 2.41)	**<0.0001**	1.89 (1.54, 2.32)	**<0.0001**	1.64 (1.33, 2.02)	**<0.0001**
**Women¶ (*n*=46,567)**	4,065 (8.7)						
D1 (<11.6 g/dL) vs. D5	301 (6.3)	1.34 (1.14, 1.58)	**0.0005**	1.34 (1.14, 1.57)	**0.0005**	1.32 (1.12, 1.56)	**0.0010**
D2 (11.6-12.1 g/dL) vs. D5	287 (7.1)	1.18 (1.01, 1.39)	**0.0431**	1.18 (1.00, 1.39)	**0.0461**	1.22 (1.03, 1.44)	**0.0218**
D3 (12.2-12.5 g/dL) vs. D5	319 (7.2)	1.10 (0.94, 1.29)	0.2261	1.10 (0.94, 1.29)	0.2332	1.16 (0.98, 1.36)	0.0806
D4 (12.6-12.8 g/dL) vs. D5	328 (7.2)	1.05 (0.90, 1.23)	0.5092	1.06 (0.90, 1.24)	0.5043	1.08 (0.92, 1.27)	0.3524
D5 (12.9-13.1 g/dL)	368 (7.1)	Reference	-	Reference	-	Reference	-
D6 (13.2-13.4 g/dL) vs. D5	452 (7.9)	1.11 (0.96, 1.29)	0.1509	1.11 (0.96, 1.29)	0.1491	1.10 (0.95, 1.28)	0.2130
D7 (13.5-13.6 g/dL) vs. D5	312 (9.3)	1.31 (1.12, 1.54)	**0.0009**	1.31 (1.12, 1.54)	**0.0009**	1.26 (1.07, 1.49)	**0.0054**
D8 (13.7-13.9 g/dL) vs. D5	422 (9.1)	1.22 (1.05, 1.42)	**0.0078**	1.22 (1.05, 1.42)	**0.0088**	1.13 (0.97, 1.31)	0.1201
D9 (14.0-14.4 g/dL) vs. D5	605 (10.9)	1.50 (1.31, 1.73)	**<0.0001**	1.50 (1.31, 1.72)	**<0.0001**	1.34 (1.16, 1.54)	**<0.0001**
D10 (>14.4 g/dL) vs. D5	671 (15.8)	2.22 (1.94, 2.55)	**<0.0001**	2.21 (1.93, 2.54)	**<0.0001**	1.83 (1.59, 2.10)	**<0.0001**

†Model 1 adjusted for age and age-squared. ‡Model 2 additionally adjusted for smoking and alcohol consumption. §Model 3 additionally adjusted for physical activity, highest educational attainment, and comorbidities. ¶In women, menopausal status was included as covariate.

Table S23: Odds ratios of diabetes across increasing deciles of hemoglobin levels among European Whites

**Diabetes**	**Cases (%)**	**Model 1**†	***P*-value**	**Model 2**‡	***P*-value**	**Model 3**§	***P*-value**
**Men (*n*=182,048)**	17,180 (9.4)						
D1 (<13.80 g/dL) vs. D8	2,854 (16.3)	1.94 (1.82, 2.08)	**<0.0001**	1.86 (1.74, 1.99)	**<0.0001**	2.10 (1.94, 2.27)	**<0.0001**
D2 (13.80-14.24 g/dL) vs. D8	1,888 (9.9)	1.17 (1.09, 1.25)	**<0.0001**	1.15 (1.07, 1.23)	**0.0002**	1.32 (1.21, 1.43)	**<0.0001**
D3 (14.25-14.54 g/dL) vs. D8	1,570 (8.7)	1.03 (0.96, 1.11)	0.4328	1.03 (0.96, 1.11)	0.3955	1.18 (1.08, 1.29)	**0.0001**
D4 (14.55-14.80 g/dL) vs. D8	1,647 (8.4)	1.01 (0.93, 1.08)	0.9648	1.00 (0.93, 1.08)	0.9916	1.14 (1.05, 1.24)	**0.0024**
D5 (14.81-15.02 g/dL) vs. D8	1,410 (8.5)	1.04 (0.96, 1.12)	0.3543	1.04 (0.96, 1.12)	0.3296	1.14 (1.05, 1.25)	**0.0028**
D6 (15.03-15.29 g/dL) vs. D8	1,556 (8.3)	1.01 (0.94, 1.09)	0.8010	1.02 (0.94, 1.10)	0.6908	1.09 (1.01, 1.19)	**0.0384**
D7 (15.30-15.51 g/dL) vs. D8	1,448 (8.1)	0.99 (0.92, 1.07)	0.7728	0.99 (0.91, 1.06)	0.7045	1.04 (0.95, 1.13)	0.4350
D8 (15.52-15.81 g/dL)	1,493 (8.2)	Reference	-	Reference	-	Reference	-
D9 (15.82-16.23 g/dL) vs. D8	1,533 (8.5)	1.05 (0.97, 1.13)	0.2128	1.04 (0.97, 1.13)	0.2604	1.03 (0.94, 1.12)	0.5500
D10 (>16.23 g/dL) vs. D8	1,781 (9.7)	1.20 (1.12, 1.29)	**<0.0001**	1.17 (1.09, 1.26)	**<0.0001**	1.07 (0.99, 1.17)	0.1041
**Women¶ (*n*=204,429)**	10,570 (5.2)						
D1 (<12.40 g/dL) vs. D8	1,400 (6.9)	1.70 (1.57, 1.85)	**<0.0001**	1.59 (1.46, 1.74)	**<0.0001**	1.81 (1.63, 2.01)	**<0.0001**
D2 (12.40-12.79 g/dL) vs. D8	913 (4.6)	1.07 (0.97, 1.17)	0.1823	1.06 (0.96, 1.16)	0.2534	1.21 (1.08, 1.35)	**0.0009**
D3 (12.80-13.09 g/dL) vs. D8	961 (4.5)	1.01 (0.93, 1.11)	0.7694	1.02 (0.93, 1.11)	0.7388	1.18 (1.06, 1.32)	**0.0033**
D4 (13.10-13.30 g/dL) vs. D8	909 (4.3)	0.96 (0.88, 1.06)	0.4333	0.97 (0.88, 1.06)	0.4726	1.06 (0.95, 1.19)	0.2913
D5 (13.31-13.52 g/dL) vs. D8	871 (4.4)	0.96 (0.88, 1.06)	0.3992	0.97 (0.88, 1.06)	0.4974	1.10 (0.99, 1.23)	0.0851
D6 (13.53-13.75 g/dL) vs. D8	898 (4.4)	0.95 (0.87, 1.05)	0.3181	0.97 (0.88, 1.06)	0.4707	1.03 (0.92, 1.15)	0.5818
D7 (13.76-13.99 g/dL) vs. D8	905 (4.5)	0.97 (0.88, 1.06)	0.4959	0.97 (0.89, 1.07)	0.5677	1.03 (0.92, 1.15)	0.6152
D8 (14.00-14.28 g/dL)	1,004 (4.7)	Reference	-	Reference	-	Reference	-
D9 (14.29-14.67 g/dL) vs. D8	1,161 (5.7)	1.19 (1.09, 1.30)	**<0.0001**	1.19 (1.09, 1.29)	**0.0001**	1.12 (1.01, 1.24)	**0.0371**
D10 (>14.67 g/dL) vs. D8	1,548 (7.6)	1.58 (1.46, 1.72)	**<0.0001**	1.53 (1.41, 1.66)	**<0.0001**	1.39 (1.26, 1.54)	**<0.0001**

†Model 1 adjusted for age and age-squared. ‡Model 2 additionally adjusted for smoking and alcohol consumption. §Model 3 additionally adjusted for physical activity, highest educational attainment, and comorbidities. ¶In women, menopausal status was included as covariate.

Table S24.1: Odds ratios of hypertension†† across increasing deciles of hemoglobin levels among Taiwanese Han Chinese

**Hypertension**	**Cases (%)**	**Model 1†**	***P*-value**	**Model 2‡**	***P*-value**	**Model 3§**	***P*-value**
**Men (*n*=20,670)**	7,076 (34.2)						
D1 (<13.6 g/dL) vs. D2	712 (35.7)	1.10 (0.95, 1.26)	0.1973	1.10 (0.96, 1.27)	0.1865	1.07 (0.93, 1.24)	0.3461
D2 (13.6-14.1 g/dL)	679 (32.2)	Reference	-	Reference	-	Reference	-
D3 (14.2-14.5 g/dL) vs. D2	711 (31.6)	1.01 (0.88, 1.17)	0.8479	1.02 (0.89, 1.17)	0.7954	1.02 (0.89, 1.17)	0.7993
D4 (14.6-14.8 g/dL) vs. D2	658 (32.5)	1.11 (0.96, 1.28)	0.1655	1.11 (0.96, 1.28)	0.1654	1.11 (0.96, 1.28)	0.1601
D5 (14.9-15.1 g/dL) vs. D2	744 (32.8)	1.09 (0.95, 1.25)	0.2397	1.09 (0.94, 1.25)	0.2481	1.08 (0.94, 1.24)	0.2728
D6 (15.2-15.3 g/dL) vs. D2	497 (32.4)	1.13 (0.97, 1.32)	0.1279	1.12 (0.96, 1.31)	0.1381	1.13 (0.97, 1.32)	0.1210
D7 (15.4-15.6 g/dL) vs. D2	733 (33.4)	1.18 (1.03, 1.36)	**0.0193**	1.18 (1.03, 1.36)	**0.0216**	1.17 (1.01, 1.35)	**0.0317**
D8 (15.7-16.0 g/dL) vs. D2	844 (34.7)	1.28 (1.11, 1.46)	**0.0005**	1.28 (1.12, 1.47)	**0.0004**	1.26 (1.10, 1.45)	**0.0010**
D9 (16.1-16.4 g/dL) vs. D2	609 (34.6)	1.34 (1.15, 1.55)	**0.0001**	1.33 (1.15, 1.55)	**0.0002**	1.29 (1.12, 1.50)	**0.0007**
D10 (>16.4 g/dL) vs. D2	889 (42.4)	1.76 (1.53, 2.03)	**<0.0001**	1.76 (1.53, 2.02)	**<0.0001**	1.67 (1.45, 1.92)	**<0.0001**
**Women¶ (*n*=46,567)**	9,002 (19.3)						
D1 (<11.6 g/dL) vs. D3	560 (11.7)	1.10 (0.96, 1.25)	0.1690	1.10 (0.96, 1.25)	0.1731	1.08 (0.94, 1.23)	0.2836
D2 (11.6-12.1 g/dL) vs. D3	573 (14.2)	1.07 (0.93, 1.22)	0.3469	1.07 (0.93, 1.22)	0.3449	1.07 (0.93, 1.22)	0.3485
D3 (12.2-12.5 g/dL)	647 (14.5)	Reference	-	Reference	-	Reference	-
D4 (12.6-12.8 g/dL) vs. D3	756 (16.6)	1.12 (0.99, 1.26)	0.0819	1.12 (0.99, 1.26)	0.0833	1.14 (1.003, 1.29)	**0.0447**
D5 (12.9-13.1 g/dL) vs. D3	901 (17.3)	1.11 (0.99, 1.25)	0.0885	1.11 (0.98, 1.25)	0.0922	1.12 (1.00, 1.27)	0.0613
D6 (13.2-13.4 g/dL) vs. D3	1,076 (18.9)	1.17 (1.04, 1.31)	**0.0078**	1.17 (1.04, 1.31)	**0.0079**	1.19 (1.06, 1.33)	**0.0043**
D7 (13.5-13.6 g/dL) vs. D3	695 (20.6)	1.28 (1.13, 1.46)	**0.0001**	1.28 (1.13, 1.46)	**0.0001**	1.29 (1.13, 1.46)	**0.0001**
D8 (13.7-13.9 g/dL) vs. D3	1,066 (22.9)	1.39 (1.24, 1.57)	**<0.0001**	1.39 (1.24, 1.57)	**<0.0001**	1.40 (1.24, 1.58)	**<0.0001**
D9 (14.0-14.4 g/dL) vs. D3	1,378 (24.9)	1.48 (1.33, 1.66)	**<0.0001**	1.48 (1.33, 1.66)	**<0.0001**	1.47 (1.31, 1.65)	**<0.0001**
D10 (>14.4 g/dL) vs. D3	1,350 (31.8)	1.92 (1.71, 2.15)	**<0.0001**	1.92 (1.71, 2.16)	**<0.0001**	1.85 (1.65, 2.08)	**<0.0001**

*With BMI as additional covariate in all models. †Model 1 adjusted for age and age-squared. ‡Model 2 additionally adjusted for smoking and alcohol consumption. §Model 3 additionally adjusted for physical activity, highest educational attainment, and comorbidities. ¶In women, menopausal status was included as covariate. ††Based on Taiwan Society of Cardiology and Taiwan Hypertension Society classification of hypertension.

Table S24.2: Odds ratios of hypertension†† across increasing deciles of hemoglobin levels among European Whites

**Hypertension**	**Cases (%)**	**Model 1†**	***P*-value**	**Model 2‡**	***P*-value**	**Model 3§**	***P*-value**
**Men (*n*=182,048)**	116,701 (64.1)						
D1 (<13.80 g/dL)	11,243 (64.1)	Reference	-	Reference	-	Reference	-
D2 (13.80-14.24 g/dL) vs. D1	11,461 (60.3)	0.92 (0.88, 0.97)	**0.0007**	0.93 (0.88, 0.97)	**0.0009**	0.98 (0.93, 1.03)	0.4767
D3 (14.25-14.54 g/dL) vs. D1	10,882 (60.2)	0.94 (0.90, 0.98)	**0.0072**	0.94 (0.90, 0.99)	**0.0109**	1.01 (0.96, 1.06)	0.6941
D4 (14.55-14.80 g/dL) vs. D1	12,026 (61.3)	0.97 (0.93, 1.02)	0.2251	0.98 (0.93, 1.02)	0.3049	1.03 (0.98, 1.08)	0.2393
D5 (14.81-15.02 g/dL) vs. D1	10,249 (62.0)	1.02 (0.97, 1.07)	0.3793	1.03 (0.98, 1.08)	0.2526	1.10 (1.04, 1.16)	**0.0004**
D6 (15.03-15.29 g/dL) vs. D1	11,898 (63.8)	1.10 (1.05, 1.15)	**<0.0001**	1.11 (1.06, 1.16)	**<0.0001**	1.17 (1.11, 1.23)	**<0.0001**
D7 (15.30-15.51 g/dL) vs. D1	11,345 (63.2)	1.08 (1.03, 1.13)	**0.0012**	1.09 (1.04, 1.14)	**0.0006**	1.15 (1.09, 1.21)	**<0.0001**
D8 (15.52-15.81 g/dL) vs. D1	11,977 (65.4)	1.17 (1.12, 1.23)	**<0.0001**	1.18 (1.13, 1.24)	**<0.0001**	1.25 (1.19, 1.32)	**<0.0001**
D9 (15.82-16.23 g/dL) vs. D1	12,287 (68.2)	1.33 (1.27, 1.40)	**<0.0001**	1.34 (1.28, 1.41)	**<0.0001**	1.42 (1.34, 1.49)	**<0.0001**
D10 (>16.23 g/dL) vs. D1	13,333 (72.9)	1.61 (1.53, 1.69)	**<0.0001**	1.62 (1.54, 1.70)	**<0.0001**	1.71 (1.62, 1.80)	**<0.0001**
**Women¶ (*n*=204,429)**	102,058 (49.9)						
D1 (<12.40 g/dL) vs. D2	8,365 (41.3)	1.07 (1.02, 1.12)	**0.0028**	1.06 (1.02, 1.11)	**0.0053**	1.04 (0.99, 1.09)	0.1518
D2 (12.40-12.79 g/dL)	8,228 (41.7)	Reference	-	Reference	-	Reference	-
D3 (12.80-13.09 g/dL) vs. D2	9,245 (43.4)	1.02 (0.98, 1.07)	0.2871	1.02 (0.98, 1.07)	0.2737	1.02 (0.97, 1.07)	0.5629
D4 (13.10-13.30 g/dL) vs. D2	9,570 (45.6)	1.09 (1.04, 1.14)	**<0.0001**	1.09 (1.05, 1.14)	**<0.0001**	1.09 (1.04, 1.15)	**0.0003**
D5 (13.31-13.52 g/dL) vs. D2	9,406 (47.6)	1.13 (1.09, 1.18)	**<0.0001**	1.14 (1.09, 1.19)	**<0.0001**	1.15 (1.09, 1.20)	**<0.0001**
D6 (13.53-13.75 g/dL) vs. D2	10,002 (49.0)	1.18 (1.13, 1.23)	**<0.0001**	1.19 (1.14, 1.24)	**<0.0001**	1.19 (1.13, 1.25)	**<0.0001**
D7 (13.76-13.99 g/dL) vs. D2	10,264 (51.4)	1.25 (1.19, 1.30)	**<0.0001**	1.25 (1.20, 1.31)	**<0.0001**	1.26 (1.20, 1.32)	**<0.0001**
D8 (14.00-14.28 g/dL) vs. D2	11,573 (54.4)	1.37 (1.32, 1.43)	**<0.0001**	1.38 (1.33, 1.44)	**<0.0001**	1.38 (1.32, 1.45)	**<0.0001**
D9 (14.29-14.67 g/dL) vs. D2	12,018 (58.9)	1.57 (1.50, 1.64)	**<0.0001**	1.58 (1.52, 1.65)	**<0.0001**	1.56 (1.48, 1.64)	**<0.0001**
D10 (>14.67 g/dL) vs. D2	13,387 (65.9)	2.00 (1.92, 2.09)	**<0.0001**	2.03 (1.94, 2.12)	**<0.0001**	1.99 (1.89, 2.10)	**<0.0001**

*With BMI as additional covariate in all models. †Model 1 adjusted for age and age-squared. ‡Model 2 additionally adjusted for smoking and alcohol consumption. §Model 3 additionally adjusted for physical activity, highest educational attainment, and comorbidities. ¶In women, menopausal status was included as covariate. ††Based on European Society of Cardiology/National Institute for Health and Care Excellence classification of hypertension.

Table S24.3: Odds ratios of dyslipidemia†† across increasing deciles of hemoglobin levels among Taiwanese Han Chinese

**Dyslipidemia**	**Cases (%)**	**Model 1†**	***P*-value**	**Model 2‡**	***P*-value**	**Model 3§**	***P*-value**
**Men (*n*=20,670)**	9,066 (43.9)						
D1 (<13.6 g/dL) vs. D2	796 (39.9)	1.22 (1.07, 1.39)	**0.0024**	1.21 (1.07, 1.38)	**0.0037**	1.18 (1.03, 1.34)	**0.0162**
D2 (13.6-14.1 g/dL)	750 (35.6)	Reference	-	Reference	-	Reference	-
D3 (14.2-14.5 g/dL) vs. D2	860 (38.2)	1.11 (0.97, 1.26)	0.1190	1.09 (0.96, 1.24)	0.1695	1.10 (0.97, 1.25)	0.1532
D4 (14.6-14.8 g/dL) vs. D2	798 (39.4)	1.15 (1.01, 1.31)	**0.0321**	1.14 (1.00, 1.30)	0.0503	1.14 (1.003, 1.31)	**0.0454**
D5 (14.9-15.1 g/dL) vs. D2	966 (42.6)	1.28 (1.13, 1.45)	**0.0001**	1.27 (1.12, 1.44)	**0.0003**	1.27 (1.12, 1.45)	**0.0002**
D6 (15.2-15.3 g/dL) vs. D2	659 (42.9)	1.28 (1.11, 1.47)	**0.0006**	1.26 (1.09, 1.45)	**0.0013**	1.27 (1.11, 1.47)	**0.0008**
D7 (15.4-15.6 g/dL) vs. D2	994 (45.4)	1.39 (1.22, 1.58)	**<0.0001**	1.37 (1.21, 1.56)	**<0.0001**	1.37 (1.20, 1.56)	**<0.0001**
D8 (15.7-16.0 g/dL) vs. D2	1,128 (46.4)	1.44 (1.27, 1.63)	**<0.0001**	1.41 (1.25, 1.60)	**<0.0001**	1.40 (1.23, 1.59)	**<0.0001**
D9 (16.1-16.4 g/dL) vs. D2	894 (50.8)	1.71 (1.49, 1.95)	**<0.0001**	1.65 (1.44, 1.89)	**<0.0001**	1.63 (1.43, 1.87)	**<0.0001**
D10 (>16.4 g/dL) vs. D2	1,221 (58.2)	2.14 (1.88, 2.43)	**<0.0001**	2.01 (1.76, 2.29)	**<0.0001**	1.91 (1.68, 2.18)	**<0.0001**
**Women¶ (*n*=46,567)**	19,868 (42.7)						
D1 (<11.6 g/dL) vs. D2	1,796 (37.4)	1.14 (1.04, 1.25)	**0.0046**	1.14 (1.04, 1.25)	**0.0047**	1.13 (1.03, 1.24)	**0.0080**
D2 (11.6-12.1 g/dL)	1,459 (36.1)	Reference	-	Reference	-	Reference	-
D3 (12.2-12.5 g/dL) vs. D2	1,620 (36.3)	0.97 (0.88, 1.06)	0.4954	0.97 (0.88, 1.06)	0.5000	0.97 (0.89, 1.07)	0.5628
D4 (12.6-12.8 g/dL) vs. D2	1,753 (38.5)	1.03 (0.94, 1.13)	0.5028	1.03 (0.94, 1.13)	0.5099	1.04 (0.94, 1.14)	0.4646
D5 (12.9-13.1 g/dL) vs. D2	2,091 (40.1)	1.08 (0.99, 1.18)	0.0879	1.08 (0.99, 1.18)	0.0859	1.09 (0.998, 1.19)	0.0549
D6 (13.2-13.4 g/dL) vs. D2	2,421 (42.5)	1.13 (1.04, 1.24)	**0.0049**	1.13 (1.04, 1.23)	**0.0055**	1.14 (1.04, 1.24)	**0.0038**
D7 (13.5-13.6 g/dL) vs. D2	1,483 (44.0)	1.18 (1.07, 1.30)	**0.0010**	1.18 (1.07, 1.30)	**0.0012**	1.17 (1.06, 1.29)	**0.0019**
D8 (13.7-13.9 g/dL) vs. D2	2,132 (45.9)	1.24 (1.14, 1.36)	**<0.0001**	1.24 (1.13, 1.36)	**<0.0001**	1.24 (1.13, 1.35)	**<0.0001**
D9 (14.0-14.4 g/dL) vs. D2	2,749 (49.7)	1.38 (1.26, 1.50)	**<0.0001**	1.37 (1.26, 1.50)	**<0.0001**	1.35 (1.24, 1.48)	**<0.0001**
D10 (>14.4 g/dL) vs. D2	2,364 (55.8)	1.61 (1.47, 1.77)	**<0.0001**	1.59 (1.45, 1.75)	**<0.0001**	1.51 (1.37, 1.66)	**<0.0001**

*With BMI as additional covariate in all models. †Model 1 adjusted for age and age-squared. ‡Model 2 additionally adjusted for smoking and alcohol consumption. §Model 3 additionally adjusted for physical activity, highest educational attainment, and comorbidities. ¶In women, menopausal status was included as covariate. ††Described as either hypertriglyceridemia, low levels of HLD-C, or a combination of the two.

Table S24.4: Odds ratios of dyslipidemia†† across increasing deciles of hemoglobin levels among European Whites

Dyslipidemia	Cases (%)	Model 1†	*P*-value	Model 2‡	*P*-value	Model 3§	*P*-value
**Men (*n*=182,048)**	128,064 (70.4)						
D1 (<13.80 g/dL)	11,993 (68.4)	Reference	-	Reference	-	Reference	-
D2 (13.80-14.24 g/dL) vs. D1	12,662 (66.7)	0.95 (0.91, 0.99)	**0.0260**	0.96 (0.92, 1.01)	0.0774	1.03 (0.98, 1.08)	0.3136
D3 (14.25-14.54 g/dL) vs. D1	12,228 (67.7)	0.99 (0.94, 1.04)	0.6074	1.00 (0.96, 1.05)	0.9019	1.08 (1.02, 1.13)	**0.0054**
D4 (14.55-14.80 g/dL) vs. D1	13,484 (68.7)	1.02 (0.97, 1.06)	0.4676	1.03 (0.99, 1.08)	0.1893	1.11 (1.06, 1.17)	**<0.0001**
D5 (14.81-15.02 g/dL) vs. D1	11,486 (69.5)	1.05 (1.001, 1.10)	**0.0433**	1.07 (1.02, 1.12)	**0.0086**	1.14 (1.08, 1.20)	**<0.0001**
D6 (15.03-15.29 g/dL) vs. D1	13,137 (70.4)	1.09 (1.04, 1.14)	**0.0002**	1.11 (1.06, 1.16)	**<0.0001**	1.17 (1.11, 1.23)	**<0.0001**
D7 (15.30-15.51 g/dL) vs. D1	12,764 (71.1)	1.12 (1.07, 1.18)	**<0.0001**	1.13 (1.08, 1.19)	**<0.0001**	1.21 (1.15, 1.28)	**<0.0001**
D8 (15.52-15.81 g/dL) vs. D1	13,302 (72.6)	1.18 (1.13, 1.24)	**<0.0001**	1.20 (1.14, 1.25)	**<0.0001**	1.27 (1.21, 1.34)	**<0.0001**
D9 (15.82-16.23 g/dL) vs. D1	13,159 (73.0)	1.19 (1.14, 1.25)	**<0.0001**	1.20 (1.14, 1.26)	**<0.0001**	1.25 (1.18, 1.31)	**<0.0001**
D10 (>16.23 g/dL) vs. D1	13,849 (75.7)	1.30 (1.24, 1.37)	**<0.0001**	1.29 (1.23, 1.36)	**<0.0001**	1.32 (1.25, 1.40)	**<0.0001**
**Women¶ (*n*=204,429)**	138,861 (67.9)						
D1 (<12.40 g/dL)	12,085 (59.6)	Reference	-	Reference	-	Reference	-
D2 (12.40-12.79 g/dL) vs. D1	12,025 (61.0)	0.98 (0.94, 1.02)	0.2764	0.99 (0.95, 1.03)	0.6292	1.00 (0.95, 1.05)	0.9456
D3 (12.80-13.09 g/dL) vs. D1	13,473 (63.2)	1.04 (0.995, 1.08)	0.0848	1.05 (1.01, 1.10)	**0.0180**	1.08 (1.03, 1.13)	**0.0024**
D4 (13.10-13.30 g/dL) vs. D1	13,713 (65.3)	1.11 (1.06, 1.16)	**<0.0001**	1.12 (1.08, 1.17)	**<0.0001**	1.17 (1.11, 1.22)	**<0.0001**
D5 (13.31-13.52 g/dL) vs. D1	13,086 (66.3)	1.11 (1.06, 1.16)	**<0.0001**	1.13 (1.08, 1.18)	**<0.0001**	1.14 (1.08, 1.20)	**<0.0001**
D6 (13.53-13.75 g/dL) vs. D1	13,933 (68.3)	1.20 (1.15, 1.25)	**<0.0001**	1.22 (1.17, 1.27)	**<0.0001**	1.22 (1.16, 1.28)	**<0.0001**
D7 (13.76-13.99 g/dL) vs. D1	14,010 (70.2)	1.26 (1.21, 1.32)	**<0.0001**	1.27 (1.22, 1.33)	**<0.0001**	1.29 (1.22, 1.35)	**<0.0001**
D8 (14.00-14.28 g/dL) vs. D1	15,329 (72.0)	1.35 (1.29, 1.41)	**<0.0001**	1.35 (1.30, 1.42)	**<0.0001**	1.38 (1.31, 1.45)	**<0.0001**
D9 (14.29-14.67 g/dL) vs. D1	15,184 (74.3)	1.44 (1.37, 1.50)	**<0.0001**	1.43 (1.37, 1.49)	**<0.0001**	1.42 (1.35, 1.50)	**<0.0001**
D10 (>14.67 g/dL) vs. D1	16,023 (78.9)	1.74 (1.66, 1.82)	**<0.0001**	1.69 (1.61, 1.77)	**<0.0001**	1.64 (1.56, 1.73)	**<0.0001**

*With BMI as additional covariate in all models. †Model 1 adjusted for age and age-squared. ‡Model 2 additionally adjusted for smoking and alcohol consumption. §Model 3 additionally adjusted for physical activity, highest educational attainment, and comorbidities. ¶In women, menopausal status was included as covariate. ††Described as either hypertriglyceridemia, low levels of HLD-C, or a combination of the two.

Table S24.5: Odds ratios of gout across increasing deciles of hemoglobin levels among Taiwanese Han Chinese

Gout	Cases (%)	Model 1†	*P*-value	Model 2‡	*P*-value	Model 3§	*P*-value
**Men (*n*=20,670)**	6,876 (33.3)						
D1 (<13.6 g/dL) vs. D3	646 (32.3)	1.24 (1.09, 1.42)	**0.0016**	1.24 (1.09, 1.42)	**0.0015**	1.24 (1.08, 1.42)	**0.0020**
D2 (13.6-14.1 g/dL) vs. D3	619 (29.3)	1.05 (0.92, 1.20)	0.4556	1.05 (0.92, 1.20)	0.4786	1.06 (0.93, 1.22)	0.3847
D3 (14.2-14.5 g/dL)	655 (29.1)	Reference	-	Reference	-	Reference	-
D4 (14.6-14.8 g/dL) vs. D3	599 (29.6)	1.01 (0.88, 1.16)	0.8779	1.01 (0.88, 1.16)	0.8929	1.00 (0.87, 1.15)	0.9890
D5 (14.9-15.1 g/dL) vs. D3	754 (33.2)	1.15 (1.01, 1.31)	**0.0356**	1.15 (1.01, 1.31)	**0.0400**	1.13 (0.99, 1.29)	0.0717
D6 (15.2-15.3 g/dL) vs. D3	504 (32.8)	1.11 (0.96, 1.28)	0.1727	1.10 (0.95, 1.27)	0.1920	1.08 (0.93, 1.25)	0.3109
D7 (15.4-15.6 g/dL) vs. D3	760 (34.7)	1.17 (1.02, 1.33)	**0.0205**	1.16 (1.02, 1.32)	**0.0260**	1.13 (0.99, 1.29)	0.0770
D8 (15.7-16.0 g/dL) vs. D3	854 (35.1)	1.18 (1.04, 1.34)	**0.0117**	1.18 (1.04, 1.34)	**0.0123**	1.13 (1.00, 1.29)	0.0592
D9 (16.1-16.4 g/dL) vs. D3	645 (36.6)	1.22 (1.07, 1.40)	**0.0041**	1.21 (1.06, 1.39)	**0.0064**	1.14 (0.99, 1.31)	0.0664
D10 (>16.4 g/dL) vs. D3	841 (40.1)	1.32 (1.16, 1.51)	**<0.0001**	1.31 (1.15, 1.49)	**<0.0001**	1.20 (1.05, 1.37)	**0.0083**
**Women¶ (*n*=46,567)**	6,704 (14.4)						
D1 (<11.6 g/dL)	396 (8.3)	Reference	-	Reference	-	Reference	-
D2 (11.6-12.1 g/dL) vs. D1	428 (10.6)	1.20 (1.04, 1.39)	**0.0159**	1.21 (1.04, 1.40)	**0.0141**	1.23 (1.05, 1.42)	**0.0083**
D3 (12.2-12.5 g/dL) vs. D1	483 (10.8)	1.18 (1.02, 1.37)	**0.0236**	1.18 (1.02, 1.37)	**0.0235**	1.22 (1.05, 1.41)	**0.0087**
D4 (12.6-12.8 g/dL) vs. D1	503 (11.0)	1.15 (0.99, 1.32)	0.0638	1.14 (0.99, 1.32)	0.0746	1.17 (1.01, 1.36)	**0.0321**
D5 (12.9-13.1 g/dL) vs. D1	658 (12.6)	1.31 (1.14, 1.50)	**0.0001**	1.30 (1.13, 1.49)	**0.0002**	1.33 (1.15, 1.52)	**<0.0001**
D6 (13.2-13.4 g/dL) vs. D1	808 (14.2)	1.39 (1.22, 1.59)	**<0.0001**	1.39 (1.22, 1.59)	**<0.0001**	1.41 (1.23, 1.61)	**<0.0001**
D7 (13.5-13.6 g/dL) vs. D1	554 (16.5)	1.63 (1.41, 1.88)	**<0.0001**	1.63 (1.41, 1.88)	**<0.0001**	1.64 (1.41, 1.89)	**<0.0001**
D8 (13.7-13.9 g/dL) vs. D1	773 (16.6)	1.59 (1.39, 1.82)	**<0.0001**	1.58 (1.38, 1.81)	**<0.0001**	1.57 (1.37, 1.79)	**<0.0001**
D9 (14.0-14.4 g/dL) vs. D1	1,029 (18.6)	1.69 (1.49, 1.93)	**<0.0001**	1.69 (1.48, 1.92)	**<0.0001**	1.64 (1.44, 1.87)	**<0.0001**
D10 (>14.4 g/dL) vs. D1	1,072 (25.3)	2.28 (2.00, 2.60)	**<0.0001**	2.27 (1.99, 2.59)	**<0.0001**	2.13 (1.87, 2.43)	**<0.0001**

*With BMI as additional covariate in all models. †Model 1 adjusted for age and age-squared. ‡Model 2 additionally adjusted for smoking and alcohol consumption. §Model 3 additionally adjusted for physical activity, highest educational attainment, and comorbidities. ¶In women, menopausal status was included as covariate.

Table S24.6: Odds ratios of gout across increasing deciles of hemoglobin levels among European Whites

Gout	Cases (%)	Model 1†	*P*-value	Model 2‡	*P*-value	Model 3§	*P*-value
**Men (*n*=182,048)**	36,401 (20.0)						
D1 (<13.80 g/dL) vs. D3	3,549 (20.2)	1.17 (1.11, 1.24)	**<0.0001**	1.19 (1.13, 1.26)	**<0.0001**	1.18 (1.11, 1.25)	**<0.0001**
D2 (13.80-14.24 g/dL) vs. D3	3,474 (18.3)	1.04 (0.98, 1.10)	0.2015	1.03 (0.98, 1.09)	0.2357	1.03 (0.97, 1.10)	0.2890
D3 (14.25-14.54 g/dL)	3,261 (18.0)	Reference	-	Reference	-	Reference	-
D4 (14.55-14.80 g/dL) vs. D3	3,693 (18.8)	1.02 (0.97, 1.08)	0.3807	1.02 (0.97, 1.08)	0.4018	1.01 (0.95, 1.07)	0.8702
D5 (14.81-15.02 g/dL) vs. D3	3,193 (19.3)	1.04 (0.99, 1.10)	0.1381	1.05 (0.99, 1.11)	0.0864	1.05 (0.98, 1.11)	0.1591
D6 (15.03-15.29 g/dL) vs. D3	3,615 (20.1)	1.07 (1.02, 1.13)	**0.0121**	1.08 (1.02, 1.14)	**0.0062**	1.05 (0.99, 1.12)	0.0927
D7 (15.30-15.51 g/dL) vs. D3	3,843 (21.0)	1.07 (1.02, 1.13)	**0.0120**	1.08 (1.03, 1.14)	**0.0040**	1.06 (1.001, 1.13)	**0.0449**
D8 (15.52-15.81 g/dL) vs. D3	3,843 (21.0)	1.10 (1.04, 1.16)	**0.0005**	1.12 (1.06, 1.18)	**<0.0001**	1.08 (1.02, 1.15)	**0.0101**
D9 (15.82-16.23 g/dL) vs. D3	3,789 (21.0)	1.08 (1.02, 1.14)	**0.0050**	1.10 (1.05, 1.17)	**0.0003**	1.07 (1.01, 1.14)	**0.0239**
D10 (>16.24 g/dL) vs. D3	4,280 (23.4)	1.18 (1.12, 1.24)	**<0.0001**	1.22 (1.16, 1.29)	**<0.0001**	1.16 (1.09, 1.23)	**<0.0001**
**Women¶ (*n*=204,429)**	19,305 (9.4)						
D1 (<12.40 g/dL) vs. D3	1,567 (7.7)	1.24 (1.15, 1.34)	**<0.0001**	1.25 (1.15, 1.35)	**<0.0001**	1.17 (1.07, 1.28)	**0.0009**
D2 (12.40-12.79 g/dL) vs. D3	1,330 (6.7)	1.02 (0.94, 1.11)	0.5981	1.02 (0.94, 1.11)	0.5978	1.03 (0.94, 1.13)	0.5686
D3 (12.80-13.09 g/dL) vs. D3	1,471 (6.9)	Reference	-	Reference	-	Reference	-
D4 (13.10-13.30 g/dL) vs. D3	1,664 (7.9)	1.12 (1.04, 1.21)	**0.0039**	1.12 (1.04, 1.21)	**0.0043**	1.14 (1.05, 1.25)	**0.0032**
D5 (13.31-13.52 g/dL) vs. D3	1,642 (8.3)	1.13 (1.04, 1.22)	**0.0024**	1.13 (1.04, 1.22)	**0.0024**	1.13 (1.03, 1.23)	**0.0095**
D6 (13.53-13.75 g/dL) vs. D3	1,823 (8.9)	1.20 (1.11, 1.29)	**<0.0001**	1.20 (1.11, 1.29)	**<0.0001**	1.19 (1.09, 1.30)	**<0.0001**
D7 (13.76-13.99 g/dL) vs. D3	1,933 (9.7)	1.22 (1.15, 1.33)	**<0.0001**	1.23 (1.15, 1.33)	**<0.0001**	1.21 (1.11, 1.32)	**<0.0001**
D8 (14.00-14.28 g/dL) vs. D3	2,271 (10.7)	1.33 (1.24, 1.43)	**<0.0001**	1.33 (1.24, 1.43)	**<0.0001**	1.29 (1.18, 1.40)	**<0.0001**
D9 (14.29-14.67 g/dL) vs. D3	2,490 (12.2)	1.45 (1.35, 1.55)	**<0.0001**	1.45 (1.35, 1.55)	**<0.0001**	1.37 (1.26, 1.49)	**<0.0001**
D10 (>14.67 g/dL) vs. D3	3,114 (15.3)	1.71 (1.60, 1.83)	**<0.0001**	1.72 (1.61, 1.84)	**<0.0001**	1.60 (1.47, 1.73)	**<0.0001**

*With BMI as additional covariate in all models. †Model 1 adjusted for age and age-squared. ‡Model 2 additionally adjusted for smoking and alcohol consumption. §Model 3 additionally adjusted for physical activity, highest educational attainment, and comorbidities. ¶In women, menopausal status was included as covariate.

Table S24.7: Odds ratios of diabetes across increasing deciles of hemoglobin levels among Taiwanese Han Chinese

**Diabetes**	**Cases (%)**	**Model 1†**	***P*-value**	**Model 2‡**	***P*-value**	**Model 3§**	***P*-value**
**Men (*n*=20,670)**	2,812 (13.6)						
D1 (<13.6 g/dL) vs. D6	388 (19.2)	1.80 (1.46, 2.21)	**<0.0001**	1.79 (1.46, 2.21)	**<0.0001**	1.83 (1.49, 2.26)	**<0.0001**
D2 (13.6-14.1 g/dL) vs. D6	307 (14.6)	1.33 (1.07, 1.64)	**0.0088**	1.34 (1.08, 1.66)	**0.0068**	1.42 (1.14, 1.76)	**0.0015**
D3 (14.2-14.5 g/dL) vs. D6	295 (13.1)	1.22 (0.99, 1.51)	0.0605	1.23 (0.99, 1.52)	0.0565	1.27 (1.03, 1.58)	**0.0277**
D4 (14.6-14.8 g/dL) vs. D6	239 (11.8)	1.11 (0.89, 1.38)	0.3614	1.10 (0.89, 1.37)	0.3839	1.12 (0.90, 1.40)	0.3125
D5 (14.9-15.1 g/dL) vs. D6	277 (12.2)	1.12 (0.91, 1.39)	0.2814	1.13 (0.91, 1.40)	0.2629	1.14 (0.92, 1.42)	0.2310
D6 (15.2-15.3 g/dL)	163 (10.6)	Reference	-	Reference	-	Reference	-
D7 (15.4-15.6 g/dL) vs. D6	259 (11.8)	1.13 (0.91, 1.40)	0.2859	1.13 (0.91, 1.40)	0.2879	1.11 (0.89, 1.38)	0.3484
D8 (15.7-16.0 g/dL) vs. D6	298 (12.3)	1.19 (0.97, 1.47)	0.1015	1.19 (0.96, 1.47)	0.1126	1.16 (0.94, 1.44)	0.1702
D9 (16.1-16.4 g/dL) vs. D6	224 (12.7)	1.32 (1.05, 1.65)	**0.0164**	1.30 (1.04, 1.62)	**0.0238**	1.25 (1.00, 1.57)	0.0544
D10 (>16.4 g/dL) vs. D6	362 (17.3)	1.78 (1.44, 2.18)	**<0.0001**	1.72 (1.40, 2.12)	**<0.0001**	1.57 (1.27, 1.94)	**<0.0001**
**Women¶ (*n*=46,567)**	4,065 (8.7)						
D1 (<11.6 g/dL) vs. D5	301 (6.3)	1.38 (1.17, 1.63)	**0.0001**	1.38 (1.17, 1.63)	**0.0001**	1.36 (1.15, 1.61)	**0.0004**
D2 (11.6-12.1 g/dL) vs. D5	287 (7.1)	1.24 (1.05, 1.46)	**0.0130**	1.23 (1.04, 1.46)	**0.0144**	1.24 (1.05, 1.47)	**0.0135**
D3 (12.2-12.5 g/dL) vs. D5	319 (7.2)	1.15 (0.98, 1.35)	0.0920	1.15 (0.98, 1.35)	0.0908	1.18 (0.999, 1.39)	0.0517
D4 (12.6-12.8 g/dL) vs. D5	328 (7.2)	1.08 (0.92, 1.27)	0.3547	1.08 (0.92, 1.27)	0.3494	1.10 (0.93, 1.29)	0.2596
D5 (12.9-13.1 g/dL)	368 (7.1)	Reference	-	Reference	-	Reference	-
D6 (13.2-13.4 g/dL) vs. D5	452 (7.9)	1.07 (0.92, 1.24)	0.4089	1.06 (0.92, 1.24)	0.4101	1.07 (0.92, 1.24)	0.3976
D7 (13.5-13.6 g/dL) vs. D5	312 (9.3)	1.23 (1.05, 1.45)	**0.0120**	1.23 (1.05, 1.45)	**0.0128**	1.22 (1.03, 1.44)	**0.0194**
D8 (13.7-13.9 g/dL) vs. D5	422 (9.1)	1.13 (0.97, 1.31)	0.1248	1.12 (0.97, 1.31)	0.1326	1.08 (0.93, 1.26)	0.3231
D9 (14.0-14.4 g/dL) vs. D5	605 (10.9)	1.32 (1.14, 1.51)	**0.0001**	1.32 (1.14, 1.52)	**0.0001**	1.25 (1.08, 1.44)	**0.0027**
D10 (>14.4 g/dL) vs. D5	671 (15.8)	1.82 (1.58, 2.10)	**<0.0001**	1.81 (1.58, 2.09)	**<0.0001**	1.64 (1.42, 1.89)	**<0.0001**

*With BMI as additional covariate in all models. †Model 1 adjusted for age and age-squared. ‡Model 2 additionally adjusted for smoking and alcohol consumption. §Model 3 additionally adjusted for physical activity, highest educational attainment, and comorbidities. ¶In women, menopausal status was included as covariate.

Table S24.8: Odds ratios of diabetes across increasing deciles of hemoglobin levels among European Whites

Diabetes	Cases (%)	Model 1†	*P*-value	Model 2‡	*P*-value	Model 3§	*P*-value
**Men (*n*=182,048)**	17,180 (9.4)						
D1 (<13.80 g/dL) vs. D8	2,854 (16.3)	2.33 (2.17, 2.50)	**<0.0001**	2.25 (2.10, 2.41)	**<0.0001**	2.33 (2.15, 2.53)	**<0.0001**
D2 (13.80-14.24 g/dL) vs. D8	1,888 (9.9)	1.35 (1.25, 1.45)	**<0.0001**	1.34 (1.24, 1.44)	**<0.0001**	1.42 (1.30, 1.54)	**<0.0001**
D3 (14.25-14.54 g/dL) vs. D8	1,570 (8.7)	1.17 (1.08, 1.26)	**<0.0001**	1.17 (1.09, 1.27)	**<0.0001**	1.25 (1.15, 1.37)	**<0.0001**
D4 (14.55-14.80 g/dL) vs. D8	1,647 (8.4)	1.10 (1.02, 1.18)	**0.0184**	1.10 (1.02, 1.19)	**0.0123**	1.19 (1.10, 1.30)	**<0.0001**
D5 (14.81-15.02 g/dL) vs. D8	1,410 (8.5)	1.11 (1.02, 1.20)	**0.0132**	1.11 (1.03, 1.20)	**0.0090**	1.18 (1.08, 1.29)	**0.0003**
D6 (15.03-15.29 g/dL) vs. D8	1,556 (8.3)	1.07 (0.99, 1.15)	0.0930	1.08 (1.00, 1.17)	0.0547	1.13 (1.03, 1.23)	**0.0066**
D7 (15.30-15.51 g/dL) vs. D8	1,448 (8.1)	1.01 (0.94, 1.09)	0.7674	1.01 (0.93, 1.09)	0.8121	1.05 (0.96, 1.14)	0.3348
D8 (15.52-15.81 g/dL)	1,493 (8.2)	Reference	-	Reference	-	Reference	-
D9 (15.82-16.23 g/dL) vs. D8	1,533 (8.5)	1.02 (0.95, 1.10)	0.5966	1.02 (0.94, 1.10)	0.6471	1.02 (0.93, 1.11)	0.7164
D10 (>16.23 g/dL) vs. D8	1,781 (9.7)	1.11 (1.03, 1.20)	**0.0063**	1.08 (1.00, 1.17)	**0.0412**	1.03 (0.95, 1.13)	0.4407
**Women¶ (*n*=204,429)**	10,570 (5.2)						
D1 (<12.40 g/dL) vs. D8	1,400 (6.9)	1.98 (1.82, 2.16)	**<0.0001**	1.87 (1.71, 2.04)	**<0.0001**	1.98 (1.78, 2.20)	**<0.0001**
D2 (12.40-12.79 g/dL) vs. D8	913 (4.6)	1.25 (1.13, 1.37)	**<0.0001**	1.23 (1.12, 1.36)	**<0.0001**	1.31 (1.17, 1.47)	**<0.0001**
D3 (12.80-13.09 g/dL) vs. D8	961 (4.5)	1.16 (1.06, 1.27)	**0.0017**	1.16 (1.06, 1.27)	**0.0019**	1.26 (1.13, 1.41)	**<0.0001**
D4 (13.10-13.30 g/dL) vs. D8	909 (4.3)	1.08 (0.98, 1.18)	0.1268	1.07 (0.98, 1.18)	0.1476	1.12 (1.001, 1.25)	**0.0482**
D5 (13.31-13.52 g/dL) vs. D8	871 (4.4)	1.05 (0.95, 1.15)	0.3339	1.05 (0.95, 1.15)	0.3394	1.16 (1.03, 1.29)	**0.0124**
D6 (13.53-13.75 g/dL) vs. D8	898 (4.4)	1.03 (0.93, 1.13)	0.5988	1.03 (0.94, 1.13)	0.5278	1.07 (0.95, 1.20)	0.2597
D7 (13.76-13.99 g/dL) vs. D8	905 (4.5)	0.99 (0.90, 1.09)	0.8956	0.99 (0.90, 1.09)	0.8412	1.04 (0.93, 1.16)	0.5234
D8 (14.00-14.28 g/dL)	1,004 (4.7)	Reference	-	Reference	-	Reference	-
D9 (14.29-14.67 g/dL) vs. D8	1,161 (5.7)	1.14 (1.04, 1.24)	**0.0046**	1.13 (1.03, 1.23)	**0.0092**	1.09 (0.98, 1.21)	0.1224
D10 (>14.67 g/dL) vs. D8	1,548 (7.6)	1.42 (1.30, 1.54)	**<0.0001**	1.36 (1.25, 1.48)	**<0.0001**	1.32 (1.19, 1.46)	**<0.0001**

*With BMI as additional covariate in all models. †Model 1 adjusted for age and age-squared. ‡Model 2 additionally adjusted for smoking and alcohol consumption. §Model 3 additionally adjusted for physical activity, highest educational attainment, and comorbidities. ¶In women, menopausal status was included as covariate.
